# Characterization of Genetic Landscape and Novel Inflammatory Biomarkers in Patients With Adult‐Onset Still's Disease

**DOI:** 10.1002/art.43054

**Published:** 2024-12-16

**Authors:** Joanne Topping, Leon Chang, Fatima Nadat, James A. Poulter, Alice Ibbotson, Samuel Lara‐Reyna, Christopher M. Watson, Clive Carter, Linda P. Pournara, Jan Zernicke, Rebecca L. Ross, Catherine Cargo, Paul A. Lyons, Kenneth G. C. Smith, Francesco Del Galdo, Jürgen Rech, Bruno Fautrel, Eugen Feist, Michael F. McDermott, Sinisa Savic, the ImmunAID Consortium

**Affiliations:** ^1^ University of Leeds Leeds United Kingdom; ^2^ St James's University Hospital Leeds United Kingdom; ^3^ Charité Universitätsmedizin Berlin Berlin Germany; ^4^ University of Leeds and National Institute for Health and Care Research Leeds Biomedical Centre, Chapel Allerton Hospital Leeds United Kingdom; ^5^ University of Cambridge Cambridge United Kingdom; ^6^ Friedrich‐Alexander University Erlangen‐Nürnberg and Universitätsklinikum Erlangen Erlangen Germany; ^7^ Sorbonne University, AP‐HP, Pitié‐Salpêtrière Hospital, INSERM UMR 1136 Paris France; ^8^ Otto‐von‐Guericke‐University Magdeburg, Magdeburg, Germany, and Helios Clinic Gommern Germany; ^9^ University of Leeds, St James's University Hospital, and National Institute for Health and Care Research Leeds Biomedical Centre, Chapel Allerton Hospital Leeds United Kingdom

## Abstract

**Objective:**

Adult‐onset Still disease (AOSD) is a systemic autoinflammatory disorder (AID) of unknown etiology. Genetic studies have been limited. Here, we conducted detailed genetic and inflammatory biomarker analysis of a large cohort with AOSD to investigate the underlying pathology and identify novel targets for potential treatment.

**Methods:**

We investigated AOSD cases (n = 60) for rare germline and somatic variants using whole exome sequencing with virtual gene panels. Transcriptome profiles were investigated by bulk RNA sequencing whole blood. Cytokine profiling was performed on an extended patient cohort (n = 106) alongside measurements of *NLRP3* inflammasome activation using a custom assay and type I interferon (IFN) score using a novel method.

**Results:**

We observed higher than expected frequencies of rare germline variants associated with monogenic AIDs in AOSD cases (AOSD 38.4% vs healthy controls [HCs] 20.4%) and earlier onset of putative somatic variants associated with clonal hematopoiesis of indeterminate potential. Transcriptome profiling revealed a positive correlation between Still Activity Score and gene expression associated with the innate immune system. ASC/*NLRP3* specks levels and type I IFN scores were significantly elevated in AOSD cases compared with HCs (*P* = 0.0001 and 0.0015, respectively), in addition to several cytokines: interleukin (IL)‐6 (*P* < 0.0001), IL‐10 (*P* < 0.0075), IL‐12p70 (*P* = 0.0005), IL‐18 (*P* < 0.0001), IL‐23 (*P* < 0.0001), IFN‐α2 (*P* = 0.0009), and IFNγ (*P* = 0.0002).

**Conclusion:**

Our study shows considerable genetic complexity within AOSD and demonstrates the potential utility of the ASC/*NLRP3* specks assay for disease stratification and targeted treatment. The enriched genetic variants identified may not by themselves be sufficient to cause disease, but may contribute to a polygenic model for AOSD.

## INTRODUCTION

Adult‐onset Still disease (AOSD) is a systemic autoinflammatory disorders (AID) of unknown etiology. Typically presenting with prolonged intermittent fevers, arthralgias, and evanescent rash—any system can be affected.^1^ It is most often diagnosed following exclusion of infection, malignancy, or other inflammatory rheumatologic diseases, and patients must fulfill classification criteria to confirm the diagnosis. The criteria by Yamaguchi et al is the most commonly used.^2^

AOSD can be stratified into different subtypes depending on disease course or predominant clinical features. In the chronic disease course, patients either have systemic disease characterized by high fevers, rash, and multiorgan involvement, or a predominantly articular disease manifesting with inflammatory joint problems. There is frequent overlap among these presentations.^3^

AOSD is presumed to have a polygenic basis, but there is genetic and clinical overlap with monogenic autoinflammatory disorders (mAIDs). A systematic review of 162 patients with AOSD and systemic‐onset juvenile idiopathic arthritis (sJIA; sJIA is considered the same disease as AOSD but with childhood onset) demonstrated that 51 patients (31.4%) carried at least one genetic variant associated with specific mAIDs (predominantly hereditary fever syndromes).^4^ The link was further supported by the observation that biologic therapies targeting proinflammatory cytokines, interleukin (IL)‐1β or IL‐6, both key to mAIDs pathogenesis, are highly effective in treating AOSD.^5^, ^6^

Recent genetic developments in AID and the observation that patients with severe or resistant AOSD respond to treatment such as JAK inhibitors (JAKi), provides insight into disease pathogenesis. For example, somatic gain‐of‐function (GOF) mutations in autoinflammatory genes such as *NLRP3 and NLRC4*, which arise within bone marrow and are restricted to myeloid lineage, result in spontaneous activation of the respective inflammasomes with subsequent excessive release of IL‐1β and IL‐18 and late‐onset autoinflammatory disorders (AIDs).^7^, ^8^ Such cases are phenotypically indistinguishable from those with inherited (constitutional) mutations, despite only a small proportion of myeloid cells carrying the somatic mutations. Similarly, VEXAS syndrome (vacuoles, E1 enzyme, X‐linked, autoinflammatory, somatic), caused by a somatic mutation in *UBA1*, and with disease pathogenesis linked to mutated and highly inflammatory myeloid cells, has clinical features that overlap with AOSD.^9^ There is also increasing recognition of the overlap between inflammatory conditions and myelodysplasia, with around 50% of patients with myelodysplastic syndrome (MDS) having autoinflammatory complications.^10^ Furthermore, in the premalignant bone marrow state clonal hematopoiesis of indeterminant potential (CHIP), which often precedes MDS, certain somatic mutations in bone hematopoietic stem cells have been associated with *NLRP3* inflammasome activation, release of IL‐1β, and a general proinflammatory state.^11^ These phenomena are more frequent in the elderly, with one case series indicating a second incidence peak of AOSD in this population,^12^ although the diagnosis is some of these cases was uncertain. Finally, it is increasingly recognized that some patients with resistant disease, and disease complicated by macrophage activation syndrome, are responsive to JAKi.^13^, ^14^ This suggests innate immune pathways beyond those involving IL‐1β and IL‐6, such as type I interferon (IFN), may be relevant in AOSD.^15^

We investigated three cohorts of well‐characterized patients with AOSD at different stages of disease progression and treatment response. We sought to determine its genetic basis and establish whether low‐prevalence somatic variants in candidate genes contribute to disease pathogenesis and the activation of specific inflammatory pathways linked with AOSD pathogenesis. We used bespoke assays to detect *NLRP3* inflammasome activation and measure serological IFN signature. Finally, we compared the mutation profiles of AOSD cases with defining disease signatures and inflammatory biomarkers to determine the potential functional relevance of rare genetic variants on AOSD expression.

## MATERIALS AND METHODS

Please refer to the Supplemental Materials for in‐depth descriptions of experimental methodology, data acquisition, and statistical analysis. In summary, 106 patients with AOSD were recruited from three separate cohorts (AOSD#1, AOSD#2, and AOSD#3); demographics are listed in Supplementary Table 1. Whole exome sequencing (WES) was performed on DNA samples extracted from whole blood (n = 60 from AOSD#2 and AOSD#3, mean depth 142×). Genetic variants were identified using in‐house bioinformatic pipelines and a custom panel of 139 genes divided into three subcategories in association with CHIP, autoinflammation, and type I interferonopathies (Supplementary Table 2). Enrichment analyses of germline variants were performed using a Fisher's exact test for each variant and the recorded prevalence in the European non‐Finnish population in the Genome Aggregation Database (gnomAD) for comparison. We accepted statistical significance at a *P* value adjusted for multiple testing with Bonferroni correction (*P* < 0.05/variant count). Bulk RNA sequencing (RNAseq) was performed on whole blood samples from AOSD cases (AOSD#2, n = 27) and healthy controls (HCs) (HCs, n = 10). Differentially expressed genes were defined as those with a log2FoldChange greater than 1 or less than −1 and an adjusted *P* value <0.001.

Functional assessments were performed on the collective AOSD cohort (AOSD#1, AOSD#2, and AOSD#3), and results were compared with other inflammatory disease and HC cohorts. We used an in‐house flow cytometry assay for quantification of antibody‐secreting cell (ASC)/*NLRP3* protein specks in patient sera (Supplementary Figure 1). The inflammatory cytokine profiles were investigated using the multiplex LEGENDplex Multi‐Analyte Flow Assay kit (Biolegend). The type I IFN score was investigated on patient sera using a custom Luminex Discovery Assay (bio‐techne, R&D systems). Full details of ImmunAID investigators are provided in the Supplementary Material.

This study was approved by the South West – Frenchay Research Ethics Committee (Research Ethics Committee reference: 20/SW/0022). Data are available upon reasonable request. Processed and raw RNAseq data is publicly available at time of publication; Gene Expression Omnibus accession number GSE244372; BioProject PRJNA1022483.

## RESULTS

### Patients with AOSD, disease, and HC demographics

Demographic data for all patients with AOSD, the disease, and HCs are shown in Supplementary Table 1. The collective AOSD cohort (n = 106) comprised 68 women and 38 men with a mean age of 39.0 years. No significant differences in age distribution were detected across cohorts between the men and women. The sample cohorts used to produce each data set will be specified at the start of each section.

### Rare germline and somatic variants identified using virtual gene panels

We compared WES data from 60 of 106 patients with AOSD (AOSD#2 n = 30; AOSD#3 n = 30) against a similar sized cohort of unrelated HCs (HC#1 n = 49, sequencing metrics available in Supplementary Table 3). Germline and somatic variant analyses were performed in parallel for each individual, and variant origins were distinguished bioinformatically. We investigated the burden of rare genetic variants in gene panels related to AIDs, CHIP, and type I interferonopathies (Supplementary Table 2). The genes within each panel are mutually exclusive.

#### Germline variant analysis

**Figure 1:**
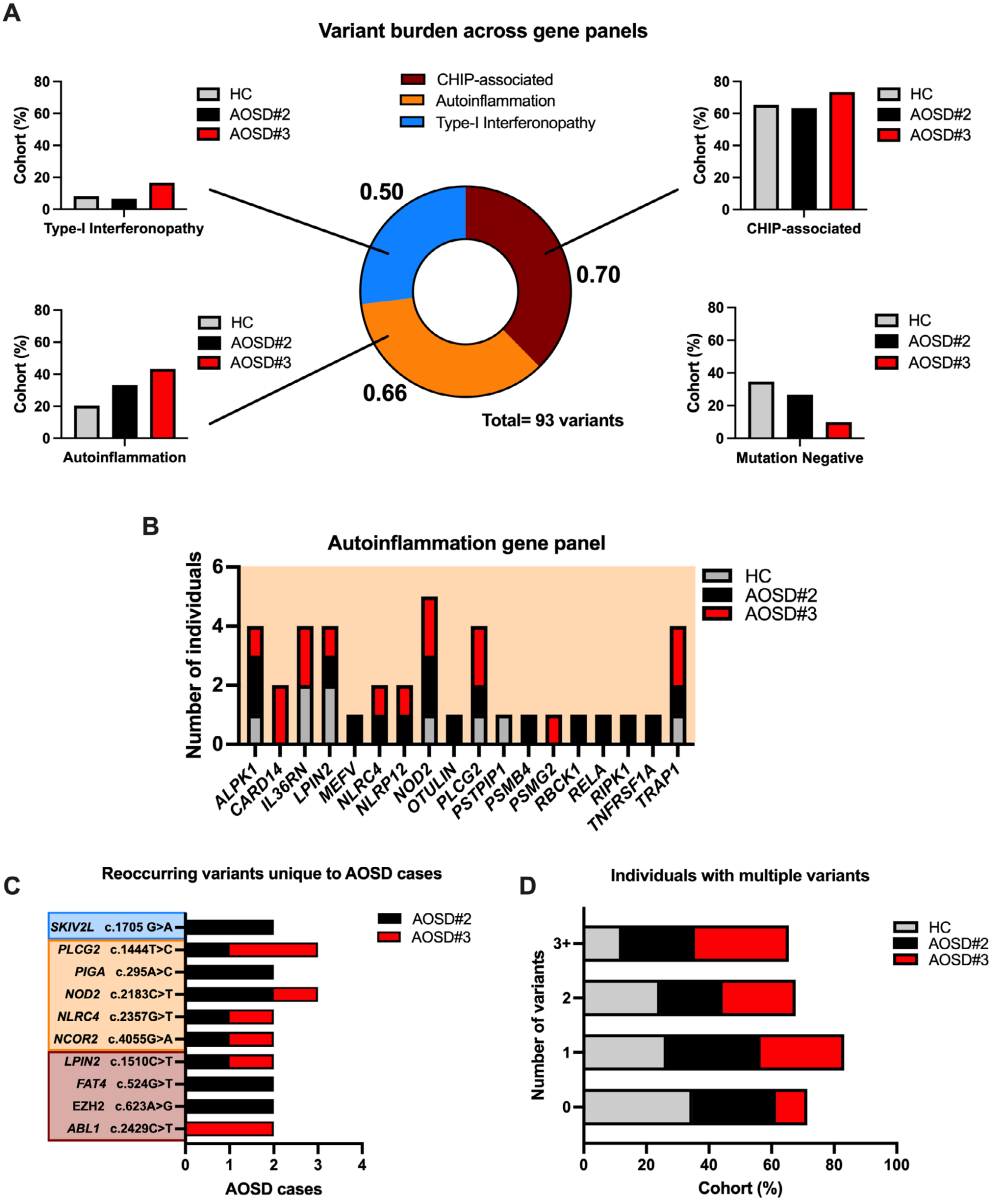
Rare germline variant analysis. (A) The number of variants identified in each panel (CHIP‐associated, autoinflammation, and type I interferonopathy) was normalized to their corresponding gene panel size (gene count). Bar charts for each segment illustrate the proportion of AOSD and HC cohorts (%) carrying a rare germline variant in each gene panel. (B) The frequency of cases carrying rare variants across genes in the autoinflammation gene panel in each cohort. (C) Summary of the rare germline variants shared by more than one AOSD case, which are color‐coded to reflect the associated gene panel: CHIP (brown), autoinflammation (orange), type I interferonopathy (blue). (D) The proportion of each cohort (%) carrying multiple variants across all gene panels. AOSD, adult‐onset Still disease; CHIP, chromatin immunoprecipitation; HC, healthy control.

In the AOSD cohort, we identified 93 rare (<1% population allele frequency), potentially pathogenic (combined annotation dependent depletion [CADD] score >20) heterozygous germline variants (89 missense, four nonsense) across 60 genes within our gene panels (Figure 1A; Supplementary Table 4). These variants were distributed across 49 of 60 AOSD cases. The number of variants identified was normalized to the number of genes within each panel, generating frequencies of 0.70, 0.66, and 0.50 variants per gene in the CHIP‐associated, autoinflammation, and type I interferonopathy panels, respectively. The variant profiles between the AOSD cohort (93 variants) and HCs (57 variants) were mostly unique with only four variants in common. No biallelic variants were identified in any samples.

We compared cohorts AOSD#2 and AOSD#3 with HCs separately to observe differences in variant burden. Cases from AOSD#3, which are treatment‐resistant, had the highest variant burden across each gene panel overall. This was most evident in the autoinflammation gene panel in which variants were twice as prevalent in AOSD cases in comparison with HCs (AOSD#3 43.4%; AOSD#2 33.3%; HCs 20.4%). Genes *ALPK1*, *PLCG2*, *TRAP1*, and *NOD2* had the highest variant frequency within this panel among AOSD cases (Figure 1B), and reoccurring variants of interest include *NOD2* c.2183C>T and *PLCG2* c.1444T>C; each were identified in three separate AOSD cases (Figure 1C).

A significant proportion of AOSD cases (31 of 60) carried multiple variants (≥2) and cohort AOSD#3 (30.0%, 9 of 30) had the largest proportion of cases carrying ≥3 variants (Figure 1D). Approximately one‐ third of the HC cohort were mutation‐negative within our gene panels (34.7%, 17 of 49), whereas only 26.7% (8 of 30) and 10.0% (3 of 30) were mutation‐negative among AOSD#2 and AOSD#3 cases, respectively.

#### Variant enrichment analysis

We conducted enrichment analyses on 142 variants found uniquely to either AOSD (89 of 93 variants) or HC cases (53 of 57 variants) and accepted a statistical significance at *P* value <3.52 × 10^−4^ after Bonferroni correction for multiple testing. Comparing with the recorded prevalence of each variant in the European non‐Finnish population of gnomAD v4.0.0 (590,031 individuals), we identified that 15 of 89 variants were significantly overrepresented within the AOSD cohort across 13 cases. Two enriched variants were found in genes from the autoinflammation panel (*ALPK1*, *PSMG2*) and the remaining 13 variants were in CHIP‐associated genes (Supplementary Table 4). We identified only 3 of 53 variants enriched in HCs. Despite each cohort having similar proportions of germline variants in CHIP‐associated genes (AOSD#2 63.3%; AOSD#3 73.3%; HCs 66.3%), 22.0% (13 of 59 variants) of these were enriched in AOSD cases, whereas only 4.7% (2 of 43 variants) were enriched in HCs.

All variants were cross‐referenced with the Infevers database (access date February 2024), which is an established registry of genetic variants associated with mAIDs. Nine variants were previously reported in Infevers, and two variants were classified as pathogenic: *TNFRSF1A* c.242G>A (P.(C81Y)) and *RIPK1* c.1934C>T (P.(T645M)); each was identified in a separate AOSD case in the heterozygous state. The genetics of autoinflammatory conditions associated with *RIPK1* are complex. The loss‐of‐function variants such as T645M are inherited either as homozygous or compound heterozygous states and will cause predominantly immunodeficiency with some immunodysregulation,^16^ whereas GOF variants, which stop the inactivation of RIPK1, are highly penetrant and are associated with a purely autoinflammatory phenotype.^17^ In this case, we have not identified a plausible second‐hit or GOF variants following manual scrutiny of the sequence data. Therefore, it is highly unlikely that the T645M variant alone is sufficient to be disease‐causing, and its contribution to overall AOSD phenotype is uncertain. In contrast, the *TNFRSF1A* p.C81Y mutation is an established autosomal dominant cause of TNF receptor‐associated syndrome (TRAPS), with several well‐characterized cases reported in the literature.^18^, ^19^ A full case description of this patient is provided in Supplementary Materials. Furthermore, we identified a heterozygous *MVK* c.1129G>A (P.(V377I)) variant in one AOSD case that was also classified as pathogenic on Infevers. However, because of the low CADD score (15.1) this variant was omitted from further analysis (CADD >20 threshold).

#### Somatic variant analysis

**Figure 2:**
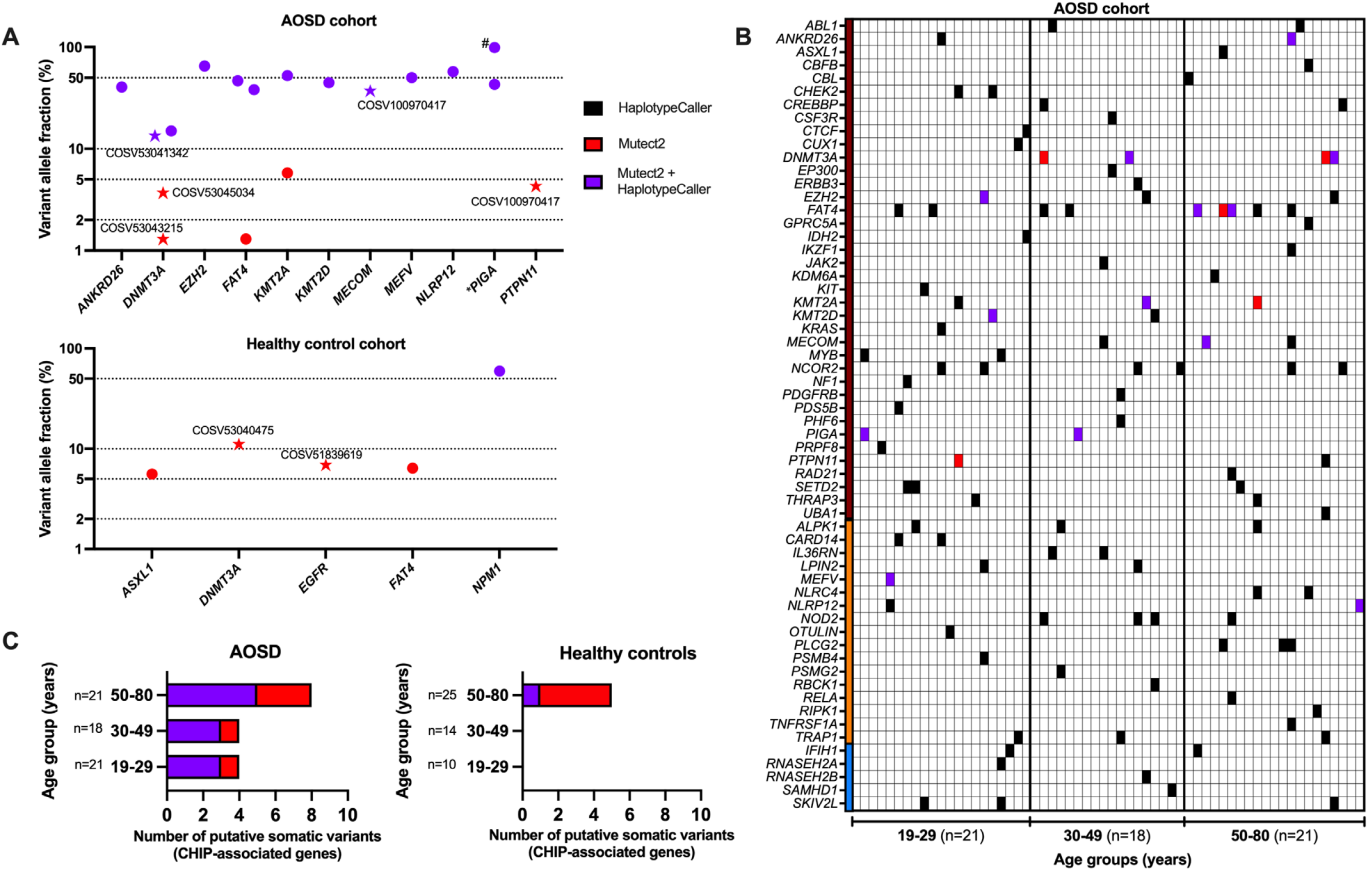
Somatic variant analysis in AOSD. Variants are color coded according to the variant calling tool used for identification (black: HaplotypeCaller [putative germline]; red: Mutect2 [putative somatic]; purple: found by both HaplotypeCaller and Mutect2 [uncertain origin]). (A) An overview of VAF across all putative somatic variants identified by Mutect2. Genes located on an X chromosome are highlighted *; biological males with high VAF % on these genes are annotated with #. Somatic variants previously reported in COSMIC are presented as stars annotated with their COSV numbers. (B) Mutational landscape summary of all putative somatic and germline variants identified in AOSD cases within the preselected gene panels (*y*‐axis gene panel: CHIP = dark red; autoinflammation = orange; type I interferonopathy = blue) in age order (*x*‐axis). (C) The distribution of putative somatic variants identified in CHIP‐associated genes across each age group. AOSD, adult‐onset Still disease; CHIP, clonal hematopoiesis of indeterminant potential; COSMIC, Catalog of Somatic Mutations in Cancer; COSV, genomic mutation identifier; VAF, variant allele fractions.

We applied the somatic variant caller Mutect2 to our exome data and filtered out variants under 69× total read depth—a lower threshold determined by a somatic variant validated by digital polymerase chain reaction in HCs (Supplementary Figure 2). We also filtered the remaining variants by CADD >20 and ≥5 variant read depth (exception allowed to variants previously confirmed as somatic in the Catalog of Somatic Mutations in Cancer [COSMIC]). This identified 18 putative somatic variants (Supplementary Table 5) across 18 of 60 AOSD cases (30.0%) and 5 variants in 5 of 49 HCs (10.2%). The burden of putative somatic variants was highest in *DNMT3A* and *FAT4* from the CHIP‐associated gene panel (Figure 2A). Joint analysis with the previously discussed germline variant data identified 14 AOSD cases (23.3%) carrying a combination of at least one germline and one putative somatic variant within our targeted gene panels (Figure 2B). This combination was only found in three HC samples (6.1%) in comparison. Putative somatic variant counts were also similar between cohorts AOSD#2 (11 cases) and AOSD#3 (12 cases).

#### Age‐related prevalence of CHIP‐associated genetic variants in AOSD

The incidence of CHIP increases with age and is relatively common above 70 years old.^20^ To establish whether the prevalence of CHIP in AOSD is also age‐dependent, putative somatic variants found in CHIP‐associated genes were analyzed. To explore variant distribution with increasing age, both HC and AOSD cohorts were partitioned into three age brackets: 19 to 29, 30 to 49, and 50 to 80 years (Figure 2C). Overall, the AOSD cohort had a greater frequency of putative somatic variants associated with CHIP across all age groups in comparison to HCs, which were confined largely to the group aged 50 to 80 (Figure 2C).

### Candidate gene identification following transcriptome analysis

**Figure 3:**
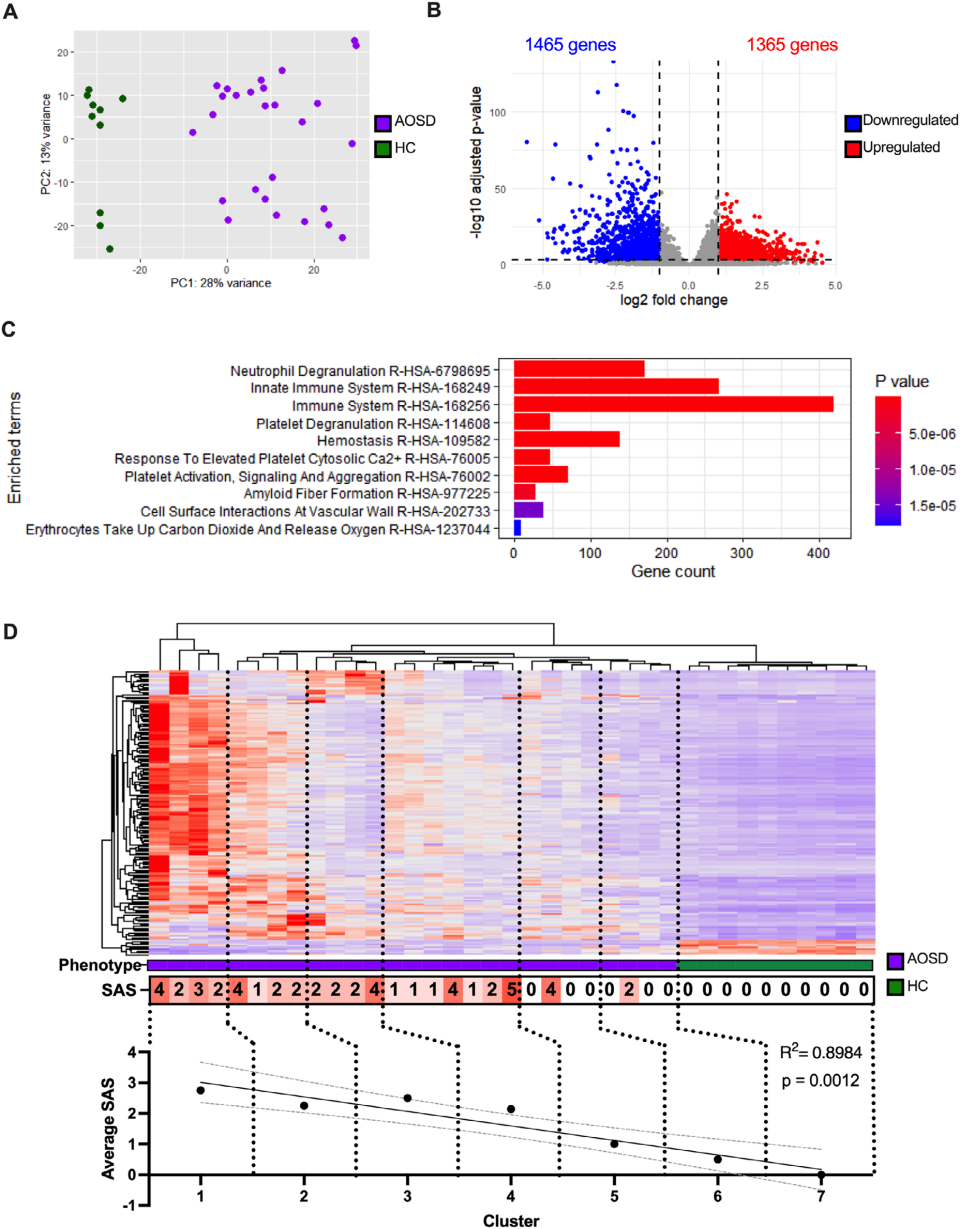
Transcriptome analysis of AOSD cases: (A) Principal component analysis plot illustrating the relationship between AOSD cases and HCs. (B) Volcano plot highlighting the log2 fold change and ‐log10 adjusted *P* value of 2,830 differentially expressed genes in AOSD cases. (C) Pathway enrichment analysis performed through Enrichr using terms from the Reactome pathway database, enriched pathway terms are arranged in order of *P* value. (D) The relative expression of 170 genes shared among the top three pathways are displayed in a heatmap. The gene expression profiles of AOSD (purple) and healthy control (green) samples are grouped into hierarchical clusters and the average SAS of each cluster was recorded. Linear regression analysis showed significant positive correlation between SAS scores and increasing gene expression. AOSD, adult‐onset Still disease; HC, healthy control; SAS, Still Activity Score.

To identify additional candidate genes of interest, we used transcriptome data generated from 27 patients with AOSD (AOSD#2) and 10 HCs (HC#2). Compared with HCs, 2,830 genes were differentially expressed in the AOSD cohort (Figure 3). Pathway and gene ontology analyses identified significant enrichment in genes associated with neutrophil degranulation and the innate immune system (Figure 3C). We identified 170 genes shared between the top three ranked pathway terms, the majority of which (162 of 170 genes) had up‐regulated expression in the AOSD cohort. This gene expression signature may be driven by the neutrophilia associated with active AOSD, and the variability within these pathways, observed through the heatmap, we hypothesized would reflect differences in the patient phenotype severity (Figure 3D).

To investigate this, we plotted hierarchal sample clusters of similar gene expression profile (dictated by the dendrogram) against the Still Activity Scores (SAS) of samples within each cluster (1–7). The SAS is a clinical disease activity composite score calculated using a combination of clinical presentations and laboratory findings (fever, arthralgia, neutrophilia, and ferritin levels).^21^ The highest SAS mean (2.75) was in cluster 1, which contained samples with the highest gene expression within the enriched pathways. A gradual reduction in mean SAS was observed across clusters, and linear regression analysis revealed a significant correlation (*P* = 0.0012, *R* ^2^ = 0.898), confirming a positive relationship between SAS and gene expression within these pathways.

We targeted these genes using our variant identification pipeline and identified an additional 87 rare germline variants across 61 of 170 genes. Only six variants were also present in the HC cohort. Enrichment analysis on the remaining 81 variants unique to AOSD identified 12 of 81 variants were significantly overrepresented across 12 of 60 (20%) AOSD cases (Supplementary Table 6).

### ASC specks profiling in AOSD

The pathogenesis of AOSD has been linked to inappropriate activation of the *NLRP3* inflammasome and the release of proinflammatory cytokines.^22^ We have developed a novel assay to detect components of the inflammasome (the apoptosis‐associated speck‐like protein containing a caspase recruitment domain (ASC) and *NLRP3*, as a complex) that are released into serum following inflammasome activation and pyroptotic cell death. ASC is an adaptor protein also found in other inflammasome complexes (*NLRP1*, *NLRC4*, Pyrin, absent in melanoma 2 [AIM2], and *NLRP10*); however, because our assay detects ASC complexed with *NLRP3*, detection of these protein complexes (specks) is indicative of *NLRP3* inflammasome activation. Using this assay, we studied *NLRP3* inflammasome activation in sera from patients with AOSD and compared these data with HCs and various recognized canonical mAIDs and polygenic AIDs.

**Figure 4:**
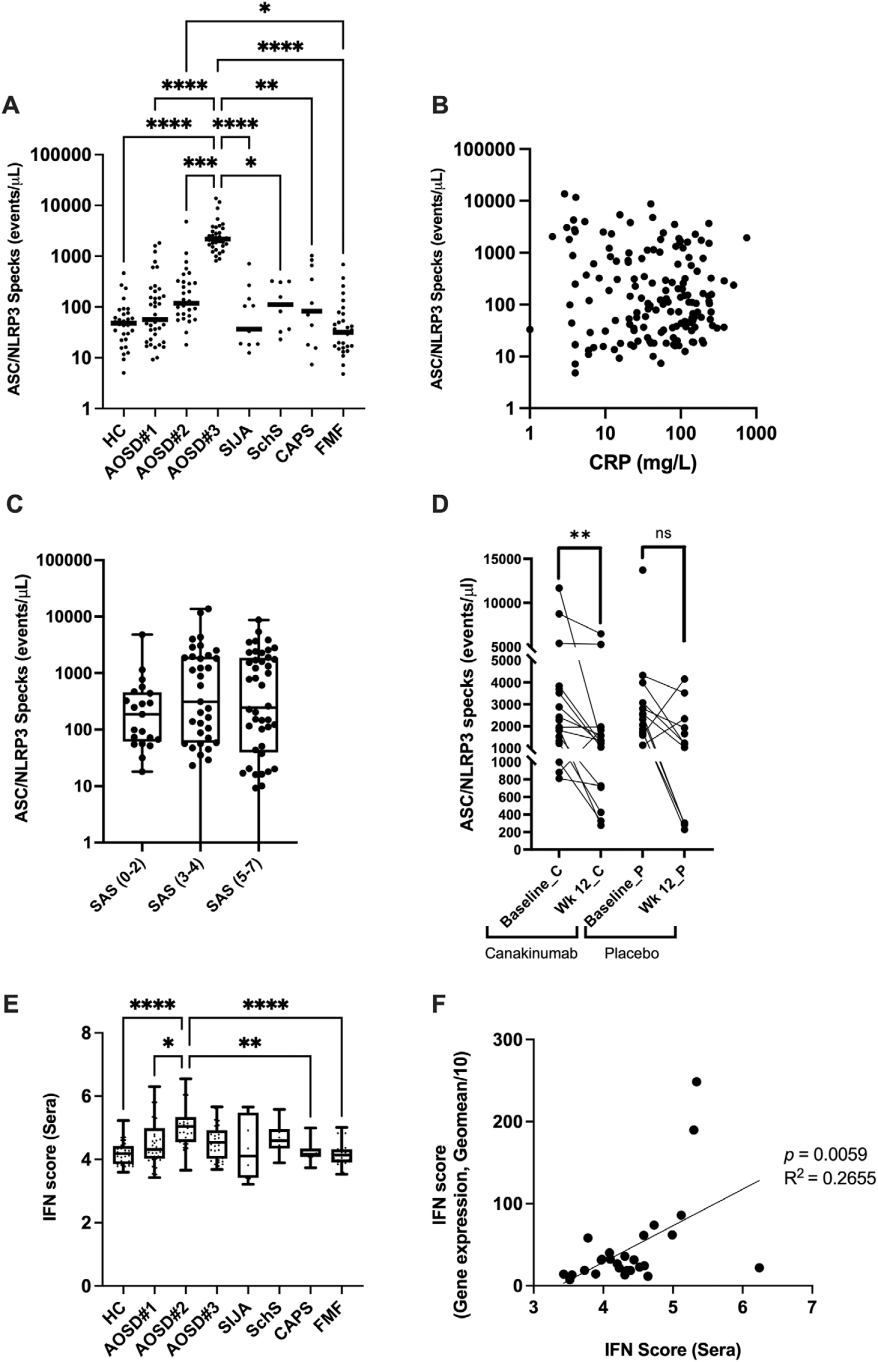
ASC/*NLRP3* specks and IFN activity levels in AOSD and disease control cohorts: (A) ASC/*NLRP3* specks in healthy controls (HC, n= 30) compared with AOSD cohort subsets (AOSD#1 (n = 39), AOSD#2 (n = 30) and AOSD#3 (n = 34), sJIA (n = 12), SchS (n = 10), CAPS (n = 11) and FMF (n = 31) cohorts. The weighted bars represent median values. (B) Scatterplot comparing ASC/*NLRP3* specks to CRP (mg/L) taken from all disease cohorts. (C) ASC/*NLRP3* specks comparisons within the AOSD cases, categorized by mild (0–2), moderate (3–4) and severe (5–7) Still Activity Score (SAS). (D) Change in ASC/*NLRP3* specks in AOSD cases following treatment with canakinumab or placebo. (E) Boxplots comparing IFN scores (Sera) between HCs and cases from each disease cohort. (F) Comparison of IFN scores calculated from sera against IFN scores calculated from the gene expression of interferon response genes (geomeans/10), analyzed by linear regression. Statistically significant pairwise comparisons are annotated with * =*P* < 0.05; ** =*P* < 0.01; *** =*P* < 0.001; **** =*P* < 0.0001. AOSD, adult‐onset Still disease; ASC, antibody‐secreting cell; CAPS, cryopyrin‐associated periodic syndrome; CRP, C‐reactive protein; FMF, familial Mediterranean fever; HC, healthy control; IFN, interferon; SchS, Schnitzler syndrome; sJIA, systemic juvenile idiopathic arthritis.

Overall, the combined AOSD cohort had significantly higher levels of ASC/*NLRP3* specks (236.5 [56.2–1,693.0] events/μL, median [interquartile range (IQR)]) compared with HCs (*P* = 0.0001, 48.1 [22.9–79.1] events/μL) and cases of familial Mediterranean fever (FMF) (*P* < 0.0001, 31.6 [15.7–73.5] events/μL). This was most prominent in treatment‐resistant patients from cohort AOSD#3 who also had higher ASC/*NLRP3* specks in comparison with patients with AIDs typically associated with *NLRP3* inflammasome activation, such as cryopyrin‐associated periodic syndrome (CAPS) and Schnitzler syndrome (Figure 4A).

To determine if ASC/*NLRP3* specks levels might have diagnostic or prognostic value in AOSD, we compared ASC/*NLRP3* specks with C‐reactive protein (CRP) levels and SAS (divided into mild [0–2, n = 21], moderate [3–4, n = 35], and severe SAS [5–7, n = 47]). No statistically significant correlations were identified between ASC/*NLRP3* specks and either of these variables (Figure 4B and C). Correlations between CRP and ASC specks across each condition is available in Supplementary Figure 3. We subsequently focused on patients with particularly high levels of ASC/*NLRP3* specks from the AOSD#3 cohort. These patients were treatment‐resistant, predominantly diagnosed with the articular subtype of the disease, and participants in the Canakinumab for Treatment of Adult‐Onset Still's Disease to Achieve Reduction of Arthritic Manifestation trial (for details, please see the Materials and Methods section). We tested samples taken before and following treatment with either canakinumab or placebo and found that ASC/*NLRP3* specks levels were significantly reduced (*P* < 0.01) following treatment with canakinumab but not placebo (Figure 4D), which suggests that this biomarker could be used to monitor treatment response to anti–IL‐1 therapy.

In parallel with ASC/*NLRP3* protein specks, we measured levels of ASC (only) specks. We reasoned that this biomarker might be informative around the activation of other inflammasome complexes that use this adaptor protein. Overall, the data from ASC (only) specks closely resembled the ASC/*NLRP3* data with cases from cohort AOSD#3, having significantly greater ASC (only) specks compared with HCs and cases with other autoinflammatory conditions (Supplementary Figure 4).

### Interferon activity score in AOSD

Given the favorable response to JAKi observed in some patients with AOSD, we examined the role of type I IFN in disease pathogenesis using a novel assay to measure serum levels of several IFN‐responsive chemokines, CCL2, CCL8, CCL19, CXCL10, and CXCL11, and combined these levels into a serological IFN score. The utility of this assay was previously demonstrated in other inflammatory diseases for which type I IFNs are thought to be important.^23^, ^24^ Initial measurements of chemokine levels in the serum showed significantly elevated CXCL10 (*P* = 0.0005) and CXCL11 (*P* < 0.0001) levels in AOSD cases in comparison with HCs (Supplementary Figure 5). After converting raw chemokine data (CXCL10, CCL2, CCL8, CCL19, CXCL11) into a combined IFN score, we observed significantly higher type I IFN scores overall in patients with AOSD compared with HCs (4.6 [4.2–5.1] vs 4.2 [3.9–4.4], median [IQR], *P* = 0.0015) and to patients with non–type I IFN‐dependent AIDs, such as FMF (*P* = 0.0003, 4.1 [3.9–4.3], median [IQR]). Within the AOSD cohort, cases from AOSD#2 had the highest type I IFN scores, which were significantly higher than HCs, AOSD#1 cases, and those with CAPS and FMF (Figure 4E). We next compared our serological‐based IFN score with a gene expression‐based score. For each AOSD case (cohort AOSD#2), we correlated the IFN scores calculated from sera to the IFN scores calculated from whole blood gene expression (28‐IFN response genes generated from RNAseq).^25^ Linear regression analysis identified a positive correlation between both IFN score calculation methods (*P* = 0.0059, *R* ^2^ = 0.2655), providing validity to our novel assay (Figure 4F).

### Cytokine profiling

**Figure 5:**
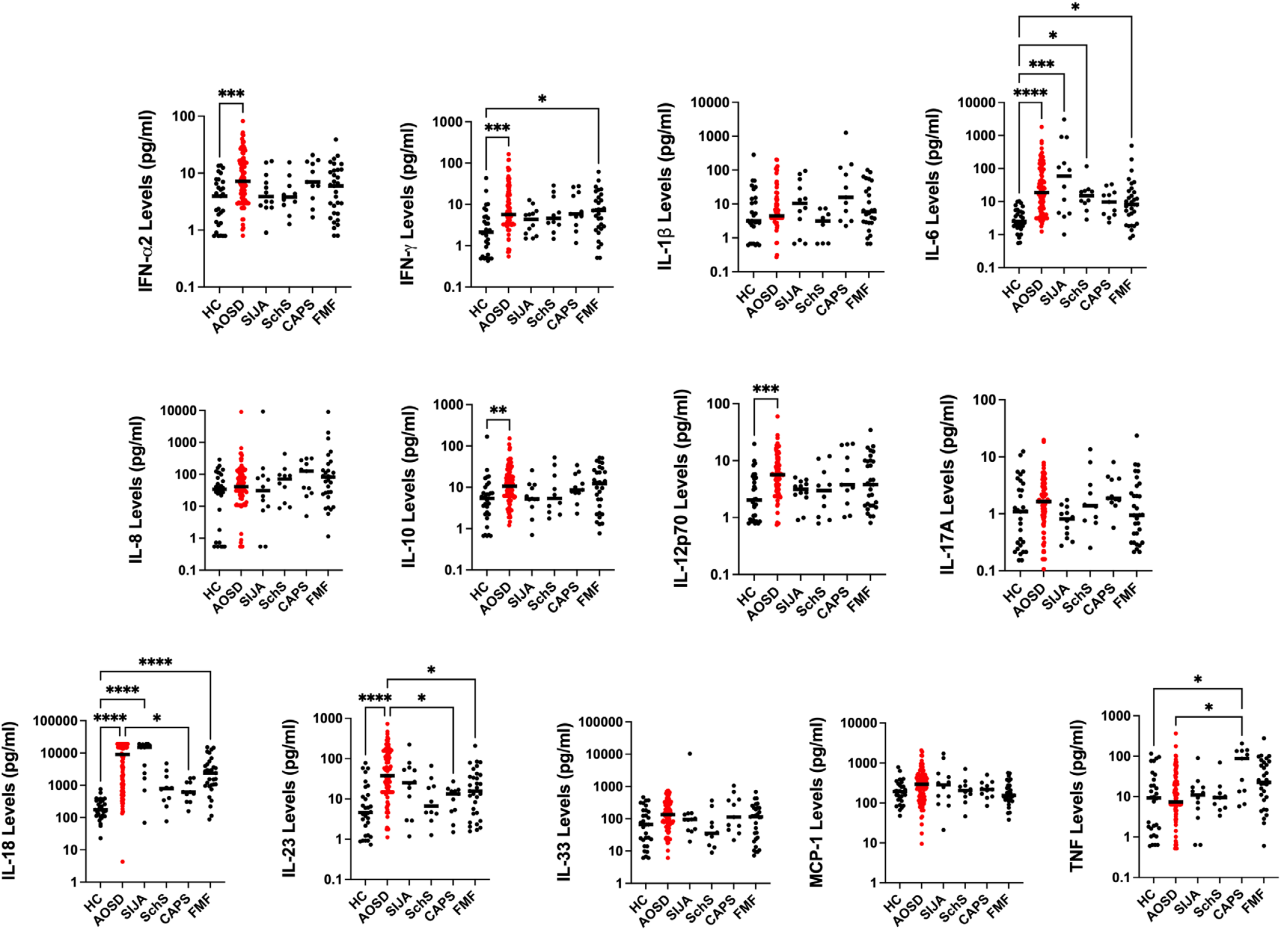
Cytokine profile in patients with AOSD, disease, and healthy controls: The serum cytokine levels for 13 targets are displayed on dot plots, comparing healthy controls to AOSD, sJIA, SchS, CAPS, and FMF cases. AOSD cases are highlighted in red, weighted bars represent median values, and statistically significant pairwise comparisons are annotated with asterisks. * =*P* < 0.05; ** =*P* < 0.01; *** =*P* < 0.001; **** =*P* < 0.0001. AOSD, adult‐onset Still disease; CAPS, cryopyrin‐associated periodic syndrome; FMF, familial Mediterranean fever; HC, healthy control; IFN, type I interferon; IL, interleukin; SchS, Schnitzler syndrome; sJIA, systemic juvenile idiopathic arthritis.

Compared with HCs, levels of IFN‐α2 (*P* = 0.0009), IFN‐γ (*P* = 0.0002), IL‐6 (*P* < 0.0001), IL‐10 (*P* < 0.0075), IL‐12p70 (*P* = 0.0005), IL‐18 (*P* < 0.0001), and IL‐23 (*P* < 0.0001) were significantly elevated in the AOSD cohort (Figure 5). IL‐18 and IL‐23 levels in AOSD cases were significantly elevated compared with patients with CAPS (*P* = 0.0276 and 0.0283, respectively). Interestingly, patients with AOSD (9,013 [886–18,209] pg/mL) and sJIA (15,000 [1,819–18,826] pg/mL) had similarly elevated levels of IL‐18 levels, in keeping with previously published data and the recent recognition that these disorders represent the same condition, presenting at different ages.^26^ The significantly elevated IFN‐α2 levels observed in patients with AOSD, in combination with the elevated IFN activity scores previously described, suggest that type I IFN drive might be important in disease pathogenesis for at least some patients with AOSD.

### Correlation between rare genetic variants and markers of inflammation

**Figure 6:**
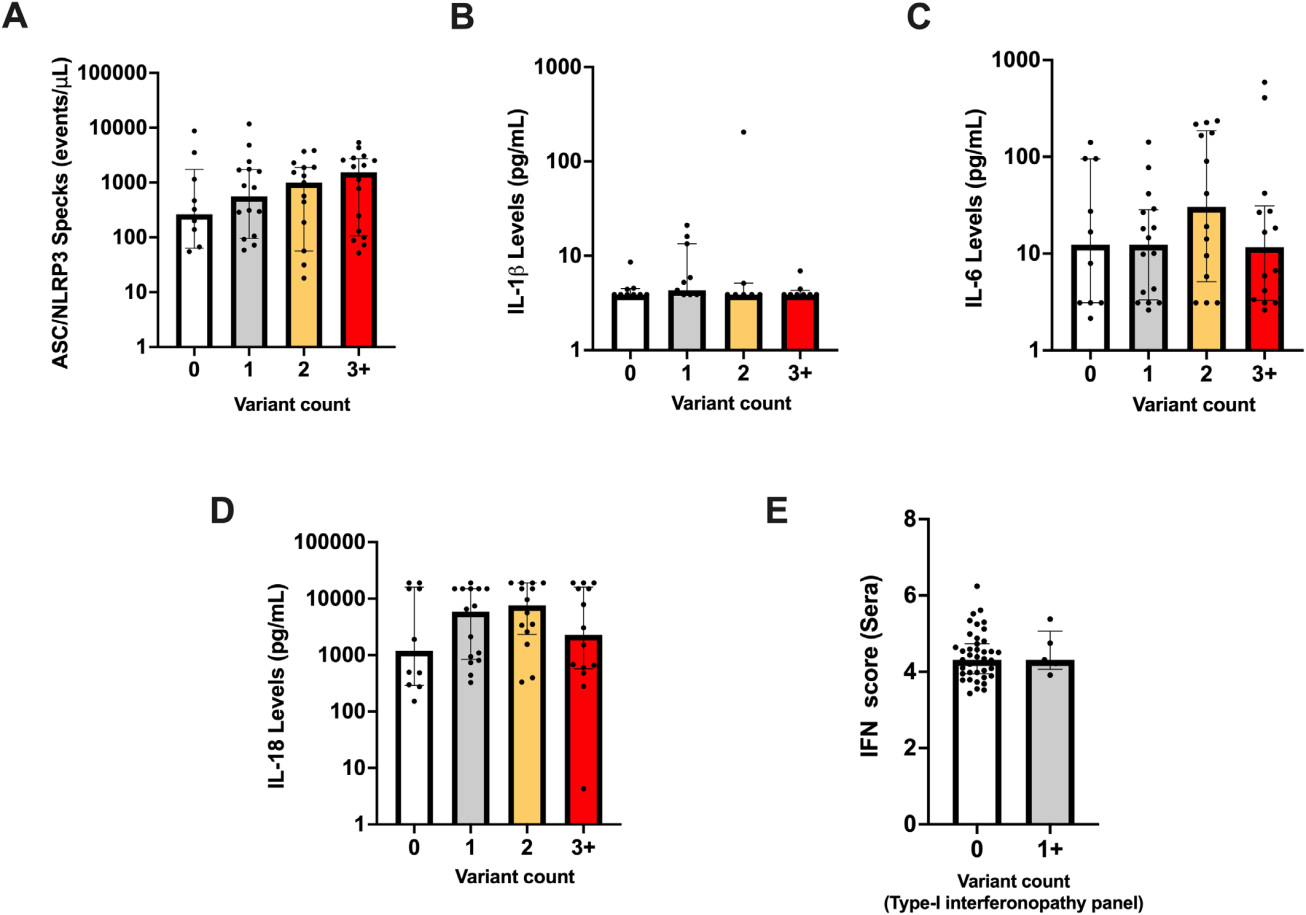
Correlation between rare genetic variant counts against biomarker levels: Bar charts (median, IQR) illustrating (A) ASC/*NLRP3* specks; (B) IL‐1β; (C) IL‐6; and (D) IL‐18 levels (pg/mL) in AOSD cases carrying 0, 1, 2, and 3+ variants (germline and/or putative somatic) within our preselected gene panels. (E) IFN scores in AOSD cases with and without a variant in the type I interferonopathy gene panel. AOSD, adult‐onset Still disease; ASC, antibody‐secreting cell; IFN, interferon; IL, interleukin; IQR, interquartile range.

To investigate any additive proinflammatory effect from multiple rare variants, we examined the relationship between variant count on ASC/*NLRP3* specks and selected cytokine levels (IL‐1β, IL‐6, and IL‐18). A modest increase in ASC/*NLRP3* specks was observed with increasing variant count, but these differences were not statistically significant (Figure 6A). This might be due to a combination of insufficient sample size (mutation‐negative AOSD cases) and high data variability. We further stratified this analysis for genes associated with CHIP and autoinflammation separately; once again, no significant differences were found. All the above comparisons analyses were repeated for ASC (only) specks, which produced similar results (Supplementary Figure 4). Cytokines IL‐1β, IL‐6, and IL‐18 levels were unaffected with increasing variant count (Figure 6B–D). We also compared type I IFN scores from AOSD cases with and without rare variants in the type I interferonopathy gene panel and found no significant IFN score increase in the mutated subset (Figure 6E).

## DISCUSSION

Despite significant progress in the field of AIDs, the pathogenesis of AOSD remains largely undefined. This is a rare disease, with clinical features overlapping with many other conditions, so it can be challenging to study. Nevertheless, there are important clues to its etiology. It is clinically similar to several mAIDs and responds to biologic therapies that target prototypic cytokines (IL‐1 and IL‐6) associated with innate immune system driven inflammation. These observations underpin our focus on genetic variants implicated in mAIDs and CHIP, both linked with heightened IL‐1 and IL‐6 levels.^11^, ^27^, ^28^, ^29^

Our analyses suggest a genetic correlation between AOSD and the presence of rare variants within genes associated with mAIDs and CHIP. Previous studies investigating genetic overlap between mAIDs in AOSD were limited to relatively few candidate genes, mainly those associated with hereditary fever syndromes with clinical features shared with AOSD such as TRAPS, FMF, CAPS, and mevalonate kinase deficiency.^4^ However, because some newly discovered mAIDs, such as A20 haploinsufficiency, have broad clinical manifestations, including features of sJIA and AOSD,^30^, ^31^ we expanded our analysis to incorporate most known mAIDs, which is currently more than 50 conditions. Furthermore, we analyzed the contribution of somatic mutations in the same genes, in recognition that such biologic mechanisms are responsible for pathogenesis of late‐onset AIDs. Overall, we found that a majority of AOSD cases (51.7%) carried multiple rare germline variants in different genes of interest. Fifteen variants were found to be significantly enriched within our AOSD cohort. Putative somatic variants were also identified in 30% of AOSD cases, adding further genetic complexity.

In the autoinflammatory panel, we identified rare variants previously linked with AOSD, such as *MEFV* c.184G>T, P.(G62W)^32^; however, the overall number of variants in this gene was far smaller than previously reported.^4^ We also identified several known pathogenic variants in other genes, with *TNFRSF1A* c.242G>T, P.(C81Y) being the most compelling. To date, all amino acid changes from the cysteine‐81 residue have been designated as pathogenic, confirming the critical importance of cysteine at this position (Infevers). Our patient with the P.(C81Y) substitution apparently had late‐onset disease presentation with symptoms at the age of 62 years. Although most patients with TRAPS develop symptoms in childhood, adult presentation has been reported.^19^ This is not the first time that an initial diagnosis of AOSD has later been attributed to TRAPS. A report of 20 patients with AOSD, which also identified one patient with the p.C81Y variant, suggests that prevalence of undiagnosed TRAPS might be up to 5% in unselected AOSD cohorts.^32^

Other genetic variants of note include *NLRC4* c.2357G>T, P.(G786V), which was identified in two separate AOSD cases. Although not obviously disease‐causing, this variant is enriched in cohorts of patients with periodic fever, aphthous stomatitis, pharyngitis, and adenitis^33^ (another polygenic AID), suggesting that certain variants might contribute to several related inflammatory diseases. When examining the occurrence of somatic variants in our autoinflammatory panel, we did not find any variants in *NLRP3* or *NLRC4*, the two genes for which low‐level somatic mutations have been previously described in late‐onset AIDs.^7^, ^8^, ^34^

Recent epidemiologic studies have linked CHIP with a broad range of chronic inflammatory diseases, including atherosclerosis, gout, and systemic sclerosis.^35^, ^36^, ^37^ To our knowledge, the prevalence of rare variants in CHIP‐associated genes in patients with AOSD has not been studied. We identified several putative somatic variants within our CHIP panel, present at a greater frequency in younger AOSD age groups than expected. This observation suggests that CHIP mutations may play a role in disease pathogenesis, irrespective of age at onset. Four somatic variants were identified in *DNMT3A* (P.(G534V), P.(P700A), P.(D529N), and P.(R320*)). DNMT3A is frequently mutated in CHIP and has the most evidence linking to systemic inflammation.^27^, ^29^ Unlike somatic variants, the effects of germline mutation in CHIP‐associated loci are unknown, but some investigations have reported germline influence in CHIP occurrence and progression.^38^, ^39^ We identified 13 rare germline variants in CHIP‐associated genes that were significantly enriched in our AOSD cohort, which suggests that these variants may genetically predispose individuals to disease. Further research is required to fully elucidate their impact.

With regards to germline variants, *FAT4* was the most frequently mutated gene within our preselected gene panel and is also associated with several cancers.^40^, ^41^, ^42^ *FAT4* encodes for FAT atypical cadherins 4, a membrane protein containing cadherin repeat sequences. *FAT4* mutations associated with malignancy are mainly attributed to somatic variants, whereas germline *FAT4* variants have only been associated with Hennekam syndrome and van Maldergem syndrome,^43^, ^44^ with their contributions to autoinflammatory phenotypes unknown.

A challenge we faced in our genetic analysis was the categorization of germline and somatic variants. The recently discovered importance of somatic variants in AIDs often means that retrospective patient cohorts lack matched “normal” specimens for somatic variant analysis. We tried to overcome this problem by running Mutect2 in tumor‐only mode against an in‐house panel of normal created from a HC cohort; this allowed us to filter common germline variants from the population. This method is widely used but does not achieve the same sensitivity on a per‐case basis with a matched normal sample. This was most problematic when somatic variants were identified with high variant allele fractions resembling a variant of germline origin. We attempted to use evidence from previous reports to raise putative somatic variants in cases of AOSD, but additional validation is required to confirm their association.

Transcriptomic analysis revealed dysregulation of gene expression in pathways associated with neutrophil activity and the innate immune system. Interestingly, elevated gene expression within these pathways was positively correlated with the SAS score, providing validity to its use as a potential biomarker for disease prognosis. Recently, whole blood transcriptomes from a similar data set were used to aid diagnosis in childhood febrile illness, which suggests both the utility and clinical applicability of this approach.^45^

We performed extensive inflammatory biomarker profiling of patients with AOSD and focused on the *NLRP3* inflammasome and linking genomic data with this profile. We reasoned that the cumulative proinflammatory effect of numerous rare variants might correlate with selected biomarkers, serving as surrogate functional validation of genomic findings. We used a novel assay to show that *NLRP3* inflammasome is highly activated in AOSD, particularly in patients with resistant and articular‐predominant disease. We also demonstrated that reduced levels of ASC/*NLRP3* specks correlates with treatment response in selected patients.

Previous studies demonstrated that serum ASC protein specks are a useful biomarker of inflammasome activation, in some circumstances predicting treatment response.^46^, ^47^, ^48^ However, these assays are unable to determine the origin of ASC specks, which is a significant limitation given that this adaptor protein is involved in activation of several inflammasome complexes. We have demonstrated the different distribution of ASC/*NLRP3* and ASC (only) specks in patients with AOSD, suggesting that additional non‐*NLRP3*‐inflammasomes may be relevant for disease pathogenesis. We produced similar findings when we used this assay to interrogate the role of *NLRP3* inflammasome in lower‐risk MDS.^49^

The interplay between inflammasomes and their varying roles in pathogenesis of mAIDs has been demonstrated previously.^50^ Different ASC‐containing inflammasomes have also been shown to have a role in the proinflammatory character of monocytes with certain CHIP‐related mutations. For example, murine monocytes with a truncating *ASXL1* mutation have increased AIM2 inflammasome activation and IL‐1β production, with inflammasome activation triggered by accumulated DNA damage.^51^ Our assay requires further refining to elucidate if there is contribution of different inflammasome complexes in AOSD pathogenesis or if the discrepancy between ASC (only) and ASC/*NLRP3* specks reflects the natural dissociation of the *NLRP3* component from the complexes.

We confirmed previous observations that IL‐18 is significantly elevated in AOSD compared with both HCs and other AIDs. IL‐18 levels might therefore be a useful diagnostic marker and contribute to a future validated and combined biochemical and clinical scoring system.^52^, ^53^, ^54^ The AOSD cohort had several other significantly elevated cytokines, most notably IL‐6 (confirming its role in AOSD pathogenesis) and IFN‐α2. Together with the observation that type I IFN scores were significantly elevated in patients with AOSD compared with HCs and disease controls, the raised IFN‐α2 levels suggest that the type I IFN pathway is relevant to disease pathogenesis. This justifies the use of, particularly in treatment‐resistant cases.

When we tested for correlation between various markers of inflammation and the burden of rare genetic variants in the autoinflammatory and/or CHIP gene panels, we found that the median level of ASC/*NLRP3* specks increased in patients with increasing variant count. However, this was not statistically significant, and overall, we were unable to find correlation with other inflammatory biomarkers tested. The lack of statistical significance may be due to limitations in sample size; nevertheless, this is of interest because ASC/*NLRP3* specks might be an important treatment target. Neutralization of the serum ASC/*NLRP3* specks using nanobodies has been shown to be an effective treatment strategy in several murine models of inflammatory diseases.^55^

The breadth of genes affected in this cohort demonstrates considerable genetic complexity within AOSD, likely influenced by the interplay of multiple genetic components. Many enriched variants were assessed by ClinVar to be of “uncertain significance” and “conflicting interpretations of pathogenicity,” suggesting they are individually insufficient for pathogenesis, supporting a multiple‐hit model. One way in which somatic mutations can collectively lead to clinical disease was demonstrated in a case of acquired NLRC4‐related AID, in which the concurrent *TET2* mutation allowed expansion of the hematopoietic stem cell clone carrying the *NLRC4* mutation, giving rise to the disease.^56^

Future multi‐omics approaches to AOSD research will help to untangle its complex pathogenesis. Although considered a distinct diagnosis, it is better seen as an umbrella term, covering overlapping pathotypes with distinct pathogenesis but common clinical manifestations. Recent work suggested patients might be subdivided into four subtypes, depending on clinical features and inflammatory biomarkers, each with different genetic backgrounds and pathologic mechanisms.^57^ This detailed clinical phenotyping, combined with future OMICs studies, will facilitate improved mapping of the biologic processes underpinning AOSD and identify additional treatment targets.

### AUTHOR CONTRIBUTIONS

All authors contributed to at least one of the following manuscript preparation roles: conceptualization AND/OR methodology, software, investigation, formal analysis, data curation, visualization, and validation AND drafting or reviewing/editing the final draft. As corresponding author, Dr Savic confirms that all authors have provided the final approval of the version to be published, and takes responsibility for the affirmations regarding article submission (eg, not under consideration by another journal), the integrity of the data presented, and the statements regarding compliance with institutional review board/Declaration of Helsinki requirements.

## Supporting information


**Disclosure Form**:

**Appendix S1.** Supporting Information

**Appendix S2.** Supporting Information

**Appendix S3.** Supporting Information

**Supplemental Figure S1. ASC/NLRP3 gating strategy**. Schematic diagram illustrating the methodology behind the ASC/NLRP3 Specks gating. The area highlighted in red reflects ASC‐PE and NLRP3‐APC double positive events.

**Supplemental Figure S2. Digital PCR (dPCR) validation of DNMT3A c.2512A>G**. A putative somatic variant *DNMT3A* c.2512A>G was identified in a healthy control at a read depth of 69× (WT 61 reads/ MU 8 reads) and we validated this variant to define our lower limit of somatic variant detection. **(A)** The mean copy number of wild‐type (*DNMT3A* c.2512A) and variant nucleotide (*DNMT3A* c.2512G) in each healthy control sample (n=15). The variant was detected in HC14 through exome sequencing and has also been detected through dPCR. **(B)** Variant allele fractions (%) of *DNMT3A* c.2512G detected in each sample. Each assay was run in duplicate for all individuals.

**Supplemental Figure S3.** Scatter plots comparing CRP (mg/L) against ASC specks (ASC/NLRP3: Red; ASC(only): Black; events/⎧L) for adult onset Still's disease (AOSD, n=106), Systemic juvenile idiopathic arthritis (SJIA, n=12), Schnitzler syndrome (SchS, n=10), Cryopyrin‐associated periodic syndrome (CAPS, n=11), familial Mediterranean fever (FMF, n=31), and the collective disease cohort. No statistically significant correlation was identified between CRP and ASC specks in the combined cohort nor individual disease.

**Supplemental Figure S4. (A)** ASC(only) specks in healthy controls (HC, n= 30) were compared to AOSD cohort subsets (AOSD#1 (n=39), AOSD#2 (n=30) and AOSD#3 (n=34)), SIJA (n=12), SchS (n=10), CAPS (n=11) and FMF (n=31) cohorts. The weighted bars represent median values. Statistically significant pairwise comparisons are annotated with * =*p*<0.05; ** =*p*<0.01; *** =*p*<0.001; **** =*p*<0.0001. Bar charts (median, IQR) illustrating ASC(only) specks between AOSD cases carrying 0, 1, 2, and 3+ variants (germline and/or somatic) within **(B)** any gene from our pre‐selected panels; **(C)** CHIP‐associated panel only and **(D)** autoinflammation panel only.

**Supplemental Figure S5. Chemokine concentrations for Type‐I Interferon scoring**. The Type‐I Interferon score was investigated using a custom Luminex Discovery Assay assessing chemokines: CXCL10, CCL2, CCL8, CCL19 and CXCL11. The raw data (pg/mL) from the HC (black) and AOSD (red) cohorts are displayed on stacked barcharts presenting median (IQR). Pairwise comparisons were assessed by Mann Whitney tests and statistical significance is denoted by asterisks. * =*p*<0.05; ** =*p*<0.01; *** =*p*<0.001; **** =*p*<0.0001.

**Supplemental Figure S6.** Comparisons between ASC/NLRP3 and ASC(only) specks against **(A)** CRP (mg/L) and **(B;C)** SAS in the treatment refractory AOSD cohort (AOSD#3, n=30). No significant correlations were identified between these variables.

**Supplemental Table S1.** Cohort demographics and treatment history.

**Supplemental Table S2.** Pre‐selected gene panels.

**Supplemental Table S3.** Whole exome sequencing metrics.

**Supplemental Table S4. Germline variants identified in cases of adult‐onset Still's disease**. An overview of 93 rare (<1% population allele frequency), potentially pathogenic (CADD>20) germline variants were identified in AOSD cases across targeted gene panels associated with CHIP, autoinflammation and type‐I interferonopathy. Genomic coordinates provided for the human reference genome build GRCh38. c.DNA nomenclature provided according to the selected RefSeq transcript. Enrichment analysis was performed for variants found unique to AOSD cases (89/93 variants), the incidence of each variant was compared to their prevalence recorded in the European (Non‐Finnish) population within gnomAD v4.0.0, and statistically significant enrichment is underlined in red (defined by adjusted p‐value <0.000352 after multiple testing correction). The pathogenicity classification from ClinVar and INFEVERS (autoinflammation panel only) are displayed for each variant to aid interpretation.

**Supplemental Table S5. Putative somatic variants identified in cases of adult‐onset Still's disease**. Putative somatic variants identified by Mutect2 are colour coded based on the gene panel category: CHIP (orange), Autoinflammatory (green), Type I interferonopathy (blue). Genomic coordinates are provided for the human reference genome build GRCh38. c.DNA nomenclature provided according to the assigned RefSeq transcript. Variants identified by Mutect2 alone, and not HaplotypeCaller, were flagged as putative somatic variants. The interpretation of the remaining variants was based on pre‐existing evidence in COSMIC. Mutations identified by both Mutect2 and HaplotypeCaller, with prior evidence as a somatic mutation were also flagged as putative somatic variants. All other variants were labelled as variants of “Uncertain” origin. VAF % values underlined denote a high value from an X chromosome gene in a male subject. The VAF % and read counts of variants carried by multiple AOSD cases are highlighted ^#^ and ^+^.

**Supplemental Table S6. Candidate variants from transcriptomics data**. 87 rare (<1% population allele frequency), potentially pathogenic (CADD>20) germline variants were identified across 61/170 genes, shared between the top three pathways enriched in the transcriptome data. The incidence of each variant was compared to their prevalence recorded in the European (Non‐Finnish) population within gnomAD v4.0.0, and statistically significant enrichment is underlined in red (defined by adjusted p‐value <0.0004 after multiple testing correction). Genomic coordinates are provided for the human reference genome build GRCh38. c.DNA nomenclature provided according to the selected RefSeq transcript. Cases carrying homozygous variants are highlighted ^B^.
