# Supplementary material for: Characterization of Genetic Landscape and Novel Inflammatory Biomarkers in Patients With Adult‐Onset Still's Disease
Source: Arthritis Rheumatol. 2024 Dec 16;77(5):582–95. doi: 10.1002/art.43054 (PMC12039473; doi:10.1002/art.43054)
Supplement: Supplementary file 1 — Disclosure Form: [file ART-77-582-s011.pdf]

## ICMJE DISCLOSURE FORM

**Date:______16/04/2024_______________________________________________________________________**
**Your Name:____Alice Ibbotson__________________________________________________________________**
**Manuscript Title:** Characterization of genetic landscape and novel inflammatory biomarkers in patients with adult-onset Still's disease
**Manuscript number (if known):_____** ar-23-1618.R1____________________________________________

**In the interest of transparency, we ask you to disclose all relationships/activities/interests listed below that are related to the content of your manuscript. "Related" means any relation with for-profit or not-for-profit third parties whose interests may be affected by the content of the manuscript. Disclosure represents a commitment to transparency and does not necessarily indicate a bias. If you are in doubt about whether to list a relationship/activity/interest, it is preferable that you do so.**

**The following questions apply to the author's relationships/activities/interests as they relate to the <u>current manuscript only</u>.**

**The author's relationships/activities/interests should be <u>defined broadly</u>. For example, if your manuscript pertains to the epidemiology of hypertension, you should declare all relationships with manufacturers of antihypertensive medication, even if that medication is not mentioned in the manuscript.**

**In item #1 below, report all support for the work reported in this manuscript without time limit. For all other items, the time frame for disclosure is the past 36 months.**

| | | **Name all entities with whom you have this relationship or indicate none (add rows as needed)** | **Specifications/Comments (e.g., if payments were made to you or to your institution)** |
|---|---|---|---|
| | | **Time frame: Since the initial planning of the work** | |
| 1 | All support for the present manuscript (e.g., funding, provision of study materials, medical writing, article processing charges, etc.) **No time limit for this item.** | __x__None | |
| | | | |
| | | | |
| | | | |
| | | | |
| | | | |
| | | | |
| | | | |
| | | **Time frame: past 36 months** | |
| 2 | Grants or contracts from any entity (if not indicated in item #1 above). | __x__None | |
| | | | |
| | | | |
| 3 | Royalties or licenses | __x__None | |
| | | | |
| | | | |
| 4 | Consulting fees | __x__None | |
| | | | |
| | | | |

| | | | |
|---|---|---|---|
| 5 | Payment or honoraria for lectures, presentations, speakers bureaus, manuscript writing or educational events | __x__None | |
| | | | |
| | | | |
| 6 | Payment for expert testimony | __x__None | |
| | | | |
| | | | |
| 7 | Support for attending meetings and/or travel | __x__None | |
| | | | |
| | | | |
| 8 | Patents planned, issued or pending | __x__None | |
| | | | |
| | | | |
| 9 | Participation on a Data Safety Monitoring Board or Advisory Board | __x__None | |
| | | | |
| | | | |
| 10 | Leadership or fiduciary role in other board, society, committee or advocacy group, paid or unpaid | __x__None | |
| | | | |
| | | | |
| 11 | Stock or stock options | __x__None | |
| | | | |
| | | | |
| 12 | Receipt of equipment, materials, drugs, medical writing, gifts or other services | __x__None | |
| | | | |
| | | | |
| 13 | Other financial or non-financial interests | __x__None | |
| | | | |
| | | | |

**Please place an "X" next to the following statement to indicate your agreement:**

**X I certify that I have answered every question and have not altered the wording of any of the questions on this form.**

## ICMJE DISCLOSURE FORM

**Date:______16/04/2024_______________________________________________________________________**
**Your Name:____Bruno Fautrel _____________________________________________________________________**
**Manuscript Title:** Characterization of genetic landscape and novel inflammatory biomarkers in patients with adult-onset Still's disease
**Manuscript number (if known):_____** ar-23-1618.R1______________________________________________

**In the interest of transparency, we ask you to disclose all relationships/activities/interests listed below that are related to the content of your manuscript. "Related" means any relation with for-profit or not-for-profit third parties whose interests may be affected by the content of the manuscript. Disclosure represents a commitment to transparency and does not necessarily indicate a bias. If you are in doubt about whether to list a relationship/activity/interest, it is preferable that you do so.**

**The following questions apply to the author's relationships/activities/interests as they relate to the current manuscript only.**

**The author's relationships/activities/interests should be defined broadly. For example, if your manuscript pertains to the epidemiology of hypertension, you should declare all relationships with manufacturers of antihypertensive medication, even if that medication is not mentioned in the manuscript.**

**In item #1 below, report all support for the work reported in this manuscript without time limit. For all other items, the time frame for disclosure is the past 36 months.**

| | | **Name all entities with whom you have this relationship or indicate none (add rows as needed)** | **Specifications/Comments (e.g., if payments were made to you or to your institution)** |
|---|---|---|---|
| | | **Time frame: Since the initial planning of the work** | |
| 1 | All support for the present manuscript (e.g., funding, provision of study materials, medical writing, article processing charges, etc.) **No time limit for this item.** | __x__None | |
| | | | |
| | | **Time frame: past 36 months** | |
| 2 | Grants or contracts from any entity (if not indicated in item #1 above). | __x__None | |
| 3 | Royalties or licenses | __x__None | |
| 4 | Consulting fees | Novartis Pharma | |
| | | Swedish Orphan Biovitrium | |
| | | | |

| | | | |
|---|---|---|---|
| 5 | Payment or honoraria for lectures, presentations, speakers bureaus, manuscript writing or educational events | __x__None | |
| | | | |
| | | | |
| 6 | Payment for expert testimony | __x__None | |
| | | | |
| | | | |
| 7 | Support for attending meetings and/or travel | Novartis | |
| | | | |
| | | | |
| 8 | Patents planned, issued or pending | __x__None | |
| | | | |
| | | | |
| 9 | Participation on a Data Safety Monitoring Board or Advisory Board | __x__None | |
| | | | |
| | | | |
| 10 | Leadership or fiduciary role in other board, society, committee or advocacy group, paid or unpaid | __x__None | |
| | | | |
| | | | |
| 11 | Stock or stock options | __x__None | |
| | | | |
| | | | |
| 12 | Receipt of equipment, materials, drugs, medical writing, gifts or other services | __x__None | |
| | | | |
| | | | |
| 13 | Other financial or non-financial interests | __x__None | |
| | | | |
| | | | |

**Please place an "X" next to the following statement to indicate your agreement:**

**X I certify that I have answered every question and have not altered the wording of any of the questions on this form.**

## ICMJE DISCLOSURE FORM

**Date:______16/04/2024________________________________________________________________________**
**Your Name:____Catherine Cargo ____________________________________________________________________**
**Manuscript Title:** Characterization of genetic landscape and novel inflammatory biomarkers in patients with adult-onset Still's disease
**Manuscript number (if known):_____** ar-23-1618.R1__________________________________________

**In the interest of transparency, we ask you to disclose all relationships/activities/interests listed below that are related to the content of your manuscript. "Related" means any relation with for-profit or not-for-profit third parties whose interests may be affected by the content of the manuscript. Disclosure represents a commitment to transparency and does not necessarily indicate a bias. If you are in doubt about whether to list a relationship/activity/interest, it is preferable that you do so.**

**The following questions apply to the author's relationships/activities/interests as they relate to the current manuscript only.**

**The author's relationships/activities/interests should be defined broadly. For example, if your manuscript pertains to the epidemiology of hypertension, you should declare all relationships with manufacturers of antihypertensive medication, even if that medication is not mentioned in the manuscript.**

**In item #1 below, report all support for the work reported in this manuscript without time limit. For all other items, the time frame for disclosure is the past 36 months.**

| | | **Name all entities with whom you have this relationship or indicate none (add rows as needed)** | **Specifications/Comments (e.g., if payments were made to you or to your institution)** |
|---|---|---|---|
| **Time frame: Since the initial planning of the work** | | | |
| 1 | All support for the present manuscript (e.g., funding, provision of study materials, medical writing, article processing charges, etc.) **No time limit for this item.** | __x__None | |
| | | | |
| | | | |
| | | | |
| | | | |
| | | | |
| | | | |
| | | | |
| **Time frame: past 36 months** | | | |
| 2 | Grants or contracts from any entity (if not indicated in item #1 above). | __x__None | |
| | | | |
| | | | |
| 3 | Royalties or licenses | __x__None | |
| | | | |
| | | | |
| 4 | Consulting fees | __x__None | |
| | | | |
| | | | |

| | | | |
|---|---|---|---|
| 5 | Payment or honoraria for lectures, presentations, speakers bureaus, manuscript writing or educational events | __x__None | |
| | | | |
| | | | |
| 6 | Payment for expert testimony | __x__None | |
| | | | |
| | | | |
| 7 | Support for attending meetings and/or travel | __x__None | |
| | | | |
| | | | |
| 8 | Patents planned, issued or pending | __x__None | |
| | | | |
| | | | |
| 9 | Participation on a Data Safety Monitoring Board or Advisory Board | __x__None | |
| | | | |
| | | | |
| 10 | Leadership or fiduciary role in other board, society, committee or advocacy group, paid or unpaid | __x__None | |
| | | | |
| | | | |
| 11 | Stock or stock options | __x__None | |
| | | | |
| | | | |
| 12 | Receipt of equipment, materials, drugs, medical writing, gifts or other services | __x__None | |
| | | | |
| | | | |
| 13 | Other financial or non-financial interests | __x__None | |
| | | | |
| | | | |

**Please place an "X" next to the following statement to indicate your agreement:**

**X I certify that I have answered every question and have not altered the wording of any of the questions on this form.**

# ICMJE DISCLOSURE FORM

**Date:______16/04/2024________________________________________________________________________**
**Your Name:____Clive Carter______________________________________________________________________**
**Manuscript Title:** Characterization of genetic landscape and novel inflammatory biomarkers in patients with adult-onset Still's disease
**Manuscript number (if known):_____** ar-23-1618.R1___________________________________________

**In the interest of transparency, we ask you to disclose all relationships/activities/interests listed below that are related to the content of your manuscript. "Related" means any relation with for-profit or not-for-profit third parties whose interests may be affected by the content of the manuscript. Disclosure represents a commitment to transparency and does not necessarily indicate a bias. If you are in doubt about whether to list a relationship/activity/interest, it is preferable that you do so.**

**The following questions apply to the author's relationships/activities/interests as they relate to the current manuscript only.**

**The author's relationships/activities/interests should be defined broadly. For example, if your manuscript pertains to the epidemiology of hypertension, you should declare all relationships with manufacturers of antihypertensive medication, even if that medication is not mentioned in the manuscript.**

**In item #1 below, report all support for the work reported in this manuscript without time limit. For all other items, the time frame for disclosure is the past 36 months.**

| | | **Name all entities with whom you have this relationship or indicate none (add rows as needed)** | **Specifications/Comments (e.g., if payments were made to you or to your institution)** |
|---|---|---|---|
| | | **Time frame: Since the initial planning of the work** | |
| 1 | All support for the present manuscript (e.g., funding, provision of study materials, medical writing, article processing charges, etc.) **No time limit for this item.** | __x__None | |
| | | | |
| | | | |
| | | | |
| | | | |
| | | | |
| | | | |
| | | | |
| | | **Time frame: past 36 months** | |
| 2 | Grants or contracts from any entity (if not indicated in item #1 above). | __x__None | |
| | | | |
| | | | |
| 3 | Royalties or licenses | __x__None | |
| | | | |
| | | | |
| 4 | Consulting fees | __x__None | |
| | | | |
| | | | |

| | | | |
|---|---|---|---|
| 5 | Payment or honoraria for lectures, presentations, speakers bureaus, manuscript writing or educational events | __x__None | |
| | | | |
| | | | |
| 6 | Payment for expert testimony | __x__None | |
| | | | |
| | | | |
| 7 | Support for attending meetings and/or travel | __x__None | |
| | | | |
| | | | |
| 8 | Patents planned, issued or pending | __x__None | |
| | | | |
| | | | |
| 9 | Participation on a Data Safety Monitoring Board or Advisory Board | __x__None | |
| | | | |
| | | | |
| 10 | Leadership or fiduciary role in other board, society, committee or advocacy group, paid or unpaid | __x__None | |
| | | | |
| | | | |
| 11 | Stock or stock options | __x__None | |
| | | | |
| | | | |
| 12 | Receipt of equipment, materials, drugs, medical writing, gifts or other services | __x__None | |
| | | | |
| | | | |
| 13 | Other financial or non-financial interests | __x__None | |
| | | | |
| | | | |

**Please place an "X" next to the following statement to indicate your agreement:**

**X I certify that I have answered every question and have not altered the wording of any of the questions on this form.**

**ICMJE DISCLOSURE FORM**

**Date:______16/04/2024___________________________________________________________________**
**Your Name:____Christopher Watson___________________________________________________________**
**Manuscript Title:** Characterization of genetic landscape and novel inflammatory biomarkers in patients with adult-onset Still's disease
**Manuscript number (if known):_____** ar-23-1618.R1__________________________________________

**In the interest of transparency, we ask you to disclose all relationships/activities/interests listed below that are related to the content of your manuscript. "Related" means any relation with for-profit or not-for-profit third parties whose interests may be affected by the content of the manuscript. Disclosure represents a commitment to transparency and does not necessarily indicate a bias. If you are in doubt about whether to list a relationship/activity/interest, it is preferable that you do so.**

**The following questions apply to the author's relationships/activities/interests as they relate to the current manuscript only.**

**The author's relationships/activities/interests should be defined broadly. For example, if your manuscript pertains to the epidemiology of hypertension, you should declare all relationships with manufacturers of antihypertensive medication, even if that medication is not mentioned in the manuscript.**

**In item #1 below, report all support for the work reported in this manuscript without time limit. For all other items, the time frame for disclosure is the past 36 months.**

| | | **Name all entities with whom you have this relationship or indicate none (add rows as needed)** | **Specifications/Comments (e.g., if payments were made to you or to your institution)** |
|---|---|---|---|
| | | **Time frame: Since the initial planning of the work** | |
| 1 | All support for the present manuscript (e.g., funding, provision of study materials, medical writing, article processing charges, etc.) **No time limit for this item.** | __x__None | |
| | | | |
| | | | |
| | | | |
| | | | |
| | | | |
| | | | |
| | | | |
| | | **Time frame: past 36 months** | |
| 2 | Grants or contracts from any entity (if not indicated in item #1 above). | __x__None | |
| | | | |
| | | | |
| 3 | Royalties or licenses | __x__None | |
| | | | |
| | | | |
| 4 | Consulting fees | __x__None | |
| | | | |
| | | | |

| | | | |
|---|---|---|---|
| 5 | Payment or honoraria for lectures, presentations, speakers bureaus, manuscript writing or educational events | __x__None | |
| | | | |
| | | | |
| 6 | Payment for expert testimony | __x__None | |
| | | | |
| | | | |
| 7 | Support for attending meetings and/or travel | __x__None | |
| | | | |
| | | | |
| 8 | Patents planned, issued or pending | __x__None | |
| | | | |
| | | | |
| 9 | Participation on a Data Safety Monitoring Board or Advisory Board | __x__None | |
| | | | |
| | | | |
| 10 | Leadership or fiduciary role in other board, society, committee or advocacy group, paid or unpaid | __x__None | |
| | | | |
| | | | |
| 11 | Stock or stock options | __x__None | |
| | | | |
| | | | |
| 12 | Receipt of equipment, materials, drugs, medical writing, gifts or other services | __x__None | |
| | | | |
| | | | |
| 13 | Other financial or non-financial interests | __x__None | |
| | | | |
| | | | |

**Please place an "X" next to the following statement to indicate your agreement:**

**X I certify that I have answered every question and have not altered the wording of any of the questions on this form.**

# ICMJE DISCLOSURE FORM

**Date:______16/04/2024____________________________________________________________________**
**Your Name:____Eugen Feist ________________________________________________________________**
**Manuscript Title:** Characterization of genetic landscape and novel inflammatory biomarkers in patients with adult-onset Still's disease
**Manuscript number (if known):_____** ar-23-1618.R1__________________________________________

**In the interest of transparency, we ask you to disclose all relationships/activities/interests listed below that are related to the content of your manuscript. "Related" means any relation with for-profit or not-for-profit third parties whose interests may be affected by the content of the manuscript. Disclosure represents a commitment to transparency and does not necessarily indicate a bias. If you are in doubt about whether to list a relationship/activity/interest, it is preferable that you do so.**

**The following questions apply to the author's relationships/activities/interests as they relate to the <u>current manuscript only</u>.**

**The author's relationships/activities/interests should be <u>defined broadly</u>. For example, if your manuscript pertains to the epidemiology of hypertension, you should declare all relationships with manufacturers of antihypertensive medication, even if that medication is not mentioned in the manuscript.**

**In item #1 below, report all support for the work reported in this manuscript without time limit. For all other items, the time frame for disclosure is the past 36 months.**

| | | **Name all entities with whom you have this relationship or indicate none (add rows as needed)** | **Specifications/Comments (e.g., if payments were made to you or to your institution)** |
|---|---|---|---|
| **Time frame: Since the initial planning of the work** | | | |
| 1 | All support for the present manuscript (e.g., funding, provision of study materials, medical writing, article processing charges, etc.) **No time limit for this item.** | __x__None | |
| | | | |
| | | | |
| | | | |
| | | | |
| | | | |
| | | | |
| | | | |
| **Time frame: past 36 months** | | | |
| 2 | Grants or contracts from any entity (if not indicated in item #1 above). | __x__None | |
| | | | |
| | | | |
| 3 | Royalties or licenses | __x__None | |
| | | | |
| | | | |
| 4 | Consulting fees | Novartis Pharma | Personal payment |
| | | Swedish Orphan Biovitrium | Personal payment |
| | | | |

| | | | |
|---|---|---|---|
| 5 | Payment or honoraria for lectures, presentations, speakers bureaus, manuscript writing or educational events | Novartis, SOBI | Personal payment |
| | | | |
| | | | |
| 6 | Payment for expert testimony | __x__None | |
| | | | |
| | | | |
| 7 | Support for attending meetings and/or travel | Novartis | Personal payment |
| | | | |
| | | | |
| 8 | Patents planned, issued or pending | __x__None | |
| | | | |
| | | | |
| 9 | Participation on a Data Safety Monitoring Board or Advisory Board | Novartis | Personal payment |
| | | | |
| | | | |
| 10 | Leadership or fiduciary role in other board, society, committee or advocacy group, paid or unpaid | __x__None | |
| | | | |
| | | | |
| 11 | Stock or stock options | __x__None | |
| | | | |
| | | | |
| 12 | Receipt of equipment, materials, drugs, medical writing, gifts or other services | __x__None | |
| | | | |
| | | | |
| 13 | Other financial or non-financial interests | __x__None | |
| | | | |
| | | | |

**Please place an "X" next to the following statement to indicate your agreement:**

**X I certify that I have answered every question and have not altered the wording of any of the questions on this form.**

## ICMJE DISCLOSURE FORM

**Date:______16/04/2024______________________________________________________________________**
**Your Name:____Franceso DelGaldo ______________________________________________________________**
**Manuscript Title:** Characterization of genetic landscape and novel inflammatory biomarkers in patients with adult-onset Still's disease
**Manuscript number (if known):_____** ar-23-1618.R1____________________________________________

**In the interest of transparency, we ask you to disclose all relationships/activities/interests listed below that are related to the content of your manuscript. "Related" means any relation with for-profit or not-for-profit third parties whose interests may be affected by the content of the manuscript. Disclosure represents a commitment to transparency and does not necessarily indicate a bias. If you are in doubt about whether to list a relationship/activity/interest, it is preferable that you do so.**

**The following questions apply to the author's relationships/activities/interests as they relate to the current manuscript only.**

**The author's relationships/activities/interests should be defined broadly. For example, if your manuscript pertains to the epidemiology of hypertension, you should declare all relationships with manufacturers of antihypertensive medication, even if that medication is not mentioned in the manuscript.**

**In item #1 below, report all support for the work reported in this manuscript without time limit. For all other items, the time frame for disclosure is the past 36 months.**

| | | **Name all entities with whom you have this relationship or indicate none (add rows as needed)** | **Specifications/Comments (e.g., if payments were made to you or to your institution)** |
|---|---|---|---|
| | | **Time frame: Since the initial planning of the work** | |
| 1 | All support for the present manuscript (e.g., funding, provision of study materials, medical writing, article processing charges, etc.) **No time limit for this item.** | __x__None | |
| | | | |
| | | | |
| | | | |
| | | | |
| | | | |
| | | | |
| | | | |
| | | **Time frame: past 36 months** | |
| 2 | Grants or contracts from any entity (if not indicated in item #1 above). | __x__None | |
| | | | |
| | | | |
| 3 | Royalties or licenses | __x__None | |
| | | | |
| | | | |
| 4 | Consulting fees | __x__None | |
| | | | |
| | | | |

| | | | |
|---|---|---|---|
| 5 | Payment or honoraria for lectures, presentations, speakers bureaus, manuscript writing or educational events | __x__None | |
| | | | |
| | | | |
| 6 | Payment for expert testimony | __x__None | |
| | | | |
| | | | |
| 7 | Support for attending meetings and/or travel | __x__None | |
| | | | |
| | | | |
| 8 | Patents planned, issued or pending | __x__None | |
| | | | |
| | | | |
| 9 | Participation on a Data Safety Monitoring Board or Advisory Board | __x__None | |
| | | | |
| | | | |
| 10 | Leadership or fiduciary role in other board, society, committee or advocacy group, paid or unpaid | __x__None | |
| | | | |
| | | | |
| 11 | Stock or stock options | __x__None | |
| | | | |
| | | | |
| 12 | Receipt of equipment, materials, drugs, medical writing, gifts or other services | __x__None | |
| | | | |
| | | | |
| 13 | Other financial or non-financial interests | __x__None | |
| | | | |
| | | | |

**Please place an "X" next to the following statement to indicate your agreement:**

**X I certify that I have answered every question and have not altered the wording of any of the questions on this form.**

## ICMJE DISCLOSURE FORM

**Date:______16/04/2024________________________________________________________________________**
**Your Name:____Fatima Nadat_____________________________________________________________________**
**Manuscript Title:** Characterization of genetic landscape and novel inflammatory biomarkers in patients with adult-onset Still's disease
**Manuscript number (if known):_____** ar-23-1618.R1**__________________________________________**

**In the interest of transparency, we ask you to disclose all relationships/activities/interests listed below that are related to the content of your manuscript. "Related" means any relation with for-profit or not-for-profit third parties whose interests may be affected by the content of the manuscript. Disclosure represents a commitment to transparency and does not necessarily indicate a bias. If you are in doubt about whether to list a relationship/activity/interest, it is preferable that you do so.**

**The following questions apply to the author's relationships/activities/interests as they relate to the current manuscript only.**

**The author's relationships/activities/interests should be defined broadly. For example, if your manuscript pertains to the epidemiology of hypertension, you should declare all relationships with manufacturers of antihypertensive medication, even if that medication is not mentioned in the manuscript.**

**In item #1 below, report all support for the work reported in this manuscript without time limit. For all other items, the time frame for disclosure is the past 36 months.**

| | | **Name all entities with whom you have this relationship or indicate none (add rows as needed)** | **Specifications/Comments (e.g., if payments were made to you or to your institution)** |
|---|---|---|---|
| | | **Time frame: Since the initial planning of the work** | |
| 1 | All support for the present manuscript (e.g., funding, provision of study materials, medical writing, article processing charges, etc.) **No time limit for this item.** | __x__None | |
| | | | |
| | | | |
| | | | |
| | | | |
| | | | |
| | | | |
| | | | |
| | | **Time frame: past 36 months** | |
| 2 | Grants or contracts from any entity (if not indicated in item #1 above). | __x__None | |
| | | | |
| | | | |
| 3 | Royalties or licenses | __x__None | |
| | | | |
| | | | |
| 4 | Consulting fees | __x__None | |
| | | | |
| | | | |

| | | | |
|---|---|---|---|
| 5 | Payment or honoraria for lectures, presentations, speakers bureaus, manuscript writing or educational events | __x__None | |
| | | | |
| | | | |
| 6 | Payment for expert testimony | __x__None | |
| | | | |
| | | | |
| 7 | Support for attending meetings and/or travel | __x__None | |
| | | | |
| | | | |
| 8 | Patents planned, issued or pending | __x__None | |
| | | | |
| | | | |
| 9 | Participation on a Data Safety Monitoring Board or Advisory Board | __x__None | |
| | | | |
| | | | |
| 10 | Leadership or fiduciary role in other board, society, committee or advocacy group, paid or unpaid | __x__None | |
| | | | |
| | | | |
| 11 | Stock or stock options | __x__None | |
| | | | |
| | | | |
| 12 | Receipt of equipment, materials, drugs, medical writing, gifts or other services | __x__None | |
| | | | |
| | | | |
| 13 | Other financial or non-financial interests | __x__None | |
| | | | |
| | | | |

**Please place an "X" next to the following statement to indicate your agreement:**

**X I certify that I have answered every question and have not altered the wording of any of the questions on this form.**

## ICMJE DISCLOSURE FORM

**Date:______16/04/2024______________________________________________________________________**
**Your Name:____James A Poulter_____________________________________________________________________**
**Manuscript Title:** Characterization of genetic landscape and novel inflammatory biomarkers in patients with adult-onset Still's disease
**Manuscript number (if known):_____** ar-23-1618.R1_____________________________________________

**In the interest of transparency, we ask you to disclose all relationships/activities/interests listed below that are related to the content of your manuscript. "Related" means any relation with for-profit or not-for-profit third parties whose interests may be affected by the content of the manuscript. Disclosure represents a commitment to transparency and does not necessarily indicate a bias. If you are in doubt about whether to list a relationship/activity/interest, it is preferable that you do so.**

**The following questions apply to the author's relationships/activities/interests as they relate to the current manuscript only.**

**The author's relationships/activities/interests should be defined broadly. For example, if your manuscript pertains to the epidemiology of hypertension, you should declare all relationships with manufacturers of antihypertensive medication, even if that medication is not mentioned in the manuscript.**

**In item #1 below, report all support for the work reported in this manuscript without time limit. For all other items, the time frame for disclosure is the past 36 months.**

| | | **Name all entities with whom you have this relationship or indicate none (add rows as needed)** | **Specifications/Comments (e.g., if payments were made to you or to your institution)** |
|---|---|---|---|
| **Time frame: Since the initial planning of the work** | | | |
| 1 | All support for the present manuscript (e.g., funding, provision of study materials, medical writing, article processing charges, etc.) **No time limit for this item.** | __x__None | |
| | | | |
| | | | |
| | | | |
| | | | |
| | | | |
| | | | |
| | | | |
| **Time frame: past 36 months** | | | |
| 2 | Grants or contracts from any entity (if not indicated in item #1 above). | __x__None | |
| | | | |
| | | | |
| 3 | Royalties or licenses | __x__None | |
| | | | |
| | | | |
| 4 | Consulting fees | __x__None | |
| | | | |
| | | | |

| | | | |
|---|---|---|---|
| 5 | Payment or honoraria for lectures, presentations, speakers bureaus, manuscript writing or educational events | __x__None | |
| | | | |
| | | | |
| 6 | Payment for expert testimony | __x__None | |
| | | | |
| | | | |
| 7 | Support for attending meetings and/or travel | __x__None | |
| | | | |
| | | | |
| 8 | Patents planned, issued or pending | __x__None | |
| | | | |
| | | | |
| 9 | Participation on a Data Safety Monitoring Board or Advisory Board | __x__None | |
| | | | |
| | | | |
| 10 | Leadership or fiduciary role in other board, society, committee or advocacy group, paid or unpaid | __x__None | |
| | | | |
| | | | |
| 11 | Stock or stock options | __x__None | |
| | | | |
| | | | |
| 12 | Receipt of equipment, materials, drugs, medical writing, gifts or other services | __x__None | |
| | | | |
| | | | |
| 13 | Other financial or non-financial interests | __x__None | |
| | | | |
| | | | |

**Please place an "X" next to the following statement to indicate your agreement:**

**X I certify that I have answered every question and have not altered the wording of any of the questions on this form.**

## ICMJE DISCLOSURE FORM

**Date:______16/04/2024____________________________________________________________________________**
**Your Name:____Jurgen Rech _________________________________________________________________________**
**Manuscript Title:** Characterization of genetic landscape and novel inflammatory biomarkers in patients with adult-onset Still's disease
**Manuscript number (if known):_____** ar-23-1618.R1______________________________________________

**In the interest of transparency, we ask you to disclose all relationships/activities/interests listed below that are related to the content of your manuscript. "Related" means any relation with for-profit or not-for-profit third parties whose interests may be affected by the content of the manuscript. Disclosure represents a commitment to transparency and does not necessarily indicate a bias. If you are in doubt about whether to list a relationship/activity/interest, it is preferable that you do so.**

**The following questions apply to the author's relationships/activities/interests as they relate to the current manuscript only.**

**The author's relationships/activities/interests should be defined broadly. For example, if your manuscript pertains to the epidemiology of hypertension, you should declare all relationships with manufacturers of antihypertensive medication, even if that medication is not mentioned in the manuscript.**

**In item #1 below, report all support for the work reported in this manuscript without time limit. For all other items, the time frame for disclosure is the past 36 months.**

| | | **Name all entities with whom you have this relationship or indicate none (add rows as needed)** | **Specifications/Comments (e.g., if payments were made to you or to your institution)** |
|---|---|---|---|
| | | **Time frame: Since the initial planning of the work** | |
| 1 | All support for the present manuscript (e.g., funding, provision of study materials, medical writing, article processing charges, etc.) **No time limit for this item.** | __x__None | |
| | | | |
| | | | |
| | | | |
| | | | |
| | | | |
| | | | |
| | | | |
| | | **Time frame: past 36 months** | |
| 2 | Grants or contracts from any entity (if not indicated in item #1 above). | Swedish Orphan | |
| | | Novartis Pharma Gmbh, | |
| | | Biovitrium GmbH | |
| 3 | Royalties or licenses | __x__None | |
| | | | |
| | | | |
| 4 | Consulting fees | Novartis Pharma Gmbh, | |
| | | Swedish Orphan Biovitrium | |
| | | | |

| | | | |
|---|---|---|---|
| 5 | Payment or honoraria for lectures, presentations, speakers bureaus, manuscript writing or educational events | Novartis Pharma Gmbh, | |
| | | Swedish Orphan Biovitrium | |
| | | | |
| 6 | Payment for expert testimony | __x__None | |
| | | | |
| | | | |
| 7 | Support for attending meetings and/or travel | __x__None | |
| | | | |
| | | | |
| 8 | Patents planned, issued or pending | __x__None | |
| | | | |
| | | | |
| 9 | Participation on a Data Safety Monitoring Board or Advisory Board | __x__None | |
| | | | |
| | | | |
| 10 | Leadership or fiduciary role in other board, society, committee or advocacy group, paid or unpaid | __x__None | |
| | | | |
| | | | |
| 11 | Stock or stock options | __x__None | |
| | | | |
| | | | |
| 12 | Receipt of equipment, materials, drugs, medical writing, gifts or other services | __x__None | |
| | | | |
| | | | |
| 13 | Other financial or non-financial interests | __x__None | |
| | | | |
| | | | |

**Please place an "X" next to the following statement to indicate your agreement:**

**X I certify that I have answered every question and have not altered the wording of any of the questions on this form.**

## ICMJE DISCLOSURE FORM

**Date:______16/04/2024__________________________________________________________**
**Your Name:____Joanne Topping__________________________________________________________**
**Manuscript Title:** Characterization of genetic landscape and novel inflammatory biomarkers in patients with adult-onset Still's disease
**Manuscript number (if known):_____** ar-23-1618.R1____________________________________________

**In the interest of transparency, we ask you to disclose all relationships/activities/interests listed below that are related to the content of your manuscript. "Related" means any relation with for-profit or not-for-profit third parties whose interests may be affected by the content of the manuscript. Disclosure represents a commitment to transparency and does not necessarily indicate a bias. If you are in doubt about whether to list a relationship/activity/interest, it is preferable that you do so.**

**The following questions apply to the author's relationships/activities/interests as they relate to the current manuscript only.**

**The author's relationships/activities/interests should be defined broadly. For example, if your manuscript pertains to the epidemiology of hypertension, you should declare all relationships with manufacturers of antihypertensive medication, even if that medication is not mentioned in the manuscript.**

**In item #1 below, report all support for the work reported in this manuscript without time limit. For all other items, the time frame for disclosure is the past 36 months.**

| | | **Name all entities with whom you have this relationship or indicate none (add rows as needed)** | **Specifications/Comments (e.g., if payments were made to you or to your institution)** |
|---|---|---|---|
| | | **Time frame: Since the initial planning of the work** | |
| 1 | All support for the present manuscript (e.g., funding, provision of study materials, medical writing, article processing charges, etc.) **No time limit for this item.** | __x__None | |
| | | | |
| | | | |
| | | | |
| | | | |
| | | | |
| | | | |
| | | | |
| | | **Time frame: past 36 months** | |
| 2 | Grants or contracts from any entity (if not indicated in item #1 above). | __x__None | |
| | | | |
| | | | |
| 3 | Royalties or licenses | __x__None | |
| | | | |
| | | | |
| 4 | Consulting fees | __x__None | |
| | | | |
| | | | |

| | | | |
|---|---|---|---|
| 5 | Payment or honoraria for lectures, presentations, speakers bureaus, manuscript writing or educational events | __x__None | |
| | | | |
| | | | |
| 6 | Payment for expert testimony | __x__None | |
| | | | |
| | | | |
| 7 | Support for attending meetings and/or travel | __x__None | |
| | | | |
| | | | |
| 8 | Patents planned, issued or pending | __x__None | |
| | | | |
| | | | |
| 9 | Participation on a Data Safety Monitoring Board or Advisory Board | __x__None | |
| | | | |
| | | | |
| 10 | Leadership or fiduciary role in other board, society, committee or advocacy group, paid or unpaid | __x__None | |
| | | | |
| | | | |
| 11 | Stock or stock options | __x__None | |
| | | | |
| | | | |
| 12 | Receipt of equipment, materials, drugs, medical writing, gifts or other services | __x__None | |
| | | | |
| | | | |
| 13 | Other financial or non-financial interests | __x__None | |
| | | | |
| | | | |

**Please place an "X" next to the following statement to indicate your agreement:**

**X I certify that I have answered every question and have not altered the wording of any of the questions on this form.**

## ICMJE DISCLOSURE FORM

**Date:______16/04/2024____________________________________________________________________**
**Your Name:____Jan Zernicke_____________________________________________________________**
**Manuscript Title:** Characterization of genetic landscape and novel inflammatory biomarkers in patients with adult-onset Still's disease
**Manuscript number (if known):_____** ar-23-1618.R1**___________________________________________**

**In the interest of transparency, we ask you to disclose all relationships/activities/interests listed below that are related to the content of your manuscript. "Related" means any relation with for-profit or not-for-profit third parties whose interests may be affected by the content of the manuscript. Disclosure represents a commitment to transparency and does not necessarily indicate a bias. If you are in doubt about whether to list a relationship/activity/interest, it is preferable that you do so.**

**The following questions apply to the author's relationships/activities/interests as they relate to the current manuscript only.**

**The author's relationships/activities/interests should be defined broadly. For example, if your manuscript pertains to the epidemiology of hypertension, you should declare all relationships with manufacturers of antihypertensive medication, even if that medication is not mentioned in the manuscript.**

**In item #1 below, report all support for the work reported in this manuscript without time limit. For all other items, the time frame for disclosure is the past 36 months.**

| | | **Name all entities with whom you have this relationship or indicate none (add rows as needed)** | **Specifications/Comments (e.g., if payments were made to you or to your institution)** |
|---|---|---|---|
| **Time frame: Since the initial planning of the work** | | | |
| 1 | All support for the present manuscript (e.g., funding, provision of study materials, medical writing, article processing charges, etc.) **No time limit for this item.** | __x__None | |
| | | | |
| | | | |
| | | | |
| | | | |
| | | | |
| | | | |
| | | | |
| **Time frame: past 36 months** | | | |
| 2 | Grants or contracts from any entity (if not indicated in item #1 above). | __x__None | |
| | | | |
| | | | |
| 3 | Royalties or licenses | __x__None | |
| | | | |
| | | | |
| 4 | Consulting fees | __x__None | |
| | | | |
| | | | |

| | | | |
|---|---|---|---|
| 5 | Payment or honoraria for lectures, presentations, speakers bureaus, manuscript writing or educational events | __x__None | |
| | | | |
| | | | |
| 6 | Payment for expert testimony | __x__None | |
| | | | |
| | | | |
| 7 | Support for attending meetings and/or travel | __x__None | |
| | | | |
| | | | |
| 8 | Patents planned, issued or pending | __x__None | |
| | | | |
| | | | |
| 9 | Participation on a Data Safety Monitoring Board or Advisory Board | __x__None | |
| | | | |
| | | | |
| 10 | Leadership or fiduciary role in other board, society, committee or advocacy group, paid or unpaid | __x__None | |
| | | | |
| | | | |
| 11 | Stock or stock options | __x__None | |
| | | | |
| | | | |
| 12 | Receipt of equipment, materials, drugs, medical writing, gifts or other services | __x__None | |
| | | | |
| | | | |
| 13 | Other financial or non-financial interests | __x__None | |
| | | | |
| | | | |

**Please place an "X" next to the following statement to indicate your agreement:**

**X I certify that I have answered every question and have not altered the wording of any of the questions on this form.**

**ICMJE DISCLOSURE FORM**

**Date:______16/04/2024_____________________________________________________________________**
**Your Name:____Kenneth G C Smith ____________________________________________________________**
**Manuscript Title:** Characterization of genetic landscape and novel inflammatory biomarkers in patients with adult-onset Still's disease
**Manuscript number (if known):_____** ar-23-1618.R1**_____________________________________________**

**In the interest of transparency, we ask you to disclose all relationships/activities/interests listed below that are related to the content of your manuscript. "Related" means any relation with for-profit or not-for-profit third parties whose interests may be affected by the content of the manuscript. Disclosure represents a commitment to transparency and does not necessarily indicate a bias. If you are in doubt about whether to list a relationship/activity/interest, it is preferable that you do so.**

**The following questions apply to the author's relationships/activities/interests as they relate to the current manuscript only.**

**The author's relationships/activities/interests should be defined broadly. For example, if your manuscript pertains to the epidemiology of hypertension, you should declare all relationships with manufacturers of antihypertensive medication, even if that medication is not mentioned in the manuscript.**

**In item #1 below, report all support for the work reported in this manuscript without time limit. For all other items, the time frame for disclosure is the past 36 months.**

| | | **Name all entities with whom you have this relationship or indicate none (add rows as needed)** | **Specifications/Comments (e.g., if payments were made to you or to your institution)** |
|---|---|---|---|
| | | **Time frame: Since the initial planning of the work** | |
| 1 | All support for the present manuscript (e.g., funding, provision of study materials, medical writing, article processing charges, etc.) **No time limit for this item.** | __x__None | |
| | | | |
| | | | |
| | | | |
| | | | |
| | | | |
| | | | |
| | | | |
| | | **Time frame: past 36 months** | |
| 2 | Grants or contracts from any entity (if not indicated in item #1 above). | __x__None | |
| | | | |
| | | | |
| 3 | Royalties or licenses | __x__None | |
| | | | |
| | | | |
| 4 | Consulting fees | __x__None | |
| | | | |
| | | | |

| | | | |
|---|---|---|---|
| 5 | Payment or honoraria for lectures, presentations, speakers bureaus, manuscript writing or educational events | __x__None | |
| | | | |
| | | | |
| 6 | Payment for expert testimony | __x__None | |
| | | | |
| | | | |
| 7 | Support for attending meetings and/or travel | __x__None | |
| | | | |
| | | | |
| 8 | Patents planned, issued or pending | __x__None | |
| | | | |
| | | | |
| 9 | Participation on a Data Safety Monitoring Board or Advisory Board | __x__None | |
| | | | |
| | | | |
| 10 | Leadership or fiduciary role in other board, society, committee or advocacy group, paid or unpaid | __x__None | |
| | | | |
| | | | |
| 11 | Stock or stock options | __x__None | |
| | | | |
| | | | |
| 12 | Receipt of equipment, materials, drugs, medical writing, gifts or other services | __x__None | |
| | | | |
| | | | |
| 13 | Other financial or non-financial interests | __x__None | |
| | | | |
| | | | |

**Please place an "X" next to the following statement to indicate your agreement:**

**X I certify that I have answered every question and have not altered the wording of any of the questions on this form.**

# ICMJE DISCLOSURE FORM

**Date:______16/04/2024____________________________________________________________**
**Your Name:____Leon Chang_________________________________________________________**
**Manuscript Title:** Characterization of genetic landscape and novel inflammatory biomarkers in patients with adult-onset Still's disease
**Manuscript number (if known):_____** ar-23-1618.R1**_________________________________**

**In the interest of transparency, we ask you to disclose all relationships/activities/interests listed below that are related to the content of your manuscript. "Related" means any relation with for-profit or not-for-profit third parties whose interests may be affected by the content of the manuscript. Disclosure represents a commitment to transparency and does not necessarily indicate a bias. If you are in doubt about whether to list a relationship/activity/interest, it is preferable that you do so.**

**The following questions apply to the author's relationships/activities/interests as they relate to the current manuscript only.**

**The author's relationships/activities/interests should be defined broadly. For example, if your manuscript pertains to the epidemiology of hypertension, you should declare all relationships with manufacturers of antihypertensive medication, even if that medication is not mentioned in the manuscript.**

**In item #1 below, report all support for the work reported in this manuscript without time limit. For all other items, the time frame for disclosure is the past 36 months.**

| | | **Name all entities with whom you have this relationship or indicate none (add rows as needed)** | **Specifications/Comments (e.g., if payments were made to you or to your institution)** |
|---|---|---|---|
| **Time frame: Since the initial planning of the work** | | | |
| 1 | All support for the present manuscript (e.g., funding, provision of study materials, medical writing, article processing charges, etc.) **No time limit for this item.** | __x__None | |
| | | | |
| | | | |
| | | | |
| | | | |
| | | | |
| | | | |
| | | | |
| **Time frame: past 36 months** | | | |
| 2 | Grants or contracts from any entity (if not indicated in item #1 above). | __x__None | |
| | | | |
| | | | |
| 3 | Royalties or licenses | __x__None | |
| | | | |
| | | | |
| 4 | Consulting fees | __x__None | |
| | | | |
| | | | |

| | | | |
|---|---|---|---|
| 5 | Payment or honoraria for lectures, presentations, speakers bureaus, manuscript writing or educational events | __x__None | |
| | | | |
| | | | |
| 6 | Payment for expert testimony | __x__None | |
| | | | |
| | | | |
| 7 | Support for attending meetings and/or travel | __x__None | |
| | | | |
| | | | |
| 8 | Patents planned, issued or pending | __x__None | |
| | | | |
| | | | |
| 9 | Participation on a Data Safety Monitoring Board or Advisory Board | __x__None | |
| | | | |
| | | | |
| 10 | Leadership or fiduciary role in other board, society, committee or advocacy group, paid or unpaid | __x__None | |
| | | | |
| | | | |
| 11 | Stock or stock options | __x__None | |
| | | | |
| | | | |
| 12 | Receipt of equipment, materials, drugs, medical writing, gifts or other services | __x__None | |
| | | | |
| | | | |
| 13 | Other financial or non-financial interests | __x__None | |
| | | | |
| | | | |

**Please place an "X" next to the following statement to indicate your agreement:**

**X I certify that I have answered every question and have not altered the wording of any of the questions on this form.**

## ICMJE DISCLOSURE FORM

**Date:______16/04/2024___________________________________________________________________**
**Your Name:____Linda P Pournara_______________________________________________________________**
**Manuscript Title:** Characterization of genetic landscape and novel inflammatory biomarkers in patients with adult-onset Still's disease
**Manuscript number (if known):_____** ar-23-1618.R1_____________________________________________

**In the interest of transparency, we ask you to disclose all relationships/activities/interests listed below that are related to the content of your manuscript. "Related" means any relation with for-profit or not-for-profit third parties whose interests may be affected by the content of the manuscript. Disclosure represents a commitment to transparency and does not necessarily indicate a bias. If you are in doubt about whether to list a relationship/activity/interest, it is preferable that you do so.**

**The following questions apply to the author's relationships/activities/interests as they relate to the current manuscript only.**

**The author's relationships/activities/interests should be defined broadly. For example, if your manuscript pertains to the epidemiology of hypertension, you should declare all relationships with manufacturers of antihypertensive medication, even if that medication is not mentioned in the manuscript.**

**In item #1 below, report all support for the work reported in this manuscript without time limit. For all other items, the time frame for disclosure is the past 36 months.**

| | | **Name all entities with whom you have this relationship or indicate none (add rows as needed)** | **Specifications/Comments (e.g., if payments were made to you or to your institution)** |
|---|---|---|---|
| **Time frame: Since the initial planning of the work** | | | |
| 1 | All support for the present manuscript (e.g., funding, provision of study materials, medical writing, article processing charges, etc.) **No time limit for this item.** | __x__None | |
| | | | |
| | | | |
| | | | |
| | | | |
| | | | |
| | | | |
| | | | |
| **Time frame: past 36 months** | | | |
| 2 | Grants or contracts from any entity (if not indicated in item #1 above). | __x__None | |
| | | | |
| | | | |
| 3 | Royalties or licenses | __x__None | |
| | | | |
| | | | |
| 4 | Consulting fees | __x__None | |
| | | | |
| | | | |

| | | | |
|---|---|---|---|
| 5 | Payment or honoraria for lectures, presentations, speakers bureaus, manuscript writing or educational events | __x__None | |
| | | | |
| | | | |
| 6 | Payment for expert testimony | __x__None | |
| | | | |
| | | | |
| 7 | Support for attending meetings and/or travel | __x__None | |
| | | | |
| | | | |
| 8 | Patents planned, issued or pending | __x__None | |
| | | | |
| | | | |
| 9 | Participation on a Data Safety Monitoring Board or Advisory Board | __x__None | |
| | | | |
| | | | |
| 10 | Leadership or fiduciary role in other board, society, committee or advocacy group, paid or unpaid | __x__None | |
| | | | |
| | | | |
| 11 | Stock or stock options | __x__None | |
| | | | |
| | | | |
| 12 | Receipt of equipment, materials, drugs, medical writing, gifts or other services | __x__None | |
| | | | |
| | | | |
| 13 | Other financial or non-financial interests | __x__None | |
| | | | |
| | | | |

**Please place an "X" next to the following statement to indicate your agreement:**

**X I certify that I have answered every question and have not altered the wording of any of the questions on this form.**

## ICMJE DISCLOSURE FORM

**Date:______16/04/2024___________________________________________________________**
**Your Name:____Michael F McDermott_____________________________________________________________**
**Manuscript Title:** Characterization of genetic landscape and novel inflammatory biomarkers in patients with adult-onset Still's disease
**Manuscript number (if known):_____** ar-23-1618.R1_____________________________________

**In the interest of transparency, we ask you to disclose all relationships/activities/interests listed below that are related to the content of your manuscript. "Related" means any relation with for-profit or not-for-profit third parties whose interests may be affected by the content of the manuscript. Disclosure represents a commitment to transparency and does not necessarily indicate a bias. If you are in doubt about whether to list a relationship/activity/interest, it is preferable that you do so.**

**The following questions apply to the author's relationships/activities/interests as they relate to the current manuscript only.**

**The author's relationships/activities/interests should be defined broadly. For example, if your manuscript pertains to the epidemiology of hypertension, you should declare all relationships with manufacturers of antihypertensive medication, even if that medication is not mentioned in the manuscript.**

**In item #1 below, report all support for the work reported in this manuscript without time limit. For all other items, the time frame for disclosure is the past 36 months.**

| | | **Name all entities with whom you have this relationship or indicate none (add rows as needed)** | **Specifications/Comments (e.g., if payments were made to you or to your institution)** |
|---|---|---|---|
| | | **Time frame: Since the initial planning of the work** | |
| 1 | All support for the present manuscript (e.g., funding, provision of study materials, medical writing, article processing charges, etc.) **No time limit for this item.** | __x__None | |
| | | | |
| | | | |
| | | | |
| | | | |
| | | | |
| | | | |
| | | | |
| | | **Time frame: past 36 months** | |
| 2 | Grants or contracts from any entity (if not indicated in item #1 above). | __x__None | |
| | | | |
| | | | |
| 3 | Royalties or licenses | __x__None | |
| | | | |
| | | | |
| 4 | Consulting fees | __x__None | |
| | | | |
| | | | |

| | | | |
|---|---|---|---|
| 5 | Payment or honoraria for lectures, presentations, speakers bureaus, manuscript writing or educational events | __x__None | |
| | | | |
| | | | |
| 6 | Payment for expert testimony | __x__None | |
| | | | |
| | | | |
| 7 | Support for attending meetings and/or travel | __x__None | |
| | | | |
| | | | |
| 8 | Patents planned, issued or pending | __x__None | |
| | | | |
| | | | |
| 9 | Participation on a Data Safety Monitoring Board or Advisory Board | __x__None | |
| | | | |
| | | | |
| 10 | Leadership or fiduciary role in other board, society, committee or advocacy group, paid or unpaid | __x__None | |
| | | | |
| | | | |
| 11 | Stock or stock options | __x__None | |
| | | | |
| | | | |
| 12 | Receipt of equipment, materials, drugs, medical writing, gifts or other services | __x__None | |
| | | | |
| | | | |
| 13 | Other financial or non-financial interests | __x__None | |
| | | | |
| | | | |

**Please place an "X" next to the following statement to indicate your agreement:**

**X I certify that I have answered every question and have not altered the wording of any of the questions on this form.**

## ICMJE DISCLOSURE FORM

**Date:______16/04/2024________________________________________________________________**
**Your Name:____Paul A Lyons ______________________________________________________________**
**Manuscript Title:** Characterization of genetic landscape and novel inflammatory biomarkers in patients with adult-onset Still's disease
**Manuscript number (if known):_____** ar-23-1618.R1**_________________________________________**

**In the interest of transparency, we ask you to disclose all relationships/activities/interests listed below that are related to the content of your manuscript. "Related" means any relation with for-profit or not-for-profit third parties whose interests may be affected by the content of the manuscript. Disclosure represents a commitment to transparency and does not necessarily indicate a bias. If you are in doubt about whether to list a relationship/activity/interest, it is preferable that you do so.**

**The following questions apply to the author's relationships/activities/interests as they relate to the <u>current manuscript only</u>.**

**The author's relationships/activities/interests should be <u>defined broadly</u>. For example, if your manuscript pertains to the epidemiology of hypertension, you should declare all relationships with manufacturers of antihypertensive medication, even if that medication is not mentioned in the manuscript.**

**In item #1 below, report all support for the work reported in this manuscript without time limit. For all other items, the time frame for disclosure is the past 36 months.**

| | | **Name all entities with whom you have this relationship or indicate none (add rows as needed)** | **Specifications/Comments (e.g., if payments were made to you or to your institution)** |
|---|---|---|---|
| **Time frame: Since the initial planning of the work** | | | |
| 1 | All support for the present manuscript (e.g., funding, provision of study materials, medical writing, article processing charges, etc.) **No time limit for this item.** | __x__None | |
| | | | |
| | | | |
| | | | |
| | | | |
| | | | |
| | | | |
| | | | |
| **Time frame: past 36 months** | | | |
| 2 | Grants or contracts from any entity (if not indicated in item #1 above). | __x__None | |
| | | | |
| | | | |
| 3 | Royalties or licenses | __x__None | |
| | | | |
| | | | |
| 4 | Consulting fees | __x__None | |
| | | | |
| | | | |

| | | | |
|---|---|---|---|
| 5 | Payment or honoraria for lectures, presentations, speakers bureaus, manuscript writing or educational events | __x__None | |
| | | | |
| | | | |
| 6 | Payment for expert testimony | __x__None | |
| | | | |
| | | | |
| 7 | Support for attending meetings and/or travel | __x__None | |
| | | | |
| | | | |
| 8 | Patents planned, issued or pending | __x__None | |
| | | | |
| | | | |
| 9 | Participation on a Data Safety Monitoring Board or Advisory Board | __x__None | |
| | | | |
| | | | |
| 10 | Leadership or fiduciary role in other board, society, committee or advocacy group, paid or unpaid | __x__None | |
| | | | |
| | | | |
| 11 | Stock or stock options | __x__None | |
| | | | |
| | | | |
| 12 | Receipt of equipment, materials, drugs, medical writing, gifts or other services | __x__None | |
| | | | |
| | | | |
| 13 | Other financial or non-financial interests | __x__None | |
| | | | |
| | | | |

**Please place an "X" next to the following statement to indicate your agreement:**

**X I certify that I have answered every question and have not altered the wording of any of the questions on this form.**

## ICMJE DISCLOSURE FORM

**Date:______16/04/2024________________________________________________________________**
**Your Name:____Rebecca L Ross ___________________________________________________________**
**Manuscript Title:** Characterization of genetic landscape and novel inflammatory biomarkers in patients with adult-onset Still's disease
**Manuscript number (if known):_____** ar-23-1618.R1__________________________________________

**In the interest of transparency, we ask you to disclose all relationships/activities/interests listed below that are related to the content of your manuscript. "Related" means any relation with for-profit or not-for-profit third parties whose interests may be affected by the content of the manuscript. Disclosure represents a commitment to transparency and does not necessarily indicate a bias. If you are in doubt about whether to list a relationship/activity/interest, it is preferable that you do so.**

**The following questions apply to the author's relationships/activities/interests as they relate to the <u>current manuscript only</u>.**

**The author's relationships/activities/interests should be <u>defined broadly</u>. For example, if your manuscript pertains to the epidemiology of hypertension, you should declare all relationships with manufacturers of antihypertensive medication, even if that medication is not mentioned in the manuscript.**

**In item #1 below, report all support for the work reported in this manuscript without time limit. For all other items, the time frame for disclosure is the past 36 months.**

| | | **Name all entities with whom you have this relationship or indicate none (add rows as needed)** | **Specifications/Comments (e.g., if payments were made to you or to your institution)** |
|---|---|---|---|
| **Time frame: Since the initial planning of the work** | | | |
| 1 | All support for the present manuscript (e.g., funding, provision of study materials, medical writing, article processing charges, etc.) **No time limit for this item.** | __x__None | |
| | | | |
| | | | |
| | | | |
| | | | |
| | | | |
| | | | |
| | | | |
| **Time frame: past 36 months** | | | |
| 2 | Grants or contracts from any entity (if not indicated in item #1 above). | __x__None | |
| | | | |
| | | | |
| 3 | Royalties or licenses | __x__None | |
| | | | |
| | | | |
| 4 | Consulting fees | __x__None | |
| | | | |
| | | | |

| | | | |
|---|---|---|---|
| 5 | Payment or honoraria for lectures, presentations, speakers bureaus, manuscript writing or educational events | __x__None | |
| | | | |
| | | | |
| 6 | Payment for expert testimony | __x__None | |
| | | | |
| | | | |
| 7 | Support for attending meetings and/or travel | __x__None | |
| | | | |
| | | | |
| 8 | Patents planned, issued or pending | __x__None | |
| | | | |
| | | | |
| 9 | Participation on a Data Safety Monitoring Board or Advisory Board | __x__None | |
| | | | |
| | | | |
| 10 | Leadership or fiduciary role in other board, society, committee or advocacy group, paid or unpaid | __x__None | |
| | | | |
| | | | |
| 11 | Stock or stock options | __x__None | |
| | | | |
| | | | |
| 12 | Receipt of equipment, materials, drugs, medical writing, gifts or other services | __x__None | |
| | | | |
| | | | |
| 13 | Other financial or non-financial interests | __x__None | |
| | | | |
| | | | |

**Please place an "X" next to the following statement to indicate your agreement:**

**X I certify that I have answered every question and have not altered the wording of any of the questions on this form.**

## ICMJE DISCLOSURE FORM

**Date:______25/08/2024_______________________________________________________________**
**Your Name:____Samuel Lara-Reyna____________________________________________________________**
**Manuscript Title:** Characterization of genetic landscape and novel inflammatory biomarkers in patients with adult-onset Still's disease
**Manuscript number (if known):_____** ar-23-1618.R1__________________________________________

**In the interest of transparency, we ask you to disclose all relationships/activities/interests listed below that are related to the content of your manuscript. "Related" means any relation with for-profit or not-for-profit third parties whose interests may be affected by the content of the manuscript. Disclosure represents a commitment to transparency and does not necessarily indicate a bias. If you are in doubt about whether to list a relationship/activity/interest, it is preferable that you do so.**

**The following questions apply to the author's relationships/activities/interests as they relate to the current manuscript only.**

**The author's relationships/activities/interests should be defined broadly. For example, if your manuscript pertains to the epidemiology of hypertension, you should declare all relationships with manufacturers of antihypertensive medication, even if that medication is not mentioned in the manuscript.**

**In item #1 below, report all support for the work reported in this manuscript without time limit. For all other items, the time frame for disclosure is the past 36 months.**

| | | **Name all entities with whom you have this relationship or indicate none (add rows as needed)** | **Specifications/Comments (e.g., if payments were made to you or to your institution)** |
|---|---|---|---|
| | | **Time frame: Since the initial planning of the work** | |
| 1 | All support for the present manuscript (e.g., funding, provision of study materials, medical writing, article processing charges, etc.) **No time limit for this item.** | __x__None | |
| | | | |
| | | | |
| | | | |
| | | | |
| | | | |
| | | | |
| | | | |
| | | **Time frame: past 36 months** | |
| 2 | Grants or contracts from any entity (if not indicated in item #1 above). | __x__None | |
| | | | |
| | | | |
| 3 | Royalties or licenses | __x__None | |
| | | | |
| | | | |
| 4 | Consulting fees | __x__None | |
| | | | |
| | | | |

| | | | |
|---|---|---|---|
| 5 | Payment or honoraria for lectures, presentations, speakers bureaus, manuscript writing or educational events | __x__None | |
| | | | |
| | | | |
| 6 | Payment for expert testimony | __x__None | |
| | | | |
| | | | |
| 7 | Support for attending meetings and/or travel | __x__None | |
| | | | |
| | | | |
| 8 | Patents planned, issued or pending | __x__None | |
| | | | |
| | | | |
| 9 | Participation on a Data Safety Monitoring Board or Advisory Board | __x__None | |
| | | | |
| | | | |
| 10 | Leadership or fiduciary role in other board, society, committee or advocacy group, paid or unpaid | __x__None | |
| | | | |
| | | | |
| 11 | Stock or stock options | __x__None | |
| | | | |
| | | | |
| 12 | Receipt of equipment, materials, drugs, medical writing, gifts or other services | __x__None | |
| | | | |
| | | | |
| 13 | Other financial or non-financial interests | __x__None | |
| | | | |
| | | | |

**Please place an "X" next to the following statement to indicate your agreement:**

**X I certify that I have answered every question and have not altered the wording of any of the questions on this form.**

# ICMJE DISCLOSURE FORM

**Date:______16/04/2024________________________________________________________________________**
**Your Name:____Sinisa Savic ____________________________________________________________________**
**Manuscript Title:** Characterization of genetic landscape and novel inflammatory biomarkers in patients with adult-onset Still's disease
**Manuscript number (if known):_____** ar-23-1618.R1___________________________________________

**In the interest of transparency, we ask you to disclose all relationships/activities/interests listed below that are related to the content of your manuscript. "Related" means any relation with for-profit or not-for-profit third parties whose interests may be affected by the content of the manuscript. Disclosure represents a commitment to transparency and does not necessarily indicate a bias. If you are in doubt about whether to list a relationship/activity/interest, it is preferable that you do so.**

**The following questions apply to the author's relationships/activities/interests as they relate to the current manuscript only.**

**The author's relationships/activities/interests should be defined broadly. For example, if your manuscript pertains to the epidemiology of hypertension, you should declare all relationships with manufacturers of antihypertensive medication, even if that medication is not mentioned in the manuscript.**

**In item #1 below, report all support for the work reported in this manuscript without time limit. For all other items, the time frame for disclosure is the past 36 months.**

| | | **Name all entities with whom you have this relationship or indicate none (add rows as needed)** | **Specifications/Comments (e.g., if payments were made to you or to your institution)** |
|---|---|---|---|
| | | **Time frame: Since the initial planning of the work** | |
| 1 | All support for the present manuscript (e.g., funding, provision of study materials, medical writing, article processing charges, etc.) **No time limit for this item.** | __x__None | |
| | | | |
| | | | |
| | | | |
| | | | |
| | | | |
| | | | |
| | | | |
| | | **Time frame: past 36 months** | |
| 2 | Grants or contracts from any entity (if not indicated in item #1 above). | Novartis | Personal payment |
| | | Kennedy trust | Personal payment |
| | | | |
| 3 | Royalties or licenses | __x__None | |
| | | | |
| | | | |
| 4 | Consulting fees | Novartis Pharma | Personal payment |
| | | Swedish Orphan Biovitrium | Personal payment |
| | | | |

| | | | |
|---|---|---|---|
| 5 | Payment or honoraria for lectures, presentations, speakers bureaus, manuscript writing or educational events | Takeda | Personal payment |
| | | | |
| | | | |
| 6 | Payment for expert testimony | __x__None | |
| | | | |
| | | | |
| 7 | Support for attending meetings and/or travel | Novartis | Personal payment |
| | | | |
| | | | |
| 8 | Patents planned, issued or pending | __x__None | |
| | | | |
| | | | |
| 9 | Participation on a Data Safety Monitoring Board or Advisory Board | Novartis | Personal payment |
| | | | |
| | | | |
| 10 | Leadership or fiduciary role in other board, society, committee or advocacy group, paid or unpaid | British Society for immunolgy | |
| | | | |
| | | | |
| 11 | Stock or stock options | __x__None | |
| | | | |
| | | | |
| 12 | Receipt of equipment, materials, drugs, medical writing, gifts or other services | __x__None | |
| | | | |
| | | | |
| 13 | Other financial or non-financial interests | __x__None | |
| | | | |
| | | | |

**Please place an "X" next to the following statement to indicate your agreement:**

**X I certify that I have answered every question and have not altered the wording of any of the questions on this form.**
