# Supplementary material for: Characterization of Genetic Landscape and Novel Inflammatory Biomarkers in Patients With Adult‐Onset Still's Disease
Source: Arthritis Rheumatol. 2024 Dec 16;77(5):582–95. doi: 10.1002/art.43054 (PMC12039473; doi:10.1002/art.43054)
Supplement: Supplementary file 2 — Appendix S1. Supporting Information [file ART-77-582-s008.docx]

**Methods**

**Patients and healthy control cohorts**

Three distinct cohorts of AOSD patients were included in this study. Patients in **Cohort 1** (AOSD#1) were recruited via ImmunAID (IMMUNome project consortium for AutoInflammatory Disorders)^1^. The complete set of inclusion and exclusion criteria for this cohort is detailed in the Supplemental methods. In summary, these patients had active disease at the time of enrolment, satisfied either Yamaguchi’s or Fautrel’s criteria, and were not receiving active therapy. Most patients had newly presented with AOSD, while a minority were presenting with a new flair, having previously been in long-term, off-treatment remission. The patients in **Cohort 2** (AOSD#2) were recruited from a single centre in Germany (Erlangen). These patients were recruited consecutively, and diagnoses established according to the Yamaguchi’s criteria after exclusion of infection, malignancy, or other rheumatological disorders. **Cohort 3** (AOSD#3) consisted of patients who took part in the CONSIDER trial (Canakinumab for Treatment of Adult-Onset Still’s Disease to Achieve Reduction of Arthritic Manifestation: phase II, randomised, double-blind, placebo-controlled, multicentre, investigator-initiated trial). Full inclusion and exclusion criteria for this study are provided in the published trial manuscript^2^. In summary, these were patients, with a chronic, therapy resistant and arthritis predominant form of AOSD, who had previously failed several standard and biological disease modifying anti-rheumatic drugs (DMARD). Disease controls include patients with genetically defined and polygenic autoinflammatory disorders: systemic-onset Juvenile Idiopathic Arthritis (sJIA, n=12), cryopyrin associated periodic syndrome (CAPS, n=11), Schnitzler’s syndrome (SchS, n=10) and familial Mediterranean fever (FMF, n=31), all recruited via the ImmunAID study.

The disease activity in AOSD patients was determined using novel Still’s activity score validated by Kalyoncu et al^3^. One of the main considerations for using this scoring system was availability of clinical data in this retrospective study. We determined that on balance SAS will not only be informative, but it is also possible to determine for the majority of patients in the study.

***Exome sequencing and data pre-processing***

DNA extracted from whole blood was sent to Novogene Cambridge for whole exome sequencing (WES, target >100× mean read depth). DNA was quality checked using the Qubit® and NanoDrop before libraries were created for paired-end 150bp sequencing using the NovaSeq 6000 platform (Illumina Inc.). The resulting data was processed using an in-house bioinformatics pipeline. Aligned BAM files were generated using bwa-mem version 0.7.17 with sequence reads being mapped to the human reference genome (build GRCh38/hg38)^4^; before being sorted by alignment coordinate and PCR duplicate marking using samtools/picard version 3.1.0. Variant identification was performed in accordance with GATK best practice guidelines^5^.

***Custom gene panel***

Targeted variant calling was achieved using a custom panel of 139 genes divided into three sub-categories in association with CHIP (87 genes), autoinflammation (38 genes) and Type I interferonopathies (14 genes). The full gene panel can be viewed in Supplemental Table S2.

***Germline variant discovery***

Germline SNP and Indel variant calling was achieved using the HaplotypeCaller. An AOSD cohort GVCF was passed through the genotyping tool before undergoing variant quality score recalibration (VQSR) to reduce the number of false positive variants. Post-VQSR variants were annotated with population allele frequencies from Genome Aggregation Database (gnomAD) v.4.0.0 and functionally annotated using the Ensembl Variant Effect Predictor (VEP). Each variant was assigned a SIFT, PolyPhen-2 and CADD score to aid with (data) interpretation. Germline variants were filtered with VASE^6^, using the following criteria: <1% population allele frequency in gnomAD, CADD>20, DP> 8, GQ>20 and VQSR Filter= PASS. This incidence of all germline variants called within our gene panel was compared against their prevalence within gnomAD v4.0.0 which hosts sequencing data from 590,031 individuals of European Non-Finnish ancestry.

***Somatic variant discovery***

Mutect2 was run on individual exomes in tumour-only mode, using the human reference genome (build GRCh38/hg38). Variant sites were pre-filtered for common germline variants and sequencing artifacts, using a, in-house ‘panel of normals’ created from the WES data of 49 healthy controls (HC) and a population germline resource supplied by GATK (gs://gatk-best-practices/somatic-hg38). Cross-sample contamination was estimated, using GetPileupSummaries, and this information was used with FilterMutectCalls to define somatic variants, with a degree of confidence. Putative somatic variants must have ≥69x total depth and at least 5 mutant reads (ensuring >5% VAF in variants with ≤100 total depth), unless it is a previously confirmed somatic variant reported in COSMIC.

***Bulk Transcriptomics***

Whole blood samples (AOSD#2 n=27, HC n=10) were sent to Novogene Cambridge for total RNA extraction and RNA sequencing (RNAseq). Total RNA samples were quality checked using the Agilent 2100 and NanoDrop, before poly-A enriched 150bp paired-end libraries were created for sequencing on the Illumina NovaSeq 6000 platform. Data trimming and pre-processing was performed in-house and BAM files were created aligning to reference genome GRCh38/hg38 using STAR aligner. PCR duplicates were marked, and gene level read counts were quantified using featureCounts. Gene counts were exported into RStudio and analysed using DESeq2. Differentially expressed genes were defined as those with a log2FoldChange >1 or <-1, and an adjusted p-value <0.001. Data were visualised using ggplot2 and functionally annotated using EnrichR.

**ASC/NLRP3 protein speck assay**

We used in-house flow cytometry assay, for quantification of ASC/NLRP3 protein specks in sera. Briefly, 100µl of patients’ sera was incubated in Phycoerythrin conjugated anti-ASC (TMS-1) Antibody (653904, BioLegend) and APC-conjugated NLRP3 Antibody (IC7578A, BioTechne) for 1 hour at room temperature, with shaking. A 50µl sample volume was measured by using a Cytoflex S Flow Cytometer, gating for events 1µM in size, using Flow Cytometry Sub-micron Particle Size Reference beads (F13839, Thermo-Fisher Scientific). For the detailed gating strategy please see Supplemental Figure S1. Results were analysed using CytExpert software and presented as ASC/NLRP3 positive events/µl.

**Cytokine profile analysis**

The inflammatory profiles of patients in the three different cohorts were investigated using the multiplex LEGENDplex™ Multi-Analyte Flow Assay kit (Biolegend). The human inflammation panel 1 includes IL-1β, IFN-α2, IFN-γ, TNF, CCL2, IL-6, CXCL8, IL-10, IL-12p70, IL-17A, IL-18, 1L-23 and IL-33. Serum samples were all diluted 2-fold in assay buffer prior to analysis and assays carried out according to the manufacturer’s instructions for analysis of serum samples in V-bottom 96-well plates (provided, Biolegend). All standards and samples were run in duplicate. Data were acquired using a BD Canto II flow cytometer (BD Bioscience) and BD Diva software. A total of 1000 events were collected per well using a high throughput 96-well plate autosampler. Analyses were carried out using the LEGENDplex™ Data Analysis Software.

**Type I Interferon score**

The Type I Interferon (IFN) score was investigated using a custom Luminex Discovery Assay (bio-techne, R&D systems) which included the following chemokines: CXCL10, CCL2, CCL8, CCL19 and CXCL11. Serum samples were prepared and assayed according to the manufacturer’s instructions. Briefly, frozen serum samples were thawed, centrifuged at 16000 g for 4 min and diluted 2-fold. All standards and samples were run in duplicate. All incubations were carried out at RT, with shaking at 800 rpm and protected from light. Standards and diluted serum were mixed with an equal volume of the diluted magnetic microparticle cocktail, in a 96 well plate, prior to incubation for 2 h. A magnet was subsequently used to wash the beads a total of three times. Diluted Biotin-antibody cocktail was then added to each well, the plate was incubated protected from light for 1 h at RT. Following this incubation period, the beads were washed again, and diluted Streptavidin-PE was added. The plate was incubated for a final 30 min followed by a final wash. The plate was loaded onto a Luminex 200, configured as per manufacturer’s instructions for the analytes in this assay. Results were generated using the xPONENT software, comparing MFIs of each analyte against a 5-point standard curve to calculate chemokine concentrations (pg/mL). The concentration of the five chemokines were natural logged and averaged to calculate the Type I Interferon scores. Results below the detection limit of the assay for any analyte, were considered to be equal to the lowest detectable concentration, for the purposes of Type I Interferon score calculations.

We also calculated traditional Type I Interferon scores based on the gene expression data generated from RNAseq. We calculated a second IFN score for each patient by adopting the methodology from Kim et al^7^., using the gene expression of 28-Interferon response genes and compared these scores to our novel IFN scoring method. Correlation was assessed by linear regression analysis.

**Statistical analyses**

Differences in patient age distribution were assessed with a one-way ANOVA. Enrichment analysis of genetic variants were performed using a Fisher’s exact test for each variant, using the recorded prevalence in the European Non-Finnish population in gnomAD for comparison. We accepted statistical significance at an adjusted p-value after applying Bonferroni correction for multiple testing (0.05/variant count). Differential gene expression was analysed using DESeq2, and subsequent pathway/ontology analysis was achieved using Enrichr. Statistical analyses of functional data (ASC/NLRP3 specks, ASC (only) specks, SAS, cytokine levels and IFN scores) were calculated using a non-parametric Kruskall-Wallis test, with postdoc Dunn’s test for pairwise comparisons. Except for Figure 4D which involved paired data and was analysed using a Wilcoxon signed-rank test to assess differences before and after treatments.

**Data availability**

Processed and raw RNAseq data is publicly available at time of publication; GEO accession number GSE244372; BioProject PRJNA1022483.

**References**

1 *ImmunAID*, <<https://www.immunaid.eu>> (

2 Kedor, C. *et al.* Canakinumab for Treatment of Adult-Onset Still's Disease to Achieve Reduction of Arthritic Manifestation (CONSIDER): phase II, randomised, double-blind, placebo-controlled, multicentre, investigator-initiated trial. *Ann Rheum Dis* **79**, 1090-1097 (2020). <https://doi.org:10.1136/annrheumdis-2020-217155>

3 Kalyoncu, U. *et al.* Derivation and validation of adult Still Activity Score (SAS). *Joint Bone Spine* **90**, 105499 (2023). <https://doi.org:10.1016/j.jbspin.2022.105499>

4 Li, H. & Durbin, R. Fast and accurate short read alignment with Burrows-Wheeler transform. *Bioinformatics* **25**, 1754-1760 (2009). <https://doi.org:10.1093/bioinformatics/btp324>

5 BD, V. d. A. G. a. O. C. *Genomics in the Cloud: Using Docker, GATK, and WDL in Terra*. 1 st edn, (O'Reilly Media, 2020).

6 Parry, D. *VASE source code*, <<https://github.com/david-a-parry/vase>> (2023).

7 Kim, H. *et al.* Development of a Validated Interferon Score Using NanoString Technology. *J Interferon Cytokine Res* **38**, 171-185 (2018). <https://doi.org:10.1089/jir.2017.0127>
