# Supplementary material for: Characterization of Genetic Landscape and Novel Inflammatory Biomarkers in Patients With Adult‐Onset Still's Disease
Source: Arthritis Rheumatol. 2024 Dec 16;77(5):582–95. doi: 10.1002/art.43054 (PMC12039473; doi:10.1002/art.43054)
Supplement: Supplementary file 3 — Appendix S2. Supporting Information [file ART-77-582-s015.pdf]

Supplementary results (Case description)

A 64-years old male initially presented in 2009 with a history of subcutaneous nodules and fevers. He was originally diagnosed with Sweet's syndrome following a skin biopsy. In 2011 he was admitted due to ongoing fevers and nodular skin rash. Routine investigations showed persistent neutrophilia, elevated CRP, Serum amylod A (SAA) and interleukin (IL)-18. Further investigation which included repeated blood cultures, whole body CT, bone marrow biopsy, endoscopy and colonoscopy, transthoracic ECHO, were all uninformative. Subsequently, a PET-CT was performed which showed multiple FDG-positive foci in the bones of the lower extremities. An MRI of lower limbs demonstrated diffuse bone marrow oedema with adjacent periosteal reaction in the tibial shafts bilaterally. A CT-guided biopsy of affected areas showed no evidence of infection, granulomatous inflammation, or malignancy. In view of ongoing fevers and other negative investigations, patient was empirically treated with anakinra, to which he made good initial response with resolution of fevers, but he continued to complain of night sweats and develop new skin nodules.

In 2015 anakinra was stopped and treatment changed to tocilizumab due to further disease flair. Shortly afterwards, having completed 3 months of treatment, the patient was found to have massive peripheral oedema. His condition worsened and he was admitted with acute renal failure, hypoalbuminemia and asities. A CT scan at the time showed large pleural effusions and multiple cavern-like lesions on his lungs in keeping with likely septic emboli. Following urgent referral to thoracic surgeons, the patient rapidly deteriorated and sadly died due to multi-organ failure and presumed sepsis.
