# Supplementary material for: Characterization of Genetic Landscape and Novel Inflammatory Biomarkers in Patients With Adult‐Onset Still's Disease
Source: Arthritis Rheumatol. 2024 Dec 16;77(5):582–95. doi: 10.1002/art.43054 (PMC12039473; doi:10.1002/art.43054)
Supplement: Supplementary file 4 — Appendix S3. Supporting Information [file ART-77-582-s009.pdf]

Inclusion/Exclusion Criteria review

Trial : ImmunAID Subgroup 1: Still Diseases : Adult onset Still disease / Systemic Juvenile Idiopathic Arthritis

This disease can affect people of any age, all patients older than 6 months can be recruited

Diagnosis Criteria	
**Patient ≥16yo**: satisfaction of original Yamaguchi or Fautrel classification criteria	☐ Yes ☐ No
**Patient <16yo**: satisfaction of the ILAR classification criteria for juvenile idiopathic arthritis (2004 release)	☐ Yes ☐ No
**Non-inclusion (must be no)**	
Active chronic infection included chronic viral infection (HIV, HBV, HCV…)	☐ Yes ☐ No
Recent infection or antibiotic treatment in the last 2 weeks	☐ Yes ☐ No
Systemic auto-immune disease	☐ Yes ☐ No
Other etiology of fever (infection or neoplasia)	☐ Yes ☐ No
Monogenic auto-inflammatory disease (other than FMF, HIDS, TRAPS, CAPS)	☐ Yes ☐ No
Genetic macrophage activation syndrome	☐ Yes ☐ No
Evidence of immuno-deficiency (e.g., neutropenia, HIV transplant recipient, immunosuppressive treatment for other conditions etc.)	☐ Yes ☐ No
Pregnancy	☐ Yes ☐ No
Individuals deprived of liberty	☐ Yes ☐ No
Inability to understand the local language	☐ Yes ☐ No
Protected persons (under guardianship or curatorship)	☐ Yes ☐ No
**Active disease defined as presence of**	
o And CRP ≥ 3 ULN	☐ Yes ☐ No
o And 3 of the following features:<br>▪ arthritis<br>▪ sore throat<br>▪ skin rash<br>▪ lymphadenopathy or splenomegaly<br>▪ increased WBC ≥ 10,000 /mm^3<br>▪ PMN ≥ 80%<br>▪ Fever	☐ Yes ☐ No
Date :_____._____.________	Investigator's Signature

Patient number:______________________　　**Patient name: ______________________**

Trial : ImmunAID Subgroup 2: Recurrent Pericarditis

This disease can affect people of any age, all patients older than 6 months can be recruited

Diagnosis Criteria (must be yes)	
At least 3 episodes of chest pain, ≥ 6 weeks apart	☐ Yes ☐ No
At least 2 of the following criteria:<br>○ Pericarditic-typical chest pain (sharp, pleuritic, improved by sitting up and leaning forward),<br>○ Pericardial friction rubs<br>○ Widespread ST-segment elevation or PR depressions	☐ Yes ☐ No
**Non-inclusion (must be no)**	
Recurrent pericarditis from other causes, including infectious (tuberculosis or other), neoplastic, inflammatory systemic diseases.	☐ Yes ☐ No
Active chronic infection included chronic viral infection (HIV, HBV, HCV…)	☐ Yes ☐ No
Recent infection or antibiotic treatment in the last 2 weeks	☐ Yes ☐ No
Systemic auto-immune disease	☐ Yes ☐ No
Other etiology of fever (infection or neoplasia)	☐ Yes ☐ No
Monogenic auto-inflammatory disease (other than FMF, HIDS, TRAPS, CAPS)	☐ Yes ☐ No
Genetic macrophage activation syndrome	☐ Yes ☐ No
Evidence of immuno-deficiency (e.g., neutropenia, HIV transplant recipient, immunosuppressive treatment for other conditions etc.)	☐ Yes ☐ No
Pregnancy	☐ Yes ☐ No
Individuals deprived of liberty	☐ Yes ☐ No
Inability to understand the local language	☐ Yes ☐ No
Protected persons (under guardianship or curatorship)	☐ Yes ☐ No
**Active disease defined as presence of**	
CRP ≥ 3 ULN	☐ Yes ☐ No
And at least one of the followings:<br>○ Pericardial rub<br>○ ECG changes suggestive of pericarditis<br>○ New or worsening of pericardial effusion	☐ Yes ☐ No
Date :_____._____._______	Investigator's Signature

Patient number:______________________ **Patient name: ______________________**

Trial : ImmunAID Subgroup 3: Neutrophilic Dermatosis

This disease affects almost exclusively adults

Diagnosis Criteria	
Satisfaction of the classification criteria of one of the 2 following diseases:<br>o Pyoderma gangrenosum<br>o Sweet syndrome	☐ Yes ☐ No
**Non-inclusion (must be no)**	
Association with an underlying hematologic or visceral malignancy,	☐ Yes ☐ No
Association with drug exposure	☐ Yes ☐ No
Association with other characterized infections or auto-inflammatory disease or auto-immune diseases	☐ Yes ☐ No
Active chronic infection included chronic viral infection (HIV, HBV, HCV…)	☐ Yes ☐ No
Recent infection or antibiotic treatment in the last 2 weeks	☐ Yes ☐ No
Systemic auto-immune disease	☐ Yes ☐ No
Other etiology of fever (infection or neoplasia)	☐ Yes ☐ No
Monogenic auto-inflammatory disease (other than FMF, HIDS, TRAPS, CAPS)	☐ Yes ☐ No
Genetic macrophage activation syndrome	☐ Yes ☐ No
Evidence of immuno-deficiency (e.g., neutropenia, HIV transplant recipient, immunosuppressive treatment for other conditions etc.)	☐ Yes ☐ No
Pregnancy	☐ Yes ☐ No
Individuals deprived of liberty	☐ Yes ☐ No
Inability to understand the local language	☐ Yes ☐ No
Protected persons (under guardianship or curatorship)	☐ Yes ☐ No
**Active disease defined as presence of**	
Presence of active skin lesion	☐ Yes ☐ No
CRP ≥ 3ULN	☐ Yes ☐ No
Date :_____._____._______	Investigator’s Signature

Patient number:______________________ **Patient name:** ______________________

Trial : ImmunAID Subgroup 4: Schnitzler

This disease affects almost exclusively adults

Diagnosis Criteria	
Satisfaction of the Strasbourg classification criteria	☐ Yes ☐ No
**Non-inclusion (must be no)**	
Active chronic infection included chronic viral infection (HIV, HBV, HCV…)	☐ Yes ☐ No
Recent infection or antibiotic treatment in the last 2 weeks	☐ Yes ☐ No
Systemic auto-immune disease	☐ Yes ☐ No
Other etiology of fever (infection or neoplasia)	☐ Yes ☐ No
Monogenic auto-inflammatory disease (other than FMF, HIDS, TRAPS, CAPS)	☐ Yes ☐ No
Genetic macrophage activation syndrome	☐ Yes ☐ No
Evidence of immuno-deficiency (e.g., neutropenia, HIV transplant recipient, immunosuppressive treatment for other conditions etc.)	☐ Yes ☐ No
Pregnancy	☐ Yes ☐ No
Individuals deprived of liberty	☐ Yes ☐ No
Inability to understand the local language	☐ Yes ☐ No
Protected persons (under guardianship or curatorship)	☐ Yes ☐ No
**Active disease defined as presence of**	
• CRP ≥ 3ULN	☐ Yes ☐ No
3 of the following features:<br>▪ arthritis<br>▪ urticarial skin rash<br>▪ bone pain<br>▪ lymphadenopathy<br>▪ increased WBC ≥ 10,000 /mm^3<br>▪ PMN ≥ 80%<br>▪ Fever	☐ Yes ☐ No
Date :_____._____.________	Investigator's Signature

Patient number:______________________ Patient name: ______________________

Trial : ImmunAID Subgroup 5: Vasculitis (Kawasaki)

These diseases can affect people of any age, all patients older than 6 months can be recruited

Diagnosis Criteria (must be yes)	
**Patient ≥16yo**: satisfaction of the Kawasaki disease criteria	☐ Yes ☐ No
**Patient <16yo**: satisfaction of the Kawasaki disease criteria of the American heart association for complete and incomplete KD	☐ Yes ☐ No
**Non-inclusion (must be no)**	
Active chronic infection included chronic viral infection (HIV, HBV, HCV…)	☐ Yes ☐ No
Recent infection or antibiotic treatment in the last 2 weeks	☐ Yes ☐ No
Systemic auto-immune disease	☐ Yes ☐ No
Other etiology of fever (infection or neoplasia)	☐ Yes ☐ No
Monogenic auto-inflammatory disease (other than FMF, HIDS, TRAPS, CAPS)	☐ Yes ☐ No
Genetic macrophage activation syndrome	☐ Yes ☐ No
Evidence of immuno-deficiency (e.g., neutropenia, HIV transplant recipient, immunosuppressive treatment for other conditions etc.)	☐ Yes ☐ No
Pregnancy	☐ Yes ☐ No
Individuals deprived of liberty	☐ Yes ☐ No
Inability to understand the local language	☐ Yes ☐ No
Protected persons (under guardianship or curatorship)	☐ Yes ☐ No
**Active disease defined as presence of**	
Physican Global Assesst (PhGA) ≥ 2/10	☐ Yes ☐ No
Patient/Parent Global Assessment ≥ 2/10	☐ Yes ☐ No
Fever and/or CRP ≥ 3ULN	☐ Yes ☐ No
At least 2 symptoms of the following list<br>o Malaise, pain, irritability<br>o Eye redness conjunctivitis<br>o Erythema of lips or oral cavity<br>o Lymphadenopathy > 15mm<br>o Edema and/or redness of the extremities<br>o Diffuse skin rash (other than vesicles or crusts)<br>o New or increasing coronary dilatation at echocardiogram with a Z score ≥ 2, at any coronary artery<br>o New or increasing peripheral arterial dilatation	☐ Yes ☐ No
Date :_____._____.________	Investigator's Signature

Patient number:______________________ Patient name: ______________________

Trial : ImmunAID Subgroup 5: Vasculitis (Behçet)

These diseases can affect people of any age, all patients older than 6 months can be recruited

Diagnosis Criteria (must be yes)	
**Patient ≥16yo:** satisfaction of the International Criteria for Behçet's Disease (ICBD : *International Team for the Revision of the International Criteria for Behçet's Disease*	☐ Yes ☐ No
**Patient <16yo:** Satisfaction of paediatric criteria for Behçet disease	☐ Yes ☐ No
**Non-inclusion (must be no)**	
Active chronic infection included chronic viral infection (HIV, HBV, HCV…)	☐ Yes ☐ No
Recent infection or antibiotic treatment in the last 2 weeks	☐ Yes ☐ No
Systemic auto-immune disease	☐ Yes ☐ No
Other etiology of fever (infection or neoplasia)	☐ Yes ☐ No
Monogenic auto-inflammatory disease (other than FMF, HIDS, TRAPS, CAPS)	☐ Yes ☐ No
Genetic macrophage activation syndrome	☐ Yes ☐ No
Evidence of immuno-deficiency (e.g., neutropenia, HIV transplant recipient, immunosuppressive treatment for other conditions etc.)	☐ Yes ☐ No
Pregnancy	☐ Yes ☐ No
Individuals deprived of liberty	☐ Yes ☐ No
Inability to understand the local language	☐ Yes ☐ No
Protected persons (under guardianship or curatorship)	☐ Yes ☐ No
**Active disease defined as presence of**	
PhGA of clinical symptoms ≥ 2/10	☐ Yes ☐ No
Patient/Parent Global Assessment ≥ 2/10	☐ Yes ☐ No
Fever and/or CRP > ULN	☐ Yes ☐ No
Date :_____._____._______	Investigator's Signature

Patient number:______________________   Patient name: _____________________

Trial : ImmunAID Subgroup 5: Vasculitis (Takayasu)

These diseases can affect people of any age, all patients older than 6 months can be recruited

Diagnosis Criteria (must be yes)	
**Patient ≥16yo:** Satisfaction of the Takayasu disease criteria	☐ Yes ☐ No
**Patient <16yo:** Satisfaction of the EULAR/Printo/PReS classification for childhood Takayasu arteritis	☐ Yes ☐ No
**Non-inclusion (must be no)**	
Active chronic infection included chronic viral infection (HIV, HBV, HCV…)	☐ Yes ☐ No
Recent infection or antibiotic treatment in the last 2 weeks	☐ Yes ☐ No
Systemic auto-immune disease	☐ Yes ☐ No
Other etiology of fever (infection or neoplasia)	☐ Yes ☐ No
Monogenic auto-inflammatory disease (other than FMF, HIDS, TRAPS, CAPS)	☐ Yes ☐ No
Genetic macrophage activation syndrome	☐ Yes ☐ No
Evidence of immuno-deficiency (e.g., neutropenia, HIV transplant recipient, immunosuppressive treatment for other conditions etc.)	☐ Yes ☐ No
Pregnancy	☐ Yes ☐ No
Individuals deprived of liberty	☐ Yes ☐ No
Inability to understand the local language	☐ Yes ☐ No
Protected persons (under guardianship or curatorship)	☐ Yes ☐ No
**Active disease defined as presence of**	
PhGA of clinical symptoms ≥ 2/10	☐ Yes ☐ No
Disease activity defined according to the NIH criteria:<br>• New ischemic vascular sign (i.e. claudication, bruit or asymmetry in pulses or blood pressure, carotidynia, pulse abolition);<br>• New arterial lesion or worsening of pre- existing lesions on imaging, visualized by echography, angio-CT and/or MRI;<br>• Systemic clinical features (i.e. weight loss, fever, or myalgia); and<br>• Biological activity of disease: increased erythrocyte sedimentation rate (ESR) and/or of C-reactive protein (CRP).<br><br>Disease will be considered as active if the NIH score was >1, and inactive in the remaining cases.	☐ Yes ☐ No
Date :_____._____._______	Investigator's Signature

Patient number:______________________ **Patient name:** ______________________

Trial : ImmunAID Subgroup 6: Inflammation of unknown origin

These diseases can affect people of any age, all patients older than 6 months can be recruited

Diagnosis Criteria (must be yes)	
Temperature > 38°C during flare	☐ Yes ☐ No
CRP ≥ 30 mg/L or SAA > 25mg/L on at least 3 occasions separated by 2 weeks	☐ Yes ☐ No
Symptom occurring over a period of > 1 months	☐ Yes ☐ No
Suggestive of a systemic autoinflammatory conditions	☐ Yes ☐ No
**Non-inclusion (must be no)**	
Active chronic infection included chronic viral infection (HIV, HBV, HCV…)	☐ Yes ☐ No
Recent infection or antibiotic treatment in the last 2 weeks	☐ Yes ☐ No
Systemic auto-immune disease	☐ Yes ☐ No
Other etiology of fever (infection or neoplasia)	☐ Yes ☐ No
Monogenic auto-inflammatory disease (other than FMF, HIDS, TRAPS, CAPS)	☐ Yes ☐ No
Genetic macrophage activation syndrome	☐ Yes ☐ No
Evidence of immuno-deficiency (e.g., neutropenia, HIV transplant recipient, immunosuppressive treatment for other conditions etc.)	☐ Yes ☐ No
Pregnancy	☐ Yes ☐ No
Individuals deprived of liberty	☐ Yes ☐ No
Inability to understand the local language	☐ Yes ☐ No
Protected persons (under guardianship or curatorship)	☐ Yes ☐ No
**Active disease defined as presence of**	
Fever	☐ Yes ☐ No
CRP ≥ 30 mg/L	☐ Yes ☐ No
PhGA ≥ 2/10	☐ Yes ☐ No
Date :_____._____.________	Investigator's Signature

Patient number:______________________ **Patient name: ______________________**

Trial : ImmunAID Subgroup 7: Chronic/recurrent non bacterial osteitis

These diseases can affect people of any age, all patients older than 6 months can be recruited

Diagnosis Criteria (must be yes)	
Patient fulfilling the criteria for Satisfaction of the non-bacterial osteitis (NBO)	☐ Yes ☐ No
**Non-inclusion (must be no)**	
Other forms of focal hyperostosis (Paget's disease of bone, osseous dysplasia, periostitis, bone metastasis…),	☐ Yes ☐ No
Hypervitaminosis A (if clinically suspected)	☐ Yes ☐ No
Active chronic infection included chronic viral infection (HIV, HBV, HCV…)	☐ Yes ☐ No
Recent infection or antibiotic treatment in the last 2 weeks	☐ Yes ☐ No
Systemic auto-immune disease	☐ Yes ☐ No
Other etiology of fever (infection or neoplasia)	☐ Yes ☐ No
Monogenic auto-inflammatory disease (other than FMF, HIDS, TRAPS, CAPS)	☐ Yes ☐ No
Genetic macrophage activation syndrome	☐ Yes ☐ No
Evidence of immuno-deficiency (e.g., neutropenia, HIV transplant recipient, immunosuppressive treatment for other conditions etc.)	☐ Yes ☐ No
Pregnancy	☐ Yes ☐ No
Individuals deprived of liberty	☐ Yes ☐ No
Inability to understand the local language	☐ Yes ☐ No
Protected persons (under guardianship or curatorship)	☐ Yes ☐ No
**Active disease defined as presence of**	
Pain ≥ 5/10	☐ Yes ☐ No
And/or new lesions on imaging (Positive MRI or bone scan or PET-scan)	☐ Yes ☐ No
And/or CRP ≥ ULN (or ≥ 10 mg/L)	☐ Yes ☐ No
Date :_____._____._______	Investigator's Signature

Patient number:______________________ **Patient name: ______________________**

Trial : ImmunAID: Parents of guSAID patients

Inclusion (must be yes)	
First-degree biological relationship (no adoption) with the index patient	☐ Yes ☐ No
Mother and Father aged more than 18 years old	☐ Yes ☐ No
Health insurance coverage	☐ Yes ☐ No
Signature of the informed consent form	☐ Yes ☐ No
**Non-inclusion (must be no)**	
Any condition which in the Investigator's opinion makes it undesirable for the subject to participate in the study or which would jeopardize compliance with the protocol	☐ Yes ☐ No
Individuals deprived of liberty	☐ Yes ☐ No
Inability to understand the local language	☐ Yes ☐ No
Protected persons (under guardianship or curatorship)	☐ Yes ☐ No
Date :_____._____._______	Investigator's Signature

Patient number:______________________ **Patient name: _____________________**

Trial : ImmunAID: mSAID patients

Inclusion (must be yes)	
Patients with monogenic hereditary SAID: FMF/TRAPS/HIDS/CAPS	☐ Yes ☐ No
Patients diagnosed according to the specific diagnostic criteria of each disease. EUROFEVER criteria *(J Rheumatol 2018, in press)* for clinical definition plus genetic criterion<br>○ FMF: Two pathogenic mutations or at least one pathogenic in the exon 10 of the *MEFV* gene<br>○ CAPS: One pathogenic mutation in *NLRP3* (or mosaicism)<br>○ TRAPS: One pathogenic mutation in *TNFRSF1A* gene<br>○ MKD: Two pathogenic mutations in the *MVK* gene or elevated mevalonaturia	☐ Yes ☐ No
1. Patients with active disease (presence of a flare and/ or persistent chronic inflammation) : ≥ 3 of the 4 following symptoms :<br>○ Fever<br>○ PhGA ≥ 2/10<br>○ Patient AIDAI score ≥ 9 on an at list 29 days period<br>○ CRP ≥ ULN (or ≥ 10 mg/L) or /and SAA≥10 mg/L	☐ Yes ☐ No
This disease can affect people of any age, so all patients older than 6 months can be recruited	☐ Yes ☐ No
Patients with health insurance	☐ Yes ☐ No
Signature of the informed consent form (parents/legal representative if the patient is less than <18 years old)	☐ Yes ☐ No
**Non-inclusion (must be no)**	
Active chronic infection included chronic viral infection (HIV, HBV, HCV…)	☐ Yes ☐ No
Recent infection or antibiotic treatment in the last 2 weeks	☐ Yes ☐ No
Systemic auto-immune disease	☐ Yes ☐ No
Other etiology of fever (infection or neoplasia)	☐ Yes ☐ No
Monogenic auto-inflammatory disease (other than FMF, HIDS, TRAPS, CAPS)	☐ Yes ☐ No
Genetic macrophage activation syndrome	☐ Yes ☐ No
Evidence of immuno-deficiency (e.g. neutropenia, HIV, transplant recipient, immunosuppressive treatment for other conditions etc.)	☐ Yes ☐ No
Pregnancy	☐ Yes ☐ No
Individuals deprived of liberty	☐ Yes ☐ No
Inability to understand the local language	☐ Yes ☐ No
Protected persons (under guardianship or curatorship)	☐ Yes ☐ No
Date :_____._____._______	Investigator's Signature

Trial : ImmunAID: Negative Control

Inclusion (must be yes)	
Subject free of inflammatory disorders and negative CRP at enrollment	☐ Yes ☐ No
Subject without personal or familial history of SAID	☐ Yes ☐ No
Subject aged from 10 to 60 years old	☐ Yes ☐ No
Subject with health insurance	☐ Yes ☐ No
Signature of the informed consent form (parents/legal representative (if the patient is less than <18 years old)	☐ Yes ☐ No
**Non-inclusion (must be no)**	
Active bacterial, viral, fungal, or opportunistic infections	☐ Yes ☐ No
Recent infection or antibiotic treatment in the last 2 weeks	☐ Yes ☐ No
History of any inflammatory, auto-inflammatory or auto-immune disease	☐ Yes ☐ No
History of systemic corticosteroid or non-steroidal (NSAID) therapy within the last 4 weeks	☐ Yes ☐ No
History of neoplasia with the exception of adequately treated basal and squamouscell carcinoma of the skin, or carcinoma in situ of the cervix	☐ Yes ☐ No
Evidence of immunocompromised (e.g. neutropenia, HIV, transplant recipient, immunosuppressive treatment for other conditions etc)	☐ Yes ☐ No
End stage renal disease (eGFR <20 mL/min/1.73m^2)	☐ Yes ☐ No
Comorbidities requiring corticosteroid therapy, including those which have required three or more courses of systemic corticosteroids within the previous 12 months	☐ Yes ☐ No
Pregnancy	☐ Yes ☐ No
Current substance abuse or history of substance abuse within the past year.	☐ Yes ☐ No
Lack of peripheral venous access	☐ Yes ☐ No
Individuals deprived of liberty	☐ Yes ☐ No
Inability to understand the local language	☐ Yes ☐ No
Protected persons (under guardianship or curatorship)	☐ Yes ☐ No
Date :_____._____._______	Investigator's Signature

Patient number:______________________ Patient name: ______________________
