# Supplementary figures and images for: Characterization of Genetic Landscape and Novel Inflammatory Biomarkers in Patients With Adult‐Onset Still's Disease
Source: Arthritis Rheumatol. 2024 Dec 16;77(5):582–95. doi: 10.1002/art.43054 (PMC12039473; doi:10.1002/art.43054)

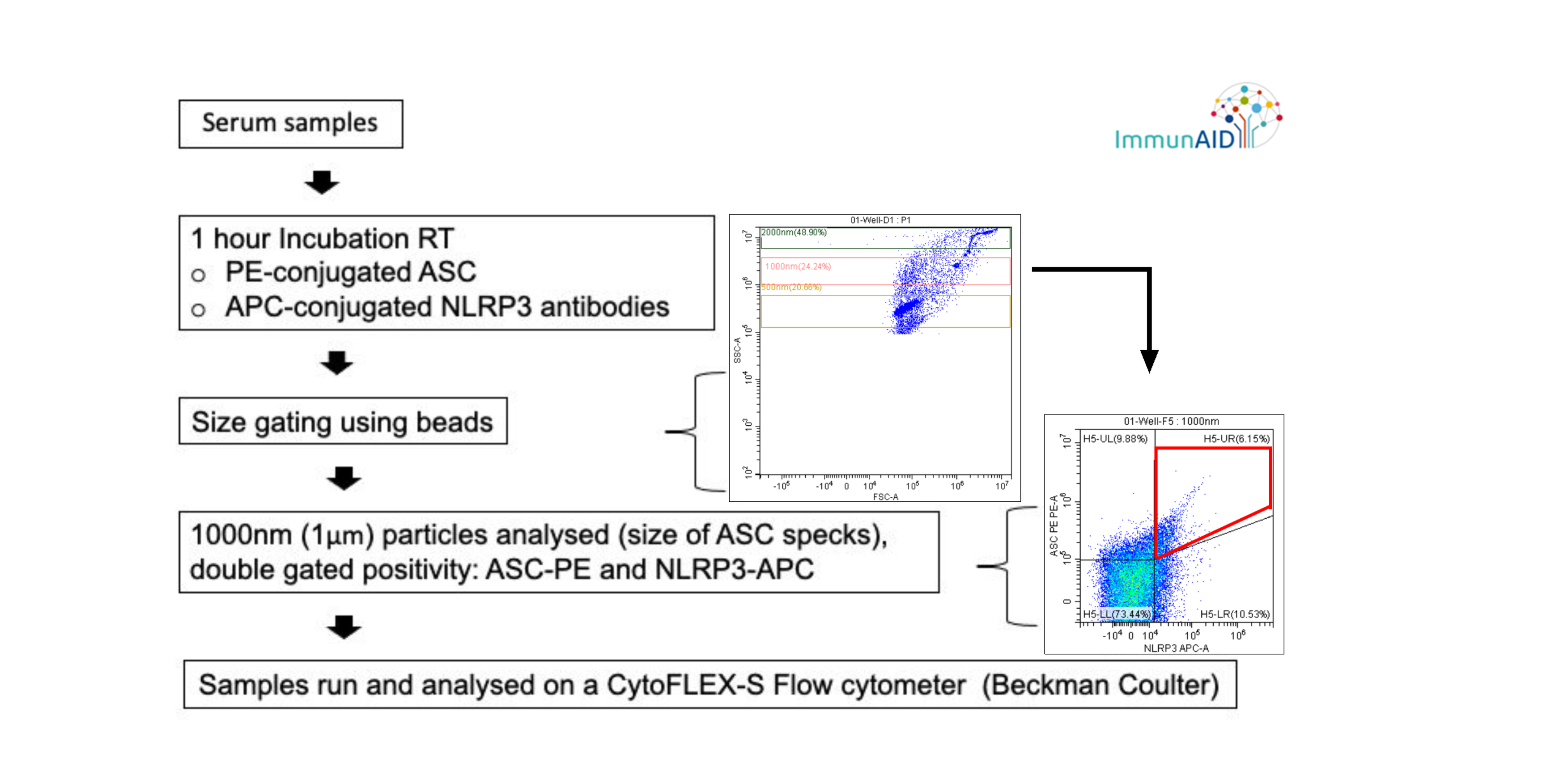

Supplement: Supplementary file 5 — Supplemental Figure S1. ASC/NLRP3 gating strategy. Schematic diagram illustrating the methodology behind the ASC/NLRP3 Specks gating. The area highlighted in red reflects ASC‐PE and NLRP3‐APC double positive events. [file ART-77-582-s007.tiff]

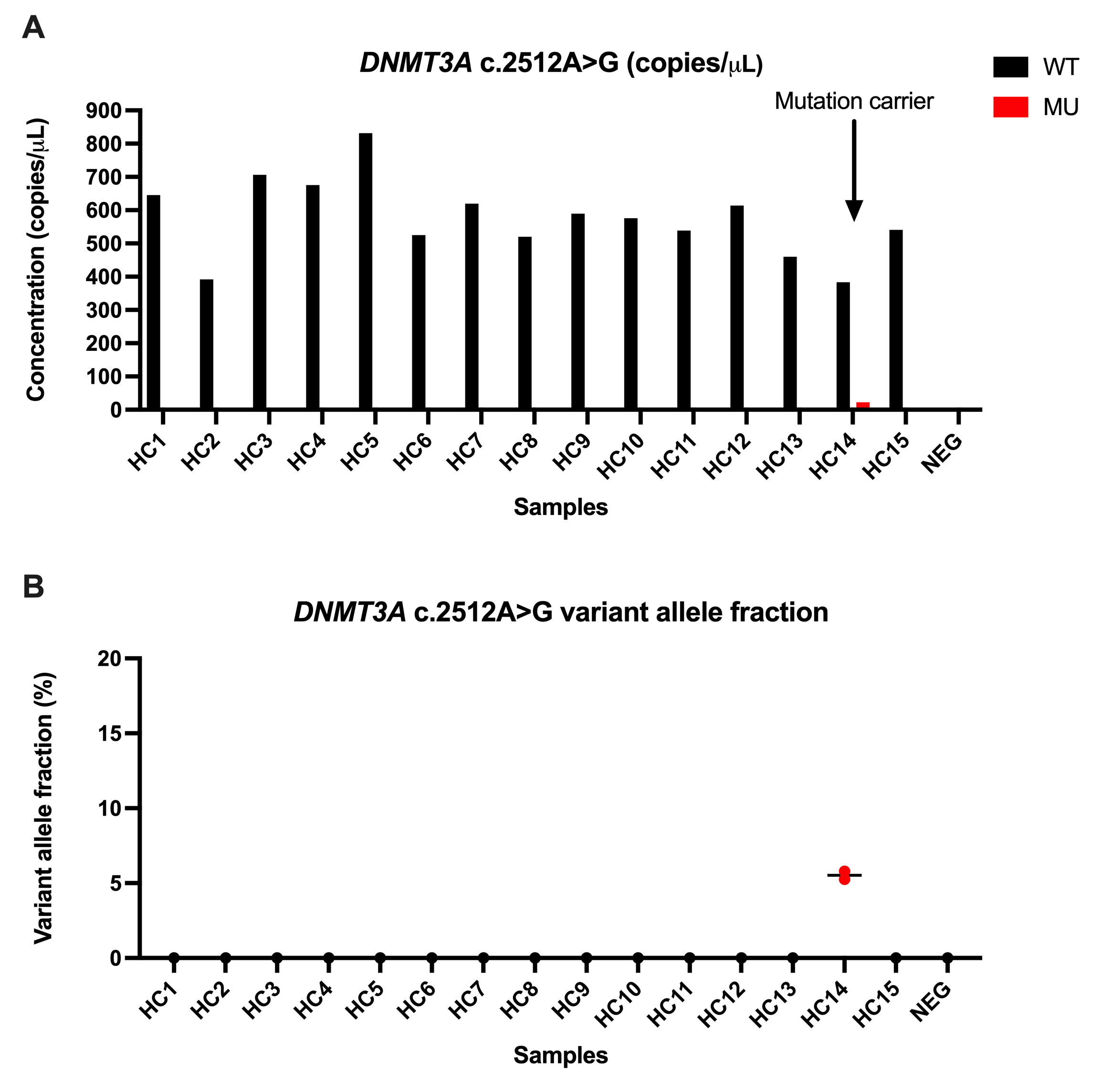

Supplement: Supplementary file 6 — Supplemental Figure S2. Digital PCR (dPCR) validation of DNMT3A c.2512A>G. A putative somatic variant DNMT3A c.2512A>G was identified in a healthy control at a read depth of 69× (WT 61 reads/ MU 8 reads) and we validated this variant to define our lower limit of somatic variant detection. (A) The mean copy number of wild‐type (DNMT3A c.2512A) and variant nucleotide (DNMT3A c.2512G) in each healthy control sample (n=15). The variant was detected in HC14 through exome sequencing and has also been detected through dPCR. (B) Variant allele fractions (%) of DNMT3A c.2512G detected in each sample. Each assay was run in duplicate for all individuals. [file ART-77-582-s005.tiff]

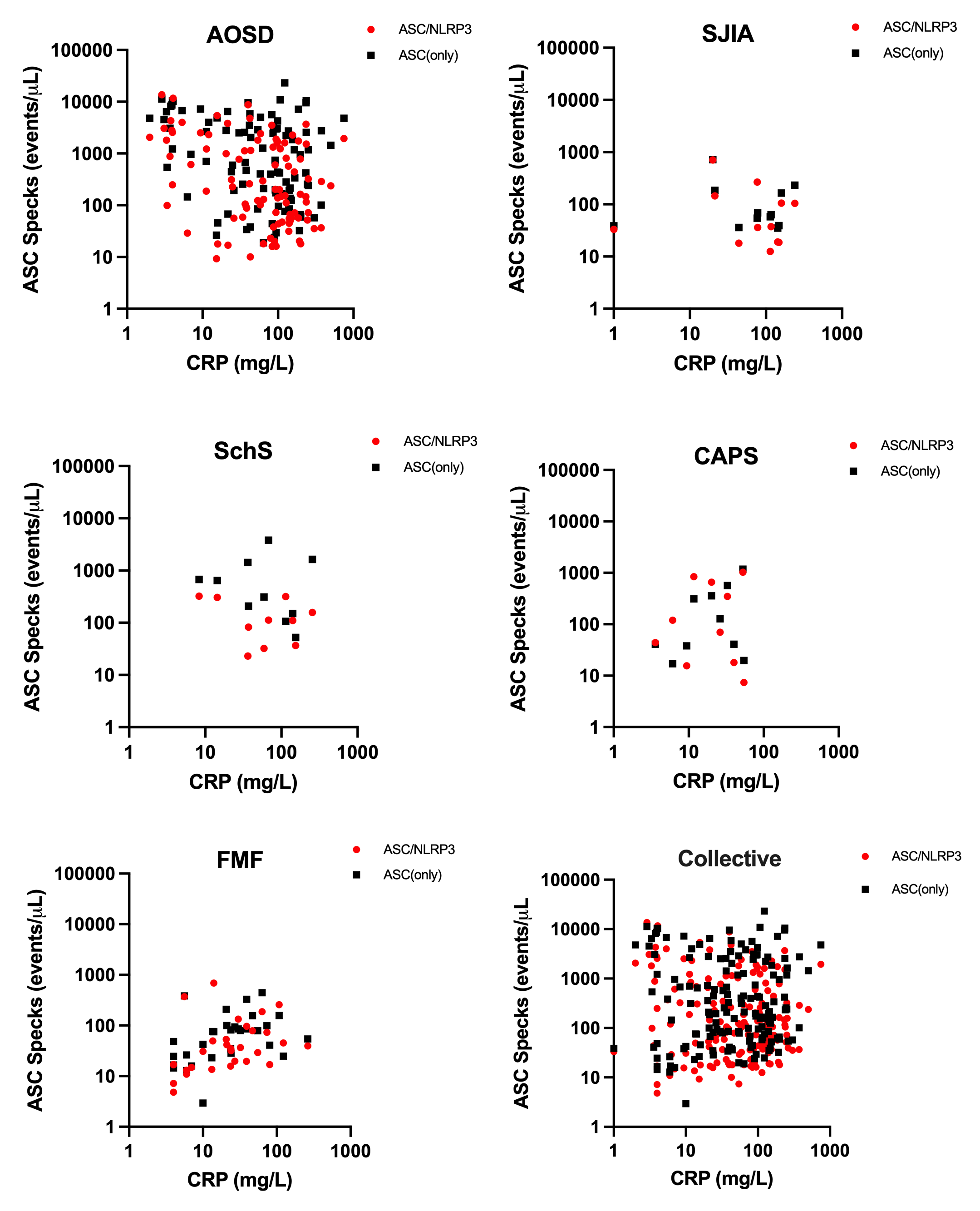

Supplement: Supplementary file 7 — Supplemental Figure S3. Scatter plots comparing CRP (mg/L) against ASC specks (ASC/NLRP3: Red; ASC(only): Black; events/⎧L) for adult onset Still's disease (AOSD, n=106), Systemic juvenile idiopathic arthritis (SJIA, n=12), Schnitzler syndrome (SchS, n=10), Cryopyrin‐associated periodic syndrome (CAPS, n=11), familial Mediterranean fever (FMF, n=31), and the collective disease cohort. No statistically significant correlation was identified between CRP and ASC specks in the combined cohort nor individual disease. [file ART-77-582-s003.tiff]

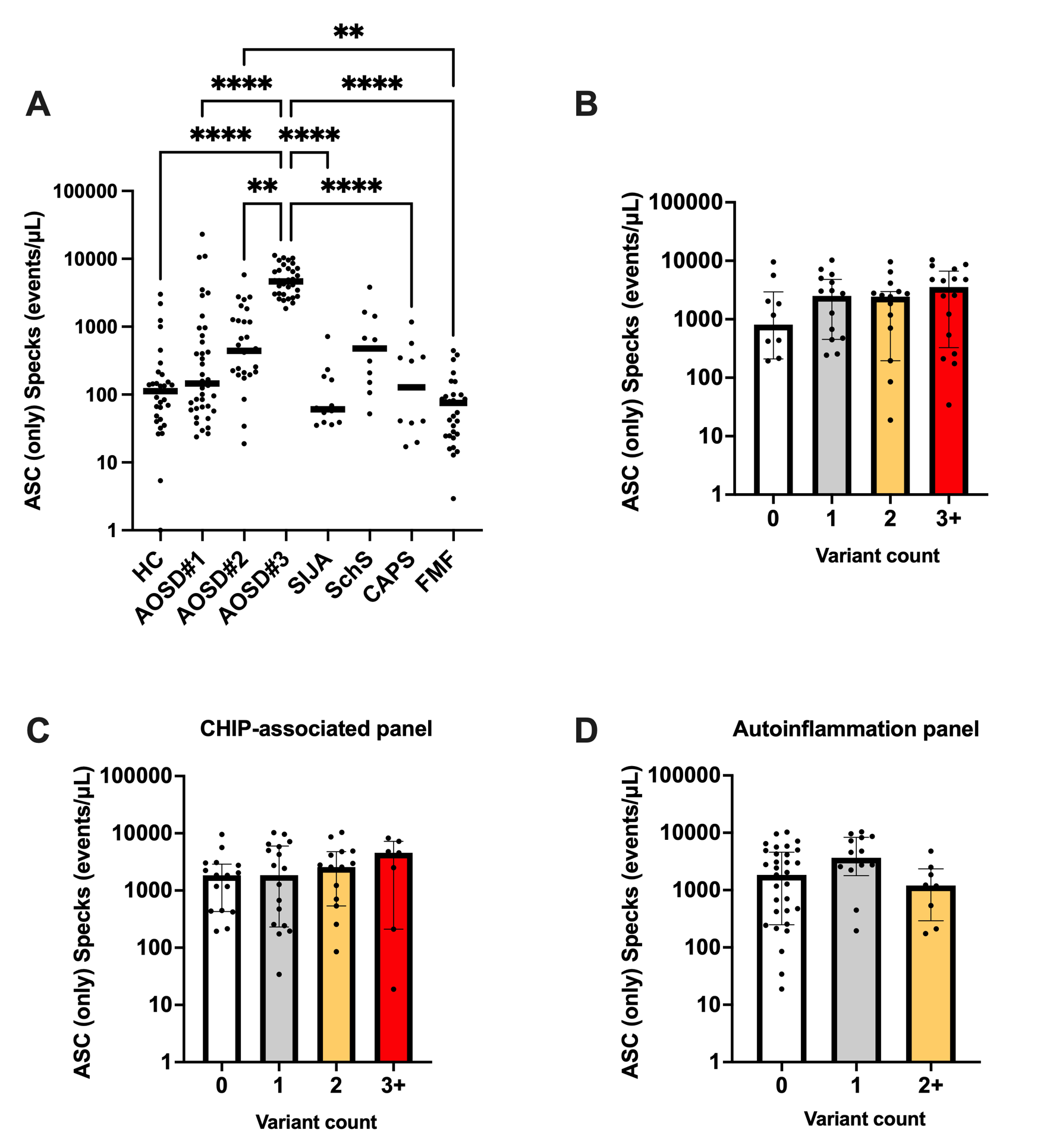

Supplement: Supplementary file 8 — Supplemental Figure S4. (A) ASC(only) specks in healthy controls (HC, n= 30) were compared to AOSD cohort subsets (AOSD#1 (n=39), AOSD#2 (n=30) and AOSD#3 (n=34)), SIJA (n=12), SchS (n=10), CAPS (n=11) and FMF (n=31) cohorts. The weighted bars represent median values. Statistically significant pairwise comparisons are annotated with * =p<0.05; ** =p<0.01; *** =p<0.001; **** =p<0.0001. Bar charts (median, IQR) illustrating ASC(only) specks between AOSD cases carrying 0, 1, 2, and 3+ variants (germline and/or somatic) within (B) any gene from our pre‐selected panels; (C) CHIP‐associated panel only and (D) autoinflammation panel only. [file ART-77-582-s001.tiff]

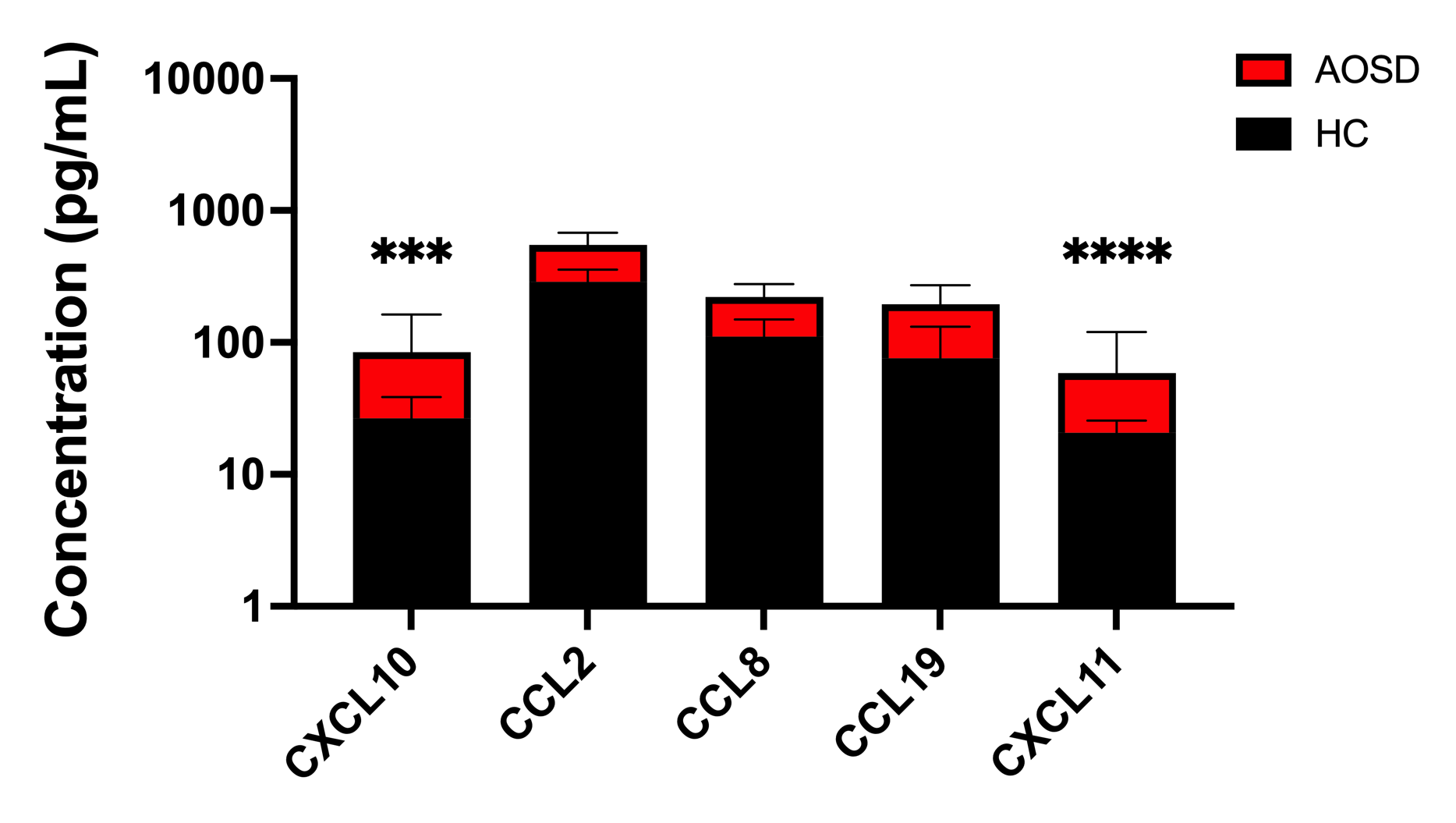

Supplement: Supplementary file 9 — Supplemental Figure S5. Chemokine concentrations for Type‐I Interferon scoring. The Type‐I Interferon score was investigated using a custom Luminex Discovery Assay assessing chemokines: CXCL10, CCL2, CCL8, CCL19 and CXCL11. The raw data (pg/mL) from the HC (black) and AOSD (red) cohorts are displayed on stacked barcharts presenting median (IQR). Pairwise comparisons were assessed by Mann Whitney tests and statistical significance is denoted by asterisks. * =p<0.05; ** =p<0.01; *** =p<0.001; **** =p<0.0001. [file ART-77-582-s012.tiff]

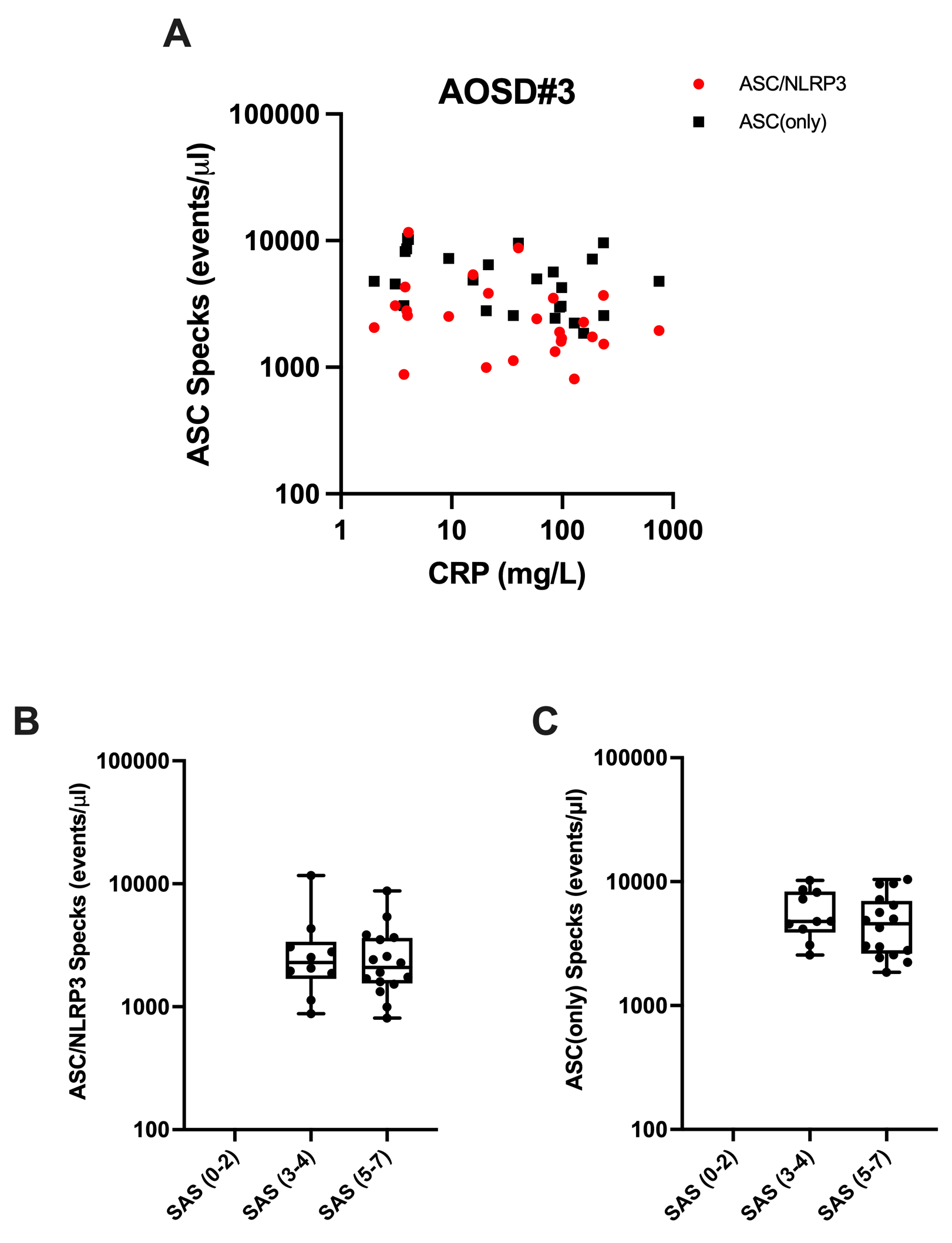

Supplement: Supplementary file 10 — Supplemental Figure S6. Comparisons between ASC/NLRP3 and ASC(only) specks against (A) CRP (mg/L) and (B;C) SAS in the treatment refractory AOSD cohort (AOSD#3, n=30). No significant correlations were identified between these variables. [file ART-77-582-s006.tiff]
