# Supplementary material for: Characterization of Genetic Landscape and Novel Inflammatory Biomarkers in Patients With Adult‐Onset Still's Disease
Source: Arthritis Rheumatol. 2024 Dec 16;77(5):582–95. doi: 10.1002/art.43054 (PMC12039473; doi:10.1002/art.43054)
Supplement: Supplementary file 11 — Supplemental Table S1. Cohort demographics and treatment history. [file ART-77-582-s002.pdf]

Supplemental Table S1 – Sample Demographics

Adult-Onset Still's Disease Cohorts

Adult-Onset Still's Disease Cohort 1 (AOSD#1, n= 39)

Demographics			Selected symptoms			Labs	
Age	Sex	SAS(At sampling)	Fever	Arthralgia	Arthritis >=2 joints	Feritin ug/l	CRP/hsCRP
29	F	5	Yes	Yes	No	292	43
35	F	4	Yes	No	No	4220	14.63/ 141
57	M	4	Yes	No	No	1755	107
41	M	4	Yes	No	No	46132	500
39	M	7	Yes	Yes	Yes	1027	87
19	F	4	Yes	No	Yes	191	80
33	F	6	Yes	Yes	No	7698	234
23	F	5	Yes	Yes	No	87	25
23	F	5	Yes	No	Yes	16369	145.6
30	F	4	Yes	Yes	No	3000	128
30	F	6	Yes	Yes	No	14327	84
27	M	6	Yes	Yes	No	3041	198
29	F	N/A	Yes	No	No	Not performed	123
29	F	5	Yes	Yes	Yes	200	101
37	M	5	Yes	Yes	No	214	192
32	F	4	Yes	No	No	1737	6.3
48	F	5	Yes	Yes	No	51	15.3
27	M	4	Yes	No	No	2706	150
35	F	4	Yes	No	No	3226	167
44	M	4	Yes	No	No	1939	113
35	M	6	Yes	Yes	No	2741	42
63	F	5	Yes	Yes	No	247	200
63	F	7	Yes	Yes	Yes	1382	21.8
30	F	6	Yes	Yes	No	3041	58.4
35	F	6	Yes	Yes	No	3756	94.4
66	M	6	Yes	Yes	No	19803	123.3
25	F	4	No	Yes	Yes	338	91.5
32	F	N/A	Yes	No	No	Not performed	15.9
38	F	4	Yes	No	No	10277	301.5
51	M	6	Yes	Yes	No	1050	7
16	F	6	Yes	Yes	No	3692	53.7
31	F	N/A	Yes	No	No	Not performed	374.4
25	M	N/A	Yes	Yes	No	210	237
50	M	5	Yes	Yes	No	923	92
42	F	6	Yes	No	Yes	129	54
22	F	4	Yes	Yes	No	148	90
30	M	5	Yes	No	No	457	187
36	M	4	Yes	No	Yes	1232	112
23	M	5	Yes	No	No	3894	197

Adult-Onset Still's Disease Cohort 2 (AOSD#2, n= 30)

Demographics			Selected symptoms			Labs	
Age	Sex	SAS(At sampling)	Fever	Arthralgia	Arthritis >=2 joints	Feritin ug/l	CRP/hsCRP
28	F	1	No	No	No	2396	4
58	F	2	No	No	No	30896	30.4
66	F	0	No	No	No	2073	375.6
40	M	1	No	No	No	509	252
22	M	1	No	No	No	1038	165
59	M	2	No	No	No	2923	0.4
58	F	4	No	Yes	No	1077	99.5
21	F	1	No	No	No	252	140.8
24	F	2	No	No	No	70000	26.1
28	M	2	No	No	No	1424	11.2
45	F	0	No	No	No	21160	93.7
51	M	0	No	No	No	351	62.9
21	M	0	No	No	No	293	42.5
30	M	4	No	Yes	No	1851	105.3
19	M	2	Yes	No	No	329	43.6
72	F	1	No	No	No	387	245.5
62	M	4	No	Yes	No	262	64.3
56	F	0	No	No	No	4467	3.4
19	M	0	No	No	No	1984	139
39	F	2	No	Yes	No	2135	37.8
43	M	4	No	Yes	No	1273	36.8
40	F	2	No	No	No	2224	144.5
55	F	3	No	Yes	No	642	71.5
29	F	2	No	Yes	No	5968	137.5
36	F	4	No	Yes	Yes	538	34.1
26	F	3	No	Yes	No	39.1	24.2
27	F	5	Yes	Yes	No	495	25.2
56	F	2	Yes	No	No	134600	252.3
28	F	3	Yes	Yes	No	16.3	38.5
26	F	2	No	Yes	No	984	64.2

Samples highlighted grey were sent for whole exome sequencing.

Adult-Onset Still's Disease Cohort 3 (AOSD#3, n= 37)

Demographics			Selected symptoms			Labs	
Age	Sex	SAS(At sampling)	Fever	Arthralgia	Arthritis >=2 joints	Feritin ug/l	CRP/hsCRP
35	M	7	No	Yes	Yes	Not known	235.9
65	F	4	No	Yes	Yes	Not known	3.91
51	F	5	No	Yes	Yes	Not known	21.4
29	M	7	No	Yes	Yes	Not known	94.2
76	M	6	No	Yes	Yes	Not known	20.5
57	F	5	No	Yes	Yes	Not known	40.1
46	F	7	No	Yes	Yes	Not known	83
41	F	4	No	Yes	Yes	Not known	0.75
24	M	7	No	Yes	Yes	Not known	85.8
24	F	4	No	Yes	Yes	Not known	4.06
44	F	4	No	Yes	Yes	Not known	<2.0
62	M	5	No	Yes	Yes	Not known	58.9
35	M	5	No	Yes	Yes	Not known	155.9
22	M	4	No	Yes	Yes	Not known	748
55	F	6	No	Yes	Yes	Not known	38
47	F	4	No	Yes	Yes	Not known	3.7
61	F	6	No	Yes	Yes	Not known	128.6
27	F	6	No	Yes	Yes	Not known	98.6
50	M	N/A	No	Yes	Yes	Not known	N/A
53	F	N/A	No	Yes	Yes	Not known	N/A
40	M	7	No	Yes	Yes	Not known	4
24	F	3	No	Yes	Yes	Not known	3.8
32	F	4	No	Yes	Yes	Not known	9.4
28	M	7	No	Yes	Yes	Not known	97.6
23	M	5	No	Yes	Yes	Not known	236.5
42	F	4	No	Yes	Yes	Not known	36.1
70	F	4	No	Yes	Yes	Not known	3.1
44	F	6	No	Yes	Yes	Not known	15.6
52	F	7	No	Yes	Yes	Not known	186.9
48	F	N/A	No	Yes	Yes	Not known	N/A
36	F	7	No	Yes	Yes	Not known	11.27
34	F	4	No	Yes	Yes	Not known	0.35
34	F	4	No	Yes	Yes	Not known	5.35
52	M	4	No	Yes	Yes	Not known	0.05
28	F	4	No	Yes	Yes	Not known	2.89
39	F	4	No	Yes	Yes	Not known	3.33
37	F	7	No	Yes	Yes	Not known	12.1

Samples highlighted grey were sent for whole exome sequencing.

Additional Disease Cohorts

Systemic Juvenile Idiopathic Arthritis Cohort (SJIA, n= 12)

Demographics			Selected symptoms			Labs	
Age	Sex	SAS(At sampling)	Fever	Arthralgia	Arthritis >=2 joints	Feritin ug/l	CRP/hsCRP
11	Female	-	-	-	-	-	240
15	Male	-	-	-	-	-	114
10	Female	-	-	-	-	-	44
8	Male	-	-	-	-	-	21.3
14	Male	-	-	-	-	-	1
6	Male	-	-	-	-	-	76.9
11	Female	-	-	-	-	-	160
9	-	-	-	-	-	-	117
12	Male	-	-	-	-	-	148
11	Male	-	-	-	-	-	143
9	Male	-	-	-	-	-	20
4	Female	-	-	-	-	-	77.8

Schnitzler Syndrome cohort (SCHN, n= 10)

Demographics			Selected symptoms			Labs	
Age	Sex	SAS(At sampling)	Fever	Arthralgia	Arthritis >=2 joints	Feritin ug/l	CRP/hsCRP
64	Male	-	-	-	-	-	59
87	Female	-	-	-	-	-	154
68	Male	-	-	-	-	-	140.5
79	Female	-	-	-	-	-	36.3
48	Female	-	-	-	-	-	255
48	Male	-	-	-	-	-	68
66	Female	-	-	-	-	-	114
72	Male	-	-	-	-	-	37
58	Female	-	-	-	-	-	8.3
76	Female	-	-	-	-	-	14.4

Cryopyrin-associated autoinflammatory syndromes (CAPS, n= 11)

Demographics			Selected symptoms			Labs	
Age	Sex	SAS(At sampling)	Fever	Arthralgia	Arthritis >=2 joints	Feritin ug/l	CRP/hsCRP
30	Male	-	-	-	-	-	52.7
56	Female	-	-	-	-	-	54.6
44	Male	-	-	-	-	-	9.4
68	Female	-	-	-	-	-	40.1
41	Male	-	-	-	-	-	32.9
16	Male	-	-	-	-	-	3.6
11	Female	-	-	-	-	-	11.7
11	Male	-	-	-	-	-	20.1

21	Male	-	-	-	-	-	26.2
35	-	-	-	-	-	-	-
30	Female	-	-	-	-	-	6.1

Familial Mediterranean Fever (FMF, n= 31)

Demographics			Selected symptoms			Labs	
Age	Sex	SAS(At sampling)	Fever	Arthralgia	Arthritis >=2 joints	Feritin ug/l	CRP/hsCRP
70	Male	-	-	-	-	-	30
46	Male	-	-	-	-	-	38.9
42	Female	-	-	-	-	-	264
40	Female	-	-	-	-	-	13.2
68	Female	-	-	-	-	-	21
23	Female	-	-	-	-	-	80.3
43	Female	-	-	-	-	-	5.6
21	Female	-	-	-	-	-	46.8
17	Female	-	-	-	-	-	32.3
64	Female	-	-	-	-	-	23.8
76	Female	-	-	-	-	-	63.1
39	Female	-	-	-	-	-	23.7
22	-	-	-	-	-	-	14
24	Female	-	-	-	-	-	4
23	Female	-	-	-	-	-	4
72	Male	-	-	-	-	-	4
65	Female	-	-	-	-	-	4
23	Female	-	-	-	-	-	6
12	Male	-	-	-	-	-	27
11	Male	-	-	-	-	-	55
6	Male	-	-	-	-	-	13.6
16	Female	-	-	-	-	-	39
4	Male	-	-	-	-	-	10
33	Male	-	-	-	-	-	24
38	Female	-	-	-	-	-	20.7
25	Female	-	-	-	-	-	6
74	Male	-	-	-	-	-	73.4
30	Female	-	-	-	-	-	108
26	Female	-	-	-	-	-	10
34	Female	-	-	-	-	-	7
20	Male	-	-	-	-	-	123

Healthy Control Cohorts

Healthy Control Cohort 1 (HC#1, n= 49, WES)

Demographics			Selected symptoms			Labs	
Age	Sex	SAS(At sampling)	Fever	Arthralgia	Arthritis >=2 joints	Feritin ug/l	CRP/hsCRP
60	F	-	-	-	-	-	-
53	F	-	-	-	-	-	-
64	F	-	-	-	-	-	-
21	M	-	-	-	-	-	-
63	F	-	-	-	-	-	-
26	F	-	-	-	-	-	-
22	F	-	-	-	-	-	-
57	M	-	-	-	-	-	-
76	F	-	-	-	-	-	-
46	F	-	-	-	-	-	-
64	M	-	-	-	-	-	-
30	M	-	-	-	-	-	-
28	F	-	-	-	-	-	-
62	F	-	-	-	-	-	-
43	F	-	-	-	-	-	-
36	F	-	-	-	-	-	-
33	F	-	-	-	-	-	-
40	F	-	-	-	-	-	-
26	F	-	-	-	-	-	-
39	M	-	-	-	-	-	-
78	F	-	-	-	-	-	-
33	F	-	-	-	-	-	-
75	M	-	-	-	-	-	-
29	F	-	-	-	-	-	-
62	M	-	-	-	-	-	-
29	M	-	-	-	-	-	-
36	M	-	-	-	-	-	-
63	M	-	-	-	-	-	-
48	M	-	-	-	-	-	-
46	M	-	-	-	-	-	-
59	F	-	-	-	-	-	-
27	M	-	-	-	-	-	-
69	F	-	-	-	-	-	-
23	M	-	-	-	-	-	-
30	F	-	-	-	-	-	-
42	F	-	-	-	-	-	-
58	M	-	-	-	-	-	-
59	F	-	-	-	-	-	-
23	F	-	-	-	-	-	-
47	F	-	-	-	-	-	-
79	M	-	-	-	-	-	-
69	M	-	-	-	-	-	-
80	M	-	-	-	-	-	-

76	M	-	-	-	-	-	-
76	M	-	-	-	-	-	-
80	M	-	-	-	-	-	-
78	M	-	-	-	-	-	-
77	M	-	-	-	-	-	-
78	M	-	-	-	-	-	-

Samples highlighted grey were sent for whole exome sequencing.

Healthy Control Cohort 2 (HC#2, n= 10, RNAseq)

Demographics			Selected symptoms			Labs	
Age	Sex	SAS(At sampling)	Fever	Arthralgia	Arthritis >=2 joints	Feritin ug/l	CRP/hsCRP
-	Female	-	-	-	-	-	-
-	Male	-	-	-	-	-	-
-	Female	-	-	-	-	-	-
-	Female	-	-	-	-	-	-
-	Female	-	-	-	-	-	-
-	Female	-	-	-	-	-	-
-	Female	-	-	-	-	-	-
-	Female	-	-	-	-	-	-
-	Male	-	-	-	-	-	-
-	Male	-	-	-	-	-	-

Healthy Control Cohort 3 (HC#3, n= 32, Functional assays)

Demographics			Selected symptoms			Labs	
Age	Sex	SAS(At sampling)	Fever	Arthralgia	Arthritis >=2 joints	Feritin ug/l	CRP/hsCRP
53	Female	-	-	-	-	-	-
45	Male	-	-	-	-	-	-
50	Male	-	-	-	-	-	-
24	Male	-	-	-	-	-	-
39	Female	-	-	-	-	-	-
40	Male	-	-	-	-	-	-
55	Female	-	-	-	-	-	-
31	Male	-	-	-	-	-	-
30	Female	-	-	-	-	-	-
34	Female	-	-	-	-	-	-
37	Male	-	-	-	-	-	-
37	Female	-	-	-	-	-	-
11	Female	-	-	-	-	-	-
27	Female	-	-	-	-	-	-
27	Female	-	-	-	-	-	-
26	Male	-	-	-	-	-	-

38	Male	-	-	-	-	-	-
49	Female	-	-	-	-	-	-
59	Male	-	-	-	-	-	-
26	Male	-	-	-	-	-	-
39	Male	-	-	-	-	-	-
44	Female	-	-	-	-	-	-
29	Male	-	-	-	-	-	-
28	Male	-	-	-	-	-	-
38	Male	-	-	-	-	-	-
47	Male	-	-	-	-	-	-
27	Male	-	-	-	-	-	-
29	Female	-	-	-	-	-	-
54	Female	-	-	-	-	-	-
15	Male	-	-	-	-	-	-
