# Supplementary material for: Characterization of Genetic Landscape and Novel Inflammatory Biomarkers in Patients With Adult‐Onset Still's Disease
Source: Arthritis Rheumatol. 2024 Dec 16;77(5):582–95. doi: 10.1002/art.43054 (PMC12039473; doi:10.1002/art.43054)
Supplement: Supplementary file 12 — Supplemental Table S2. Pre‐selected gene panels. [file ART-77-582-s010.pdf]

CHIP-associated panel (87 genes)		Autoinflammatory panel (38 genes)	Type I Interferonopathies panel (14 genes)
ABL1	*LAMB4*	*ADA2*	*ACP5*
ANKRD26	*LUC7L2*	*ALPK1*	*ADAR*
ARAF	*MAP2K1*	*AP1S3*	*DDX58*
ASXL1	*MECOM*	*CARD14*	*DNAse2*
ASXL2	*MPL*	*CDC42*	*IFIH1*
ATRX	*MYB*	*CEBPE*	*ISG15*
BCOR	*NCOR2*	*IL1RN*	*POLA1*
BCORL1	*NF1*	*IL36RN*	*RNASEH2A*
BRAF	*NFE2*	*LACC1*	*RNASEH2B*
CALR	*NPM1*	*LPIN2*	*RNASEH2C*
CBFB	*NRAS*	*MEFV*	*SAMHD1*
CBL	*PDGFRA*	*MVK*	*SKIV2L*
CDKN1B	*PDGFRB*	*NCSTN*	*TREX-1*
CDKN2A	*PDS5B*	*NLRC4*	*UDP18*
CEBPA	*PHF6*	*NLRP1*	
CHEK2	*PIGA*	*NLRP12*	
CREBBP	*PPM1D*	*NLRP3*	
CSF3R	*PRPF8*	*NOD2*	
CTCF	*PTEN*	*OTULIN*	
CUX1	*PTPN11*	*PLCG2*	
DDX41	*RAD21*	*POMP*	
DNMT3A	*RAD51*	*PSMA3*	
EGFR	*RUNX1*	*PSMB10*	
EP300	*SETBP1*	*PSMB4*	
ERBB3	*SETD2*	*PSMB8*	
ETNK1	*SF1*	*PSMB9*	
ETV6	*SF3B1*	*PSMG2*	
EZH2	*SH2B3*	*PSTPIP1*	
FAT4	*SMC1A*	*RBCK1*	
FLT3	*SMC3*	*RELA*	
GATA1	*SRSF2*	*RIPK1*	
GATA2	*STAG2*	*SLC29A3*	
GNAS	*SUZ12*	*TMEM173*	
GNB1	*TET2*	*TNFAIP3*	
GPRC5A	*THRAP3*	*TNFRSF1A*	
HRAS	*TP53*	*TRAP1*	
IDH1	*U2AF2*	*TRNT1*	
IDH2	*UBA1*	*WDR1*	
IKZF1	*WT1*		
IRF1	*ZRSR2*		
JAK2			
JAK3			
KDM6A			
KIT			
KMT2A			
KMT2D			
KRAS			
