# Supplementary material for: Characterization of Genetic Landscape and Novel Inflammatory Biomarkers in Patients With Adult‐Onset Still's Disease
Source: Arthritis Rheumatol. 2024 Dec 16;77(5):582–95. doi: 10.1002/art.43054 (PMC12039473; doi:10.1002/art.43054)
Supplement: Supplementary file 13 — Supplemental Table S3. Whole exome sequencing metrics. [file ART-77-582-s016.pdf]

Supplemental Table S3. Whole exome sequencing metrics

Adult-onset Still's Disease Cohort:

Sample	Unique pairs (%)	Unique pairs	Duplication (%)	Mean read depth	% bases above 50 reads	% bases above 100 reads
AOSD_S01	74.80%	91006718	25.20%	162.16	85.8	66
AOSD_S02	77.50%	84232702	22.50%	152.37	84.6	63.5
AOSD_S03	81.00%	55378686	19.00%	101.23	74.8	42.4
AOSD_S04	75.00%	97257368	25.00%	178.37	87.1	69.6
AOSD_S05	79.20%	71300404	20.80%	130.27	81.4	55.6
AOSD_S06	79.10%	87294646	20.90%	156.25	85	63.8
AOSD_S07	72.60%	57096784	27.40%	106.3	71.1	42.6
AOSD_S08	75.50%	92122676	24.50%	167.43	86	67
AOSD_S09	75.50%	62761214	24.50%	116.07	76.3	47.2
AOSD_S10	78.50%	69521594	21.50%	127.92	79.8	53.4
AOSD_S11	76.80%	101769324	23.20%	185.02	87.7	71.3
AOSD_S12	77.20%	90999044	22.80%	164.34	86.2	66.8
AOSD_S13	76.10%	88037918	23.90%	157.96	85.2	64.3
AOSD_S14	79.70%	64598204	20.30%	115.4	78	49
AOSD_S15	76.80%	81248308	23.20%	144.99	83.9	61.1
AOSD_S16	77.00%	95015962	23.00%	171.97	86.6	68.4
AOSD_S17	76.00%	108268436	24.00%	195.55	88.2	72.6
AOSD_S18	82.50%	66752088	17.50%	119.49	79.3	51
AOSD_S19	77.30%	108760650	22.70%	194.77	88.3	72.6
AOSD_S20	79.30%	76505468	20.70%	143.67	82.2	58.3
AOSD_S21	77.60%	91967668	22.40%	167.64	86	66.6
AOSD_S22	77.20%	91848824	22.80%	164.58	85.9	66.4
AOSD_S23	78.00%	84115368	22.00%	148.42	84.6	63.1
AOSD_S24	78.30%	91811458	21.70%	162.4	85.9	66.2
AOSD_S25	78.40%	73883468	21.60%	132.72	81.8	56.4
AOSD_S26	82.80%	80001466	17.20%	143.39	81.9	57.8
AOSD_S27	75.80%	176219964	24.20%	317.69	92.4	85.1
AOSD_S28	78.00%	171141278	22.00%	310.31	92.2	84.5
AOSD_S29	76.80%	78136154	23.20%	140.4	82.9	58.9

AOSD_S30	73.20%	104006200	26.80%	185.24	88.1	72.3
AOSD_S31	74.60%	65594736	25.40%	124.63	80.1	54.2
AOSD_S32	73.80%	61106662	26.20%	116.91	78.1	50.4
AOSD_S33	71.20%	69633050	28.80%	132.06	81.1	56.8
AOSD_S34	64.80%	94778856	35.20%	176.24	86.5	70.1
AOSD_S35	72.10%	84087812	27.90%	157.92	84.5	64.9
AOSD_S36	71.50%	68571598	28.50%	130.93	81.1	56.3
AOSD_S37	69.80%	70172258	30.20%	132.16	81.5	57.4
AOSD_S38	71.10%	60525656	28.90%	113.59	78	49.2
AOSD_S39	69.60%	77946742	30.40%	147.76	83.4	62.3
AOSD_S40	67.20%	81789016	32.80%	152.75	84.4	64.2
AOSD_S41	72.70%	73787688	27.30%	140.31	82.2	59.5
AOSD_S42	73.40%	72270850	26.60%	136.24	81.8	58.2
AOSD_S43	71.90%	64026458	28.10%	121.47	79.2	52.4
AOSD_S44	69.00%	58037630	31.00%	106.49	76.8	46.6
AOSD_S45	71.60%	54459548	28.40%	99.55	74.7	42.6
AOSD_S46	73.50%	63402616	26.50%	119.18	79.1	51.8
AOSD_S47	72.60%	63150168	27.40%	113.74	79	50.6
AOSD_S48	72.70%	55315100	27.30%	100.26	74.6	43
AOSD_S49	68.80%	66317760	31.20%	120.74	80.2	53.7
AOSD_S50	71.60%	58620080	28.40%	108.44	77.4	48
AOSD_S51	69.70%	53240854	30.30%	95.86	73.3	40.4
AOSD_S52	71.10%	67473798	28.90%	124.48	81	55.8
AOSD_S53	69.30%	70413476	30.70%	127.11	81.5	56.6
AOSD_S54	71.70%	61840212	28.30%	113.67	79	50.7
AOSD_S55	73.20%	56114172	26.80%	101.46	75.8	44.3
AOSD_S56	70.60%	66794566	29.40%	122.5	80.6	54.7
AOSD_S57	72.60%	58445342	27.40%	108.19	77.5	48
AOSD_S58	69.20%	62323522	30.80%	114.46	79.1	51.2
AOSD_S59	72.70%	55627354	27.30%	99.17	75.3	42.8
AOSD_S60	70.80%	57805402	29.20%	105.71	76.9	46.6
			**Mean Depth**	**142.14**		

Healthy Controls Cohort:

Sample	Unique pairs (%)	Unique pairs	Duplication (%)	Mean read depth	% bases above 50 reads	% bases above 100 reads
HC_S01	86.50%	33264696	13.50%	55.75	58.4	3.4
HC_S02	85.70%	36546580	14.30%	60.85	66.9	5.9
HC_S03	85.60%	35308398	14.40%	58.65	63.4	4.6
HC_S04	87.10%	32362250	12.90%	53.24	53.1	2.9
HC_S05	84.40%	39072408	15.60%	65.11	73.9	8
HC_S06	84.90%	40317666	15.10%	67.21	75.8	10
HC_S07	85.30%	36363742	14.70%	60.5	66.4	5.7
HC_S08	84.40%	36005336	15.60%	60.55	65.8	6.2
HC_S09	86.60%	27834822	13.40%	48.19	42.1	1.9
HC_S10	85.20%	34083828	14.80%	58.78	61.5	6.1
HC_S11	85.70%	31207330	14.30%	53.84	53.1	3.8
HC_S12	84.20%	27182498	15.80%	46.44	38.6	1.8
HC_S13	84.00%	35430782	16.00%	60.18	63.6	6.9
HC_S14	85.40%	33626334	14.60%	57.6	59.6	5.4
HC_S15	85.00%	28696740	15.00%	49.52	44.6	2.4
HC_S16	87.40%	22611200	12.60%	39.3	24.4	0.7
HC_S17	85.50%	37250088	14.50%	63.17	67.2	9.5
HC_S18	85.00%	37678790	15.00%	63.72	68.9	9.2
HC_S19	86.30%	38692578	13.70%	65.07	70.9	10
HC_S20	86.10%	31920712	13.90%	54.02	53.3	4
HC_S21	84.80%	35719164	15.20%	60.02	63	7.2
HC_S22	85.00%	30715552	15.00%	51.77	48.8	3.1
HC_S23	84.90%	28467132	15.10%	48.03	41.6	2.1
HC_S24	85.20%	35304500	14.80%	59.67	63.1	6.5
HC_S25	80.90%	29921496	19.10%	50.99	47.1	3.6
HC_S26	86.30%	35336332	13.70%	56.34	57.8	4.6
HC_S27	85.10%	28738360	14.90%	46.48	38.4	1.6
HC_S28	86.50%	21775014	13.50%	35.29	16.1	0.3
HC_S29	86.80%	25043592	13.20%	40.53	25.7	0.7
HC_S30	86.50%	29780734	13.50%	47.95	41.5	1.8
HC_S31	84.80%	38558950	15.20%	62.38	67.3	8
HC_S32	86.60%	25369474	13.40%	41.04	27.2	0.8
HC_S33	85.20%	22707168	14.80%	34.53	13.1	0.3
HC_S34	86.50%	30427660	13.50%	46.81	39	1.5
HC_S35	85.70%	22691398	14.30%	34.68	13.3	0.3
HC_S36	87.20%	19781394	12.80%	29.82	6.2	0.2
HC_S37	85.90%	31307658	14.10%	47.05	39.6	1.5
HC_S38	85.30%	33579686	14.70%	51.68	49.1	2.4
HC_S39	86.20%	34883240	13.80%	59.88	65.9	5.1
HC_S40	83.90%	35887096	16.10%	55.49	57.5	3.3

HC_S41	86.00%	48875426	14.00%	85.55	88.5	29.5
HC_S42	85.20%	54024554	14.80%	95.44	92.4	41.5
HC_S43	82.80%	53348232	17.20%	93.53	91.8	39.3
HC_S44	86.50%	37842630	13.50%	66.85	74.3	10.4
HC_S45	86.40%	27606412	13.60%	47.41	41.5	1.3
HC_S46	83.60%	22827444	16.40%	40.15	24.5	0.6
HC_S47	86.70%	40233474	13.30%	69.6	79.5	11.2
HC_S48	87.60%	32819864	12.40%	56.03	60.1	3.2
HC_S49	86.90%	16424626	13.10%	27.89	4.3	0.1
			**Mean depth**	**54.79**		
