# Supplementary material for: Characterization of Genetic Landscape and Novel Inflammatory Biomarkers in Patients With Adult‐Onset Still's Disease
Source: Arthritis Rheumatol. 2024 Dec 16;77(5):582–95. doi: 10.1002/art.43054 (PMC12039473; doi:10.1002/art.43054)
Supplement: Supplementary file 14 — Supplemental Table S4. Germline variants identified in cases of adult‐onset Still's disease. An overview of 93 rare (<1% population allele frequency), potentially pathogenic (CADD>20) germline variants were identified in AOSD cases across targeted gene panels associated with CHIP, autoinflammation and type‐I interferonopathy. Genomic coordinates provided for the human reference genome build GRCh38. c.DNA nomenclature provided according to the selected RefSeq transcript. Enrichment analysis was performed for variants found unique to AOSD cases (89/93 variants), the incidence of each variant was compared to their prevalence recorded in the European (Non‐Finnish) population within gnomAD v4.0.0, and statistically significant enrichment is underlined in red (defined by adjusted p‐value <0.000352 after multiple testing correction). The pathogenicity classification from ClinVar and INFEVERS (autoinflammation panel only) are displayed for each variant to aid interpretation. [file ART-77-582-s004.pdf]

SUPPLEMENTAL TABLE S4. Germline variants identified in cases of adult-onset Still's disease

CHIP-ASSOCIATED GENE PANEL

Gene	g.DNA Nomenclature	c.DNA Nomenclature	Protein Nomenclature	RefSeq transcript	dbSNP	Consequence	CADD	Cases (AOSD | HC)	Fisher's Exact (p-value)	ClinVar (Accession)	INFEVERS
ABL1	Chr9:g.130884719C>T	c.2429C>T	p.(P910L)	NM_005157.6	rs2229071	Missense	24.3	2 | 0	0.2297	Benign (VCV000133440.7)	-
ANKRD26	Chr10:g.27040130C>A	c.2210G>T	p.(R737I)	NM_014915.3	rs201688627	Missense	24.0	1 | 0	0.0116	Conflicting Interpretations (VCV000877273.6)	-
ANKRD26	Chr10:g.27035332C>G	c.3118G>C	p.(E1040Q)	NM_014915.3	rs769022656	Missense	23.9	1 | 0	0.0007	-	-
ASXL1	Chr20:g.32433852A>G	c.1654A>G	p.(I552V)	NM_015338.6	rs143952412	Missense	24.9	1 | 0	0.0060	Likely benign (VCV000736660.6)	-
CBFB	Chr16:g.67098735C>G	c.521C>G	p.(P174R)	NM_022845.3	rs971993971	Missense	24.9	1 | 0	0.0004	-	-
CBL	Chr11:g.119298451C>T	c.2345C>T	p.(P782L)	NM_005188.4	rs2229073	Missense	23.9	1 | 0	0.0161	Conflicting Interpretations (VCV000040421.33)	-
CHEK2	Chr22:g.28725031G>A	c.538C>T	p.(R180C)	NM_007194.4	rs77130927	Missense	24.7	1 | 0	0.0992	Conflicting Interpretations (VCV000128081.60)	-
CHEK2	Chr22:g.28711986C>T	c.715G>A	p.(E239K)	NM_007194.4	rs121908702	Missense	22.5	1 | 0	0.0136	Uncertain significance (VCV000005600.35)	-
CREBBP	Chr16:g.3773815G>A	c.2399C>T	p.(P800L)	NM_004380.3	rs371771785	Missense	25.4	1 | 0	0.0006	Uncertain significance (VCV000194217.5)	-
CREBBP	Chr16:g.3728595C>T	c.6452G>A	p.(R2151Q)	NM_004380.3	-	Missense	22.5	1 | 0	0.0009	-	-
CSF3R	Chr1:g.36466446C>T	c.2422G>A	p.(E808K)	NM_000760.4	rs146617729	Missense	23.2	1 | 0	-	Benign/Likely benign (VCV000434833.17)	-
CTCF	Chr16:g.67636779C>T	c.1927C>T	p.(P643S)	NM_006565.4	rs145727304	Missense	25.1	1 | 0	0.5169	Benign/Likely benign (VCV000210800.26)	-
CUX1	Chr7:g.102227604C>G	c.3368C>G	p.(S1123C)	NM_181552.4	rs140448919	Missense	26.5	1 | 0	0.2091	Likely benign (VCV001676073.8)	-
DNMT3A	Chr2:g.25244622C>T	c.1585G>A	p.(D529N)	NM_022552.5	rs962805778	Missense	27.0	1 | 0	0.0003	Likely pathogenic (VCV001708274.1)	-
DNMT3A	Chr2:g.25247647G>A	c.958C>T	p.(R320*)	NM_022552.5	rs778270132	Stop gain	37.0	1 | 0	0.0021	Pathogenic (VCV000955045.2)	-
EP300	Chr22:g.41146776T>G	c.2091T>G	p.(S697R)	NM_001429.4	rs61756764	Missense	26.2	1 | 0	0.5534	Benign/Likely benign (VCV000134035.23)	-
ERBB3	Chr12:g.56101555C>A	c.3529C>A	p.(L1177I)	NM_001982.4	rs55699040	Missense	22.6	1 | 0	0.4482	Conflicting Interpretations (VCV000715634.21)	-
EZH2	Chr7:g.148809150T>C	c.2116A>G	p.(M706V)	NM_004456.5	-	Missense	25.9	1 | 0	0.0001	-	-
EZH2	Chr7:g.148828742T>C	c.623A>G	p.(D208G)	NM_004456.5	rs61753264	Missense	25.3	2 | 0	<0.0001	Uncertain significance (VCV000642027.6)	-
FAT4	Chr4:g.125316935G>T	c.14486G>C	p.(R4829T)	NM_001291303.3	rs368984725	Missense	22.6	1 | 0	0.0005	Uncertain significance (VCV001499643.5)	-
FAT4	Chr4:g.125491302G>C	c.14159C>G	p.(A4720G)	NM_001291303.3	rs76607227	Missense	23.0	1 | 0	0.0281	Likely benign (VCV000787279.7)	-
FAT4	Chr4:g.125490975C>G	c.4432A>G	p.(I1478V)	NM_001291303.3	rs200565115	Missense	22.6	1 | 0	0.0071	Uncertain significance (VCV001028321.3)	-
FAT4	Chr4:g.125320843A>G	c.13802C>T	p.(P4601L)	NM_001291303.3	rs200851934	Missense	22.9	1 | 0	0.0176	Uncertain significance (VCV001417368.5)	-
FAT4	Chr4:g.125490618C>T	c.524G>T	p.(R175L)	NM_001291303.3	rs143534324	Missense	24.7	2 | 0	0.0741	Benign/Likely benign (VCV000391893.34)	-
FAT4	Chr4:g.125449031A>T	c.8021A>T	p.(D2674V)	NM_001291303.3	rs138655269	Missense	24.5	1 | 0	0.3528	Conflicting Interpretations (VCV000420323.12)	-

FAT4	Chr4:g.125398903G>C	c.5295G>C	p.(M1765I)	NM_001291303.3	rs930143534	Missense	21.2	1 | 0	**0.0003**	-	-
GPRC5A	Chr12:g.12908793A>G	c.544A>G	p.(T182A)	NM_003979.4	rs12368599	Missense	23.3	1 | 0	>0.9999	-	-
IDH2	Chr15:g.90087472C>T	c.782G>A	p.(R261H)	NM_002168.4	rs118101777	Missense	29.1	1 | 0	-	Benign/Likely benign (VCV000211177.20)	-
IKZF1	Chr7:g.50400611G>A	c.1544G>A	p.(R515H)	NM_006060.6	rs1159541845	Missense	28.8	1 | 0	0.0006	-	-
JAK2	Chr9:g.5069202C>A	c.1507C>A	p.(P503T)	NM_004972.4	rs1220590246	Missense	22.3	1 | 0	0.0007	-	-
KDM6A	ChrX:g.45060642G>A	c.1363G>A	p.(V455I)	NM_001291415.2	rs1247978055	Missense	20.6	1 | 0	0.0010	Uncertain significance (VCV000432518.3)	-
KIT	Chr4:g.54695731C>T	c.287C>T	p.(T96M)	NM_000222.3	rs1060502564	Missense	23.2	1 | 0	0.0025	Uncertain significance (VCV000409773.10)	-
KMT2A	Chr11:g.118436816G>A	c.304G>A	p.(G102R)	NM_001197104.2	rs781876156	Missense	24.0	1 | 0	0.0019	Uncertain significance (VCV001967465.3)	-
KMT2A	Chr11:g.118491813G>A	c.4889G>A	p.(R1630Q)	NM_001197104.2	rs143317202	Missense	21.3	1 | 0	0.0156	Benign (VCV000724065.5)	-
KMT2D	Chr12:g.49024662C>A	c.15968G>T	p.(R5323L)	NM_003482.4	-	Missense	28.7	1 | 0	**0.0001**	-	-
KMT2D	Chr12:g.49053283G>A	c.878C>T	p.(T293M)	NM_003482.4	rs794727860	Missense	22.0	1 | 0	0.0010	Conflicting Interpretations (VCV000198493.5)	-
KRAS	Chr12:g.25225657C>T	c.407G>A	p.(S136N)	NM_004985.5	rs757816355	Missense	22.2	1 | 0	0.0010	Uncertain significance (VCV000503538.6)	-
MECOM	Chr3:g.169122674T>C	c.884A>G	p.(Q295R)	NM_004991.4	rs34896995	Missense	25.5	1 | 0	0.4184	Benign (VCV002041744.3)	-
MECOM	Chr3:g.169115629C>T	c.2243G>A	p.(R748Q)	NM_004991.4	rs755819303	Missense	22.4	1 | 0	0.0014	-	-
MECOM	Chr3:g.169143749G>T	c.459C>A	p.(F153L)	NM_004991.4	rs199656825	Missense	21.7	1 | 0	0.0589	-	-
MYB	Chr6:g.135200148C>G	c.1773C>G	p.(F591L)	NM_001130173.2	-	Missense	24.4	1 | 0	**0.0002**	-	-
MYB	Chr6:g.135197012G>T	c.1255G>T	p.(D419Y)	NM_001130173.2	rs774565025	Missense	25.2	1 | 0	0.0014	-	-
NCOR2	Chr12:g.124495200G>A	c.52C>T	p.(R18C)	NM_006312.6	rs369571536	Missense	28.8	1 | 0	0.0318	-	-
NCOR2	Chr12:g.124347842C>T	c.4678G>A	p.(V1560M)	NM_006312.6	rs2229841	Missense	21.4	1 | 0	0.0604	-	-
NCOR2	Chr12:g.124344633C>T	c.4055G>A	p.(R1352H)	NM_006312.6	rs36081651	Missense	26.1	2 | 0	0.2584	-	-
NCOR2	Chr12:g.124372584C>T	c.2245G>A	p.(E749K)	NM_006312.6	-	Missense	26.5	1 | 0	0.0037	-	-
NCOR2	Chr12:g.124486490G>T	c.184C>A	p.(Q62K)	NM_006312.6	-	Missense	25.4	1 | 0	**0.0001**	-	-
NF1	Chr17:g.31225224C>T	c.1975C>T	p.(R659W)	NM_001042492.3	rs757512142	Missense	25.5	1 | 0	0.0017	Uncertain significance (VCV000184227.15)	-
PDGFRB	Chr5:g.150119473T>C	c.2792A>G	p.(D931G)	NM_002609.4	-	Missense	23.2	1 | 0	**0.0002**	-	-
PDS5B	Chr13:g.32687157G>A	c.1227G>A	p.(M409I)	NM_015032.4	rs923273558	Missense	26.0	1 | 0	0.0016	-	-
PHF6	ChrX:g.134413559C>T	c.487C>T	p.(R163C)	NM_001015877.2	rs199945885	Missense	25.7	1 | 0	0.0068	Likely benign (VCV000135031.7)	-
PIGA	ChrX:g.15331636T>G	c.295A>C	p.(N99H)	NM_002641.4	rs1241615240	Missense	25.0	2 | 0	**<0.0001**	-	-
PRPF8	Chr17:1682211C>T	c.352G>A	p.(V118M)	NM_006445.4	-	Missense	25.8	1 | 0	**0.0002**	-	-
PTPN11	Chr12:112454601A>G	c.563A>G	p.(D188G)	NM_002834.5	rs1436513335	Missense	24.7	1 | 0	**0.0001**	Uncertain significance (VCV001422308.5)	-
RAD21	Chr8:g.116852066A>C	c.1352T>G	p.(L451R)	NM_006265.3	rs144953114	Missense	29.0	1 | 0	0.0753	Conflicting Interpretations (VCV000372619.12)	-

SETD2	Chr3:g.47122481T>C	c.2155A>G	p.(N719D)	NM_014159.7	rs115859828	Missense	23.0	1 | 0	0.0031	Conflicting Interpretations (VCV000135211.11)	-
SETD2	Chr3:g.47122861G>T	c.1775C>A	p.(T592K)	NM_014159.7	rs115569620	Missense	23.0	1 | 0	0.0867	Likely benign (VCV000135219.7)	-
SETD2	Chr3:g.47123533C>T	c.1103G>A	p.(R368Q)	NM_014159.7	rs1385695873	Missense	25.8	1 | 0	0.0005	Uncertain significance (VCV000574938.2)	-
THRAP3	Chr1:g.36292601A>G	c.1922A>G	p.(H641R)	NM_005119.4	-	Missense	24.3	1 | 0	**0.0001**	-	-
THRAP3	Chr1:g.36289262C>T	c.1243C>T	p.(R415*)	NM_005119.4	-	Stop gain	35.0	1 | 0	**0.0003**	-	-
UBA1	ChrX:g.47198836G>T	c.34G>T	p.(V12L)	NM_003334.4	rs982155788	Missense	20.6	1 | 0	0.0011	Uncertain significance (VCV001435307.3)	-

AUTOINFLAMMATION GENE PANEL

Gene	g.DNA Nomenclature	c.DNA Nomenclature	Protein Nomenclature	RefSeq transcript	dbSNP	Consequence	CADD	Cases (AOSD | HC)	Fisher's Exact (p-value)	ClinVar	INFEVERS
ALPK1	Chr4:g.112382476A>G	c.200A>G	p.(Q67R)	NM_025144.4	rs33943680	Missense	25.8	1 | 1	-	Likely Benign (VCV000771706.4)	-
ALPK1	Chr4:g.112438573C>G	c.3278C>G	p.(T1093R)	NM_025144.4	rs566719614	Missense	23.6	1 | 0	**0.0001**	-	-
ALPK1	Chr4:g.112438606T>C	c.3311T>C	p.(I1104T)	NM_025144.4	rs375993289	Missense	25.1	1 | 0	0.0054	Uncertain Significance (VCV001917761.2)	-
CARD14	Chr17:g.80205054G>C	c.2418G>C	p.(W806C)	NM_001366385.1	rs2041211247	Missense	25.7	1 | 0	0.0061	Uncertain Significance (VCV000916444.18)	-
CARD14	Chr17:g.80205587G>A	c.2626G>A	p.(E876K)	NM_001366385.1	rs746358733	Missense	25.9	1 | 0	0.0066	-	-
IL36RN	Chr2:g.113062150C>T	c.142C>T	p.(R48W)	NM_012275.3	rs151325121	Missense	24.8	1 | 0	0.0176	Uncertain significance (VCV000030491.10)	Not classified
IL36RN	Chr2:g.113062547C>T	c.338C>T	p.(S113L)	NM_012275.3	rs144478519	Missense	22.5	1 | 2	-	Conflicting Interpretations (VCV000030490.39)	Not classified
LPIN2	Chr18:g.2929105G>A	c.1510C>T	p.(L504F)	NM_001375808.2	rs104895500	Missense	25.8	2 | 0	0.0747	Conflicting Interpretations (VCV000097814.54)	Not classified
MEFV	Chr16:g.3256404C>A	c.184G>T	p.(G62W)	NM_000243.3	-	Missense	25.4	1 | 0	0.0007	-	Not classified
NLRC4	Chr2:g.32238296C>A	c.2357G>T	p.(G786V)	NM_001199138.2	rs149451729	Missense	21.3	2 | 0	0.1119	Conflicting Interpretations (VCV000475252.28)	Likely Benign
NLRP12	Chr19:g.53824146A>G	c.29T>C	p.(L10P)	NM_144687.4	rs775129261	Missense	24.6	1 | 0	0.0015	Uncertain significance (VCV001442965.4)	-
NLRP12	Chr19:g.53795869G>A	c.3088C>T	p.(R1030*)	NM_144687.4	rs201619538	Stop gain	44	1 | 0	0.0041	Uncertain significance (VCV000945830.7)	Uncertain significance
NOD2	Chr16:g.50712175C>T	c.2183C>T	p.(A728V)	NM_001370466.1	rs61747625	Missense	23.5	3 | 0	0.0160	Conflicting Interpretations (VCV000319462.35)	-
NOD2	Chr16:g.50712214A>G	c.2222A>G	p.(K741R)	NM_001370466.1	rs1298126346	Missense	25.0	1 | 0	0.0006	-	-
OTULIN	Chr5:g.14690079A>G	c.635A>G	p.(Q212R)	NM_138348.6	rs369858491	Missense	22.3	1 | 0	0.0458	Uncertain significance (VCV001359906.4)	-
PLCG2	Chr16:g.81905484T>C	c.1444T>C	p.(Y482H)	NM_002661.5	rs187956469	Missense	24.1	3 | 0	0.0149	Conflicting Interpretations (VCV000440158.30)	Likely benign
PSMB4	Chr1:g.151400811C>T	c.542C>T	p.(S181L)	NM_002796.3	rs11557382	Missense	24.2	1 | 0	0.0025	Uncertain significance (VCV001428080.2)	-
PSMG2	Chr18:g.12724566G>A	c.649G>A	p.(A217T)	NM_020232.5	-	Missense	29.3	1 | 0	**0.0001**	-	-

RBCK1	Chr20:g.409908G>A	c.50G>A	p.(R17Q)	NM_031229.4	rs200757913	Missense	22.2	1 | 0	0.0055	Uncertain significance (VCV000858404.4)	-
RELA	Chr11:g.65658293C>T	c.871G>A	p.(D291N)	NM_021975.4	rs61759893	Missense	27.5	1 | 0	0.4424	Benign/Likely benign (VCV000791495.9)	-
RIPK1	Chr6:g.3113257C>T	c.1934C>T	p.(T645M)	NM_001354930.2	rs116040763	Missense	24.3	1 | 0	0.0176	Conflicting Interpretations (VCV000598788.7)	Pathogenic
TNFRSF1A	Chr12:g.6333817C>T	c.242G>A	p.(C81Y)	NM_001065.4	rs104895220	Missense	24.7	1 | 0	0.0004	Not provided (VCV000097669.1)	Pathogenic
TRAP1	Chr16:g.3717505C>A	c.4G>T	p.(A2S)	NM_016292.3	rs748315970	Missense	23.8	1 | 0	0.0784	Uncertain significance (VCV001518112.2)	-
TRAP1	Chr16:g.3674364C>T	c.1019G>A	p.(R340H)	NM_016292.3	rs145715008	Missense	22.7	1 | 0	0.1041	Uncertain significance (VCV000559152.7)	-
TRAP1	Chr16:g.3664360G>A	c.1483C>T	p.(R495C)	NM_016292.3	rs113510154	Missense	31.0	1 | 0	0.0031	-	-

TYPE I INTERFERONOPATHY GENE PANEL

Gene	g.DNA Nomenclature	c.DNA Nomenclature	Protein Nomenclature	RefSeq transcript	dbSNP	Consequence	CADD	Cases (AOSD | HC)	Fisher's Exact (p-value)	ClinVar	INFEVERS
IFIH1	Chr2:g.162277580C>A	c.1879G>T	p.(E627*)	NM_022168.4	rs35744605	Stop gain	37.0	1 | 0	0.5450	Conflicting Interpretations (VCV000377048.31)	-
IFIH1	Chr2:g.162272383C>T	c.2459G>A	p.(R820H)	NM_022168.4	rs74162087	Missense	27.6	1 | 0	0.0873	Conflicting Interpretations (VCV000426581.10)	-
RNASEH2A	Chr19:g.12810201G>A	c.542G>A	p.(C181Y)	NM_006397.3	rs201041092	Missense	27.0	1 | 0	0.0020	Uncertain significance (VCV001428783.4)	-
RNASEH2B	Chr13:g.50945445G>A	c.529G>A	p.(A177T)	NM_024570.4	rs75184679	Missense	24.0	1 | 0	0.2540	Pathogenic/Likely pathogenic (VCV000001262.96)	-
SAMHD1	Chr20:g.36927201C>T	c.677G>A	p.(R226H)	NM_015474.4	rs369489315	Missense	28.4	1 | 0	0.0006	-	-
SKIV2L	Chr6:g.31964081C>T	c.1816C>T	p.(R606C)	NM_006929.5	rs78026291	Missense	28.5	1 | 0	0.0172	Uncertain significance (VCV001064068.4)	-
SKIV2L	Chr6:g.31963970G>A	c.1705G>A	p.(V569M)	NM_006929.5	rs144147284	Missense	24.8	2 | 0	0.0250	Benign/Likely benign (VCV000356328.16)	-
