# Supplementary material for: Characterization of Genetic Landscape and Novel Inflammatory Biomarkers in Patients With Adult‐Onset Still's Disease
Source: Arthritis Rheumatol. 2024 Dec 16;77(5):582–95. doi: 10.1002/art.43054 (PMC12039473; doi:10.1002/art.43054)
Supplement: Supplementary file 15 — Supplemental Table S5. Putative somatic variants identified in cases of adult‐onset Still's disease. Putative somatic variants identified by Mutect2 are colour coded based on the gene panel category: CHIP (orange), Autoinflammatory (green), Type I interferonopathy (blue). Genomic coordinates are provided for the human reference genome build GRCh38. c.DNA nomenclature provided according to the assigned RefSeq transcript. Variants identified by Mutect2 alone, and not HaplotypeCaller, were flagged as putative somatic variants. The interpretation of the remaining variants was based on pre‐existing evidence in COSMIC. Mutations identified by both Mutect2 and HaplotypeCaller, with prior evidence as a somatic mutation were also flagged as putative somatic variants. All other variants were labelled as variants of “Uncertain” origin. VAF % values underlined denote a high value from an X chromosome gene in a male subject. The VAF % and read counts of variants carried by multiple AOSD cases are highlighted # and +. [file ART-77-582-s013.pdf]

Supplemental Table S5. Putative somatic variants identified in cases of adult-onset Still's disease

Gene	g.DNA Nomenclature	c.DNA Nomenclature	Protein Nomenclature	RefSeq transcript	Consequence	CADD	COSMIC ID	VAF %	Reads (MU:WT)	Variant Caller	Interpretation
ANKRD26	Chr10:g.27035332C>G	c.3118G>C	p.(E1040Q)	NM_014915.3	Missense	23.9	-	40.6	33:59	Mutect2, HaplotypeCaller	Uncertain
DNMT3A	Chr2:g.25244579C>A	c.1628G>T	p.(G543V)	NM_022552.5	Missense	25.6	COSV53045034	3.7	7:246	Mutect2	**Putative somatic**
DNMT3A	Chr2:g.25240715G>C	c.2098C>G	p.(P700A)	NM_022552.5	Missense	26.9	COSV53043215	1.3	8:648	Mutect2	**Putative somatic**
DNMT3A	Chr2:g.25244622C>T	c.1585G>A	p.(D529N)	NM_022552.5	Missense	27.0	-	15.0	31:179	Mutect2, HaplotypeCaller	Uncertain
DNMT3A	Chr2:g.25247647G>A	c.958C>T	p.(R320*)	NM_022552.5	Stop gain	37.0	COSV53041342	13.5	8:61	Mutect2, HaplotypeCaller	**Putative somatic**
EZH2	Chr7:g.148828742T>C	c.623A>G	p.D208G	NM_004456.5	Missense	25.3	-	65.2	69:33	Mutect2, HaplotypeCaller	Uncertain
FAT4	Chr4:g.125491302G>C	c.14486G>C	p.(R4829T)	NM_001291303.3	Missense	22.6	-	46.7	102:122	Mutect2, HaplotypeCaller	Uncertain
FAT4	Chr4:g.125317489G>A	c.1078G>A	p.(A360T)	NM_001291303.3	Missense	22.6	-	1.3	5:511	Mutect2	**Putative somatic**
FAT4	Chr4:g.125398903G>C	c.5295G>C	p.(M1765I)	NM_001291303.3	Missense	21.2	-	38.1	41:75	Mutect2, HaplotypeCaller	Uncertain
KMT2A	Chr11:g.118436816G>A	c.304G>A	p.G102R	NM_001197104.2	Missense	24.0	-	52.6	32:43	Mutect2, HaplotypeCaller	Uncertain
KMT2A	Chr11:g.118505418T>A	c.9526T>A	p.(F3176I)	NM_001197104.2	Missense	27.1	-	5.8	9:151	Mutect2	**Putative somatic**
KMT2D	Chr12:g.49024662C>A	c.15968G>T	p.(R5323L)	NM_003482.4	Missense	28.7	-	44.7	84:107	Mutect2, HaplotypeCaller	Uncertain
MECOM	Chr3:g.169115629C>T	c.2243G>A	p.(R748Q)	NM_004991.4	Missense	22.4	COSV52958497	37.2	40:62	Mutect2, HaplotypeCaller	**Putative somatic**
MEFV	Chr16:g.3256404C>A	c.184G>T	p.(G62W)	NM_000243.3	Missense	25.4	-	50.1	79:78	Mutect2, HaplotypeCaller	Uncertain
NLRP12	Chr19:g.53824146A>G	c.29T>C	p.(L10P)	NM_144687.4	Missense	24.6	-	57.4	73:51	Mutect2, HaplotypeCaller	Uncertain
PIGA	ChrX:g.15331636T>G	c.295A>C	p.(N99H)	NM_002641.4	Missense	25.0	-	43.1[#]<br>99.0[+]	83:114[#]<br>0:123[+]	Mutect2, HaplotypeCaller	Uncertain
PTPN11	Chr12:g.112486520C>T	c.1270C>T	p.(P424S)	NM_002834.5	Missense	26.3	COSV100692718	4.3	3:76	Mutect2	**Putative somatic**
