# Supplementary material for: Characterization of Genetic Landscape and Novel Inflammatory Biomarkers in Patients With Adult‐Onset Still's Disease
Source: Arthritis Rheumatol. 2024 Dec 16;77(5):582–95. doi: 10.1002/art.43054 (PMC12039473; doi:10.1002/art.43054)
Supplement: Supplementary file 16 — Supplemental Table S6. Candidate variants from transcriptomics data. 87 rare (<1% population allele frequency), potentially pathogenic (CADD>20) germline variants were identified across 61/170 genes, shared between the top three pathways enriched in the transcriptome data. The incidence of each variant was compared to their prevalence recorded in the European (Non‐Finnish) population within gnomAD v4.0.0, and statistically significant enrichment is underlined in red (defined by adjusted p‐value <0.0004 after multiple testing correction). Genomic coordinates are provided for the human reference genome build GRCh38. c.DNA nomenclature provided according to the selected RefSeq transcript. Cases carrying homozygous variants are highlighted B. [file ART-77-582-s014.pdf]

SUPPLEMENTAL TABLE S6. Candidate variants from transcriptomics data

Gene	g.DNA Nomenclature	c.DNA Nomenclature	Protein Nomenclature	RefSeq transcript	dbSNP	Consequence	CADD	Cases (AOSD | HC)	Fisher's Exact (p-value)	ClinVar (Accession)
ADGRE5	Chr19:g.14406401G>A	c.1892G>A	p.(S631N)	NM_078481.4	rs140048136	Missense	22.7	1 | 0	0.0311	-
AGPAT2	Chr9:g.136674749T>A	c.647A>T	p.(K216M)	NM_006412.4	rs143244920	Missense	24.9	1[B] | 0	0.0024	Conflicting interpretations (VCV000913447.9)
AGPAT2	Chr9:g.136674750T>A	c.646A>T	p.(K216*)	NM_006412.4	rs138994150	Stop gain	46.0	1[B] | 0	0.0028	Conflicting interpretations (VCV000365922.17)
AGPAT2	Chr9:g.136677510G>A	c.229C>T	p.(R77C)	NM_006412.4	rs151053652	Missense	26.2	1 | 0	0.0026	Uncertain significance (VCV000128289.7)
AIFM2	Chr10:g.70120545T>C	c.469A>G	p.(M157V)	NM_032797.6	rs139479025	Missense	23.5	1 | 0	0.2927	-
ANPEP	Chr15:g.89803917G>C	c.1265C>G	p.(A422G)	NM_001150.3	rs144282919	Missense	27.5	1 | 0	0.214	Likely benign (VCV000716129.4)
ANPEP	Chr15:g.89806367G>A	c.217C>T	p.(R73C)	NM_001150.3	rs200298670	Missense	22.7	1 | 0	0.0663	-
ANPEP	Chr15:g.89785450G>A	c.2803C>T	p.(R935W)	NM_001150.3	rs141333670	Missense	32.0	1 | 0	0.1378	-
ANPEP	Chr15:g.89804345G>A	c.1087C>T	p.(R363W)	NM_001150.3	rs147069406	Missense	28.2	1 | 0	0.017	-
APAF1	Chr12:g.98699432G>A	c.2329G>A	p.(E777K)	NM_181861.2	rs138526583	Missense	21.6	2 | 0	0.1274	-
ARHGAP9	Chr12:g.57474102G>A	c.1858C>T	p.(R620W)	NM_032496.4	rs61758883	Missense	27.6	1 | 0	0.4362	-
ARHGAP9	Chr12:g.57477605G>A	c.610C>T	p.(P204S)	NM_032496.4	rs112573859	Missense	23.0	1 | 0	0.3927	-
ASAH1	Chr8:g.18057578C>T	c.1144G>A	p.(E382K)	NM_177924.5	rs148976489	Missense	25.4	1 | 0	0.0264	Uncertain significance (VCV000911466.12)
ASAH1	Chr8:g.18061398G>C	c.764C>G	p.(T255R)	NM_177924.5	-	Missense	20.2	1 | 0	0.0001	-
ATAD3B	Chr1:g.1495809_1495810del	c.1939_1940del	p.(p.L647Vfs*6)	NM_031921.6	-	Frameshift	21.2	1 | 0	0.0003	-
ATG7	Chr3:g.11348028C>T	c.1277C>T	p.(P426L)	NM_001349232.2	rs143545741	Missense	25.5	1 | 0	0.1697	-
BPI	Chr20:g.38326269C>T	c.998C>T	p.(A333V)	NM_001725.3	rs142171209	Missense	20.5	1 | 0	0.0151	-
C3	Chr19:g.6678019T>G	c.4855A>C	p.(S1619R)	NM_000064.4	rs2230210	Missense	22.1	1 | 0	0.2598	Conflicting interpretations (VCV000330271.33)

C3	Chr19:g.6707118G>A	c.2203C>T	p.(R735W)	NM_000064.4	rs117793540	Missense	22.6	1 | 0	0.2607	-
C3AR1	Chr12:g.8060072C>A	c.114G>T	p.(L38F)	NM_004054.4	-	Missense	23.2	1 | 0	0.0002	-
CANT1	Chr17:g.78997190C>A	c.433G>T	p.(V145L)	NM_001159773.2	rs140112462	Missense	23.0	1 | 0	0.0188	Conflicting interpretations (VCV000325703.13)
CAP1	Chr1:g.40067685C>T	c.776C>T	p.(A259V)	NM_006367.4	rs148169899	Missense	26.4	1 | 0	0.0449	-
CD36	Chr7:g.80664445G>A	c.649G>A	p.(G217R)	NM_001001548.3	rs200067322	Missense	32.0	1 | 0	0.0243	Uncertain significance (VCV000360761.8)
CD36	Chr7:g.80674138_80674147del	c.1416_*1del	p.(L472Nfs*80)	NM_001001548.3	rs771061715	Frameshift	29.3	1 | 0	0.0005	Likely pathogenic (VCV000692066.3)
CEACAM6	Chr19:g.41762212T>A	c.947T>A	p.(I316N)	NM_002483.7	rs149127408	Missense	20.7	2 | 1	-	-
CEP290	Chr12:g.88086456C>G	c.4237G>C	p.(D1413H)	NM_025114.4	rs183655276	Missense	23.4	1 | 0	0.2979	Conflicting interpretations (VCV000096172.41)
CEP290	Chr12:g.88111221T>C	c.2348A>G	p.(Y783C)	NM_025114.4	rs780990839	Missense	29.7	1 | 0	0.0011	Uncertain Significance (VCV000880839.9)
CHI3L1	Chr1:g.203185337C>T	c.104G>A	p.(R35Q)	NM_001276.4	rs146010120	Missense	28.2	1 | 0	0.1418	-
CKAP4	Chr12:g.106239035C>T	c.1798G>A	p.(E600K)	NM_006825.4	rs773178493	Missense	26.7	1 | 0	0.0041	-
COMMD9	Chr11:g.36276228C>T	c.365G>A	p.(R122H)	NM_014186.4	rs374847668	Missense	23.9	1 | 0	0.0008	-
CPPED1	Chr16:g.12705025G>A	c.314C>T	p.(T105M)	NM_018340.3	rs186670586	Missense	22.5	1 | 0	>0.9999	-
CR1	Chr1:g.207588702C>A	c.5738C>A	p.(P1913H)	NM_000651.6	-	Missense	21.3	1 | 0	0.0001	-
CRACR2A	Chr12:g.3633629G>C	c.1710C>G	p.(F570L)	NM_001144958.2	-	Missense	24.2	1 | 0	0.0001	-
CRACR2A	Chr12:g.3659602G>T	c.724C>A	p.(Q242K)	NM_001144958.2	-	Missense	25.5	1 | 0	0.0001	-
CRACR2A	Chr12:g.3656325G>C	c.844C>G	p.(Q282E)	NM_001144958.2	rs1445855890	Missense	23.9	1 | 0	0.0008	-
CRISPLD2	Chr16:g.84867039G>A	c.852G>A	p.(M284I)	NM_031476.4	rs149615348	Missense	24.5	1 | 1	-	-
CRISPLD2	Chr16:g.84838721A>G	c.226A>G	p.(N76D)	NM_031476.4	rs201061137	Missense	22.7	1 | 0	0.0298	-

CST3	Chr20:g.23635325G>C	c.286C>G	p.(R96G)	NM_000099.4	rs11542355	Missense	23.7	1 | 0	0.0003	-
CTSA	Chr20:g.45894833G>A	c.880G>A	p.(D294N)	NM_000308.4	rs142892564	Missense	22.7	2 | 0	0.1099	Benign/Likely benign (VCV000624194.30)
CTSA	Chr20:g.45895081G>A	c.1036G>A	p.(V346M)	NM_000308.4	rs137941635	Missense	24.9	1 | 0	0.0011	Conflicting interpretations (VCV000338536.15)
CTSA	Chr20:g.45894665C>T	c.793C>T	p.(R265C)	NM_000308.4	rs763381306	Missense	21.4	1 | 0	0.0068	Uncertain Significance (VCV000338533.6)
CTSB	Chr8:g.11847697T>C	c.658A>G	p.(K220E)	NM_001908.5	rs371200657	Missense	20.3	1 | 0	0.0033	-
CTSB	Chr8:g.11847084G>C	c.761C>G	p.(S254C)	NM_001908.5	rs200664537	Missense	22.9	1 | 0	0.0162	-
CXCR2	Chr2:g.218135544G>A	c.743G>A	p.(R248Q)	NM_001557.4	rs61733609	Missense	24.6	1 | 0	0.1155	-
DOCK2	Chr5:g.170067575G>T	c.4533G>T	p.(M1511I)	NM_004946.3	rs148502872	Missense	22.8	1 | 0	0.3447	Likely benign (VCV000542638.8)
DOK3	Chr5:g.177504852G>A	c.536C>T	p.(P179L)	NM_001308236.3	rs765323088	Missense	22.5	1 | 0	0.0368	-
EGFL7	Chr9:g.136668600G>A	c.124G>A	p.(E42K)	NM_016215.5	rs143961691	Missense	25.4	2 | 0	<0.0001	-
FRMPD3	Chrx:g.107602115C>T	c.4076C>T	p.(S1359F)	NM_001388459.1	rs187380964	Missense	24.9	1 | 0	0.1066	-
FRMPD3	Chrx:g.107602480G>A	c.4441G>A	p.(E1481K)	NM_001388459.1	rs776277144	Missense	22.9	1 | 0	0.0008	-
GAA	Chr17:g.80107818G>A	c.877G>A	p.(G293R)	NM_000152.5	rs121907945	Missense	32.0	1 | 0	0.0009	-
GCA	Chr2:g.162352367G>T	c.222G>T	p.(Q74H)	NM_012198.5	rs79565841	Missense	25.2	1 | 0	0.3924	-
GGH	Chr8:g.63024114G>A	c.572C>T	p.(T191I)	NM_003878.3	-	Missense	27.7	1 | 0	0.0002	-
GPR84	Chr12:g.54362896C>T	c.956G>A	p.(R319Q)	NM_020370.3	rs764900108	Missense	22.1	1 | 0	0.0064	-
IQGAP1	Chr15:g.90483498G>A	c.3693G>A	p.(M1231I)	NM_003870.4	rs117246983	Missense	22.2	1 | 0	0.3183	-
LCN2	Chr9:g.128149551G>T	c.26G>T	p.(G9V)	NM_005564.5	rs147787222	Missense	21.9	1 | 1	-	Likely Benign (VCV000780048.3)
LGALS3	Chr14:g.55145237T>C	c.719T>C	p.(I240T)	NM_002306.4	rs150161752	Missense	24.4	1 | 0	0.0174	-

LTA4H	Chr12:g.96003916G>C	c.1535C>G	p.(P512R)	NM_000895.3	rs771917557	Missense	24.8	1 | 0	0.0096	-
LTF	Chr3:g.46445289C>T	c.1505G>A	p.(C502Y)	NM_002343.6	rs1462341921	Missense	27.8	1 | 0	0.0001	-
MGAM	Chr7:g.142032859C>T	c.1619C>T	p.(S540L)	NM_001365693.1	rs201161396	Missense	26.0	1 | 0	0.0173	-
MGAM	Chr7:g.142027650G>A	c.1136G>A	p.(G379E)	NM_001365693.1	rs191269779	Missense	27.2	1 | 0	0.0103	-
MMP9	Chr20:g.46010960C>T	c.559C>T	p.(L187F)	NM_004994.3	rs55789927	Missense	28.2	1 | 0	0.1624	Benign/Likely benign (VCV000773609.14)
MMP9	Chr20:g.46010575C>T	c.464C>T	p.(T155I)	NM_004994.3	rs143024943	Missense	22.8	1 | 0	0.0005	Uncertain significance (VCV000338547.8)
MMP9	Chr20:g.46011636G>A	c.886G>A	p.(G296S)	NM_004994.3	rs144098289	Missense	25.5	1 | 1	-	Conflicting interpretations (VCV000198179.17)
MNDA	Chr1:g.158847794G>A	c.1054G>A	p.(G352R)	NM_002432.3	rs369069905	Missense	22.6	1 | 0	0.002	-
MPO	Chr17:g.58270865T>G	c.2031-2A>C	p.?	NM_000250.2	rs35897051	Splice acceptor	34.0	1 | 0	0.5492	-
MPO	Chr17:g.58277971G>A	c.1060C>T	p.(R354C)	NM_000250.2	-	Missense	24.6	1 | 0	0.0002	-
NPC2	Chr14:74480701C>T	c.441+1G>A	p.?	NM_006432.5	rs140130028	Splice donor	23.2	1 | 1	-	Conflicting interpretations (VCV000100734.72)
PGM1	Chr1:g.63651802G>A	c.1414G>A	p.(D472N)	NM_002633.3	rs774591034	Missense	29.9	1 | 0	0.0027	-
PLAU	Chr10:g.73915328T>C	c.1048T>C	p.(Y350H)	NM_002658.6	rs72816325	Missense	25.0	1 | 1	-	Benign/Likely benign (VCV000300758.9)
PRCP	Chr11:g.82849966A>C	c.699T>G	p.(C233W)	NM_005040.4	rs1162309389	Missense	25.9	1 | 0	0.0012	-
PYGL	Chr14:g.50944180T>C	c.224A>G	p.(Y75C)	NM_002863.5	rs771094581	Missense	32.0	1 | 0	0.007	-
RAB5C	Chr17:g.42128371G>A	c.331C>T	p.(R111W)	NM_004583.4	-	Missense	33.0	1 | 0	0.0002	-
RHOA	Chr3:g.49362504T>A	c.400A>T	p.(M134L)	NM_001664.4	rs779873892	Missense	23.0	1 | 0	0.0035	-
SIGLEC5	Chr19:g.51627722G>C	c.1022C>G	p.(S341C)	NM_003830.4	rs574831178	Missense	21.1	1 | 0	0.0369	-
SLC44A2	Chr19:g.10637706G>C	c.1654G>C	p.(E552Q)	NM_020428.4	rs142741358	Missense	27.2	2 | 0	0.2	-

TMBIM1	Chr2:g.218282119G>C	c.23C>G	p.(P8R)	NM_022152.6	rs146727797	Missense	23.8	1 | 0	0.1782	-
TMC6	Chr17:g.78124676A>G	c.739T>C	p.(S247P)	NM_001127198.5	-	Missense	28.1	1 | 0	0.0004	-
TMC6	Chr17:g.78121702C>G	c.1237G>C	p.(A413P)	NM_001127198.5	rs555453763	Missense	24.3	1 | 0	0.0365	Conflicting interpretations (VCV000788265.8)
TMC6	Chr17:g.78125168G>A	c.526C>T	p.(R176C)	NM_001127198.5	rs112743475	Missense	23.5	1 | 0	0.0068	Conflicting interpretations (VCV001122634.6)
TNFAIP6	Chr2:g.151363949C>A	c.101C>A	p.(A34E)	NM_007115.4	-	Missense	25.3	1 | 0	0.0008	-
TOM1	Chr22:g.35334417C>T	c.1117C>T	p.(R373W)	NM_005488.3	rs377303679	Missense	28.5	1 | 0	0.0072	-
VAPA	Chr18:g.9954087C>T	c.626C>T	p.(S209L)	NM_194434.3	rs139402315	Missense	22.5	1 | 0	0.0048	-
VCL	Chr10:g.74090140C>G	c.1294C>G	p.(L432V)	NM_014000.3	rs144146254	Missense	22.3	1 | 0	0.0482	Conflicting interpretations (VCV000166543.27)
VCL	Chr10:g.73998227G>T	c.20G>T	p.(R7L)	NM_014000.3	-	Missense	25.9	1 | 0	0.0052	-
VCL	Chr10:g.74100982A>G	c.1907A>G	p.(H636R)	NM_014000.3	rs71579374	Missense	24.1	2 | 0	0.0124	Conflicting interpretations (VCV000045594.42)
VNN1	Chr6:g.132711835G>A	c.215C>T	p.(A72V)	NM_004666.3	rs146663285	Missense	24.3	1 | 0	0.0384	-
VNN1	Chr6:g.132713848G>A	c.188C>T	p.(A63V)	NM_004666.3	rs45500693	Missense	24.9	1 | 0	0.3527	-
